# The ambrosial mycobiota of *Treptoplatypus oxyurus* (*Coleoptera*, *Platypodidae*): a unique island of fungal diversity revealing *Wilhelmdebeerea oxyuri* gen. et sp. nov. (*Ophiostomatales*), and two new yeast species *Blastobotrys sasensis* sp. nov., and *Sugiyamaella casensis* sp. nov. (*Dipodascales*)

**DOI:** 10.3897/imafungus.17.177075

**Published:** 2026-02-16

**Authors:** Miroslav Kolařík, Renata Vadkertiová, Miloš Knížek, František Sklenář, Jozef Vakula, Milan Zúbrik, Michal Kolář, Jiří Hulcr

**Affiliations:** 1 Institute of Microbiology of the Czech Academy of Sciences, Vídeňská 1083, Praha 14220, Czech Republic Institute of Microbiology of the Czech Academy of Sciences Praha Czech Republic; 2 Culture Collection of Yeasts, Institute of Chemistry, Slovak Academy of Sciences, Dúbravská cesta 9, Bratislava 84538, Slovakia School of Forest, Fisheries and Geomatics Sciences, University of Florida Gainesville United States of America; 3 Department of Forest Protection Service, Forestry and Game Management Research Institute, Strnady 136, Jíloviště 25202, Czech Republic Department of Forest Protection Service, Forestry and Game Management Research Institute Jíloviště Czech Republic; 4 Department of Forest Protection and Forest Protection Service, Science and Research Division, National Forest Centre, Lesnícka 11, 969 01 Banská Štiavnica, Slovakia Culture Collection of Yeasts, Institute of Chemistry, Slovak Academy of Sciences Bratislava Slovakia; 5 Institute of Molecular Genetics of the Czech Academy of Sciences, Vídeňská 1083, Praha 14220, Czech Republic Institute of Molecular Genetics of the Czech Academy of Sciences Praha Czech Republic; 6 School of Forest, Fisheries and Geomatics Sciences, University of Florida, Gainesville 32611, Florida, USA Science and Research Division, National Forest Centre Banská Štiavnica Slovakia

**Keywords:** ambrosia beetles, ambrosia fungi, *Candida schatavii*, *
Magnusiomyces
*, mycangia, pinhole borers, symbiosis

## Abstract

Ambrosia beetles (*Coleoptera*, *Curculionidae*) form obligate nutritional symbioses with ambrosia fungi cultivated within their galleries. Among them, the pinhole borers (*Platypodinae*) are predominantly tropical, with only two representatives native to Europe. One of them, the rare and understudied *Treptoplatypus oxyurus*, primarily colonises *Abies alba*. We investigated its fungal symbionts using a cultivation-dependent approach. We identified three numerically dominant associates in the prothorax containing mycangia: *Candida schatavii*, *Magnusiomyces fungicola*, and a novel member of *Ophiostomatales*. The latter, *Wilhelmdebeerea oxyuri***gen. et sp. nov**., was the most abundant and exhibited both leptographium-like and hyalorhinocladiella-like morphs. Additionally, two new yeast species of low abundance and uncertain ecological roles were isolated and described: *Blastobotrys sasensis***sp. nov**. and *Sugiyamaella casensis***sp. nov**., both belonging to the family *Trichomonascaceae (Dipodascales)*. Multigene and phylogenomics analyses confirmed the distinct taxonomic placement of all three new species. The ecological roles of the identified fungi and the strength of their association with *T. oxyurus* require confirmation through further studies at additional locations. Our findings reveal a previously undocumented fungal diversity tightly linked to a unique pinhole borer, *T. oxyurus*, thereby enriching our understanding of the fungi associated with conifer-colonising beetles and their ecological and biotechnological importance.

## Introduction

Ambrosia beetles are a diverse group of wood-boring weevils (*Coleoptera*, *Curculionidae*) that occur in a strict symbiosis with the so-called ambrosia fungi within their galleries, and which serve as their primary food source. The beetle-fungus consortium plays many roles in tree health and wood decomposition, ranging from contributing to tree death to facilitating the entry of other organisms into the wood resource, and it also dominates the subcortical fungal community and sometimes delays wood decay (Skelton et al. 2019; Seibold et al. 2021). The most remarkable diversity of ambrosia beetles is found within the weevil subfamilies *Scolytinae* (6000 spp.) and *Platypodinae* (1400 spp.) (Kirkendall et al. 2015). The subfamily *Platypodinae*, also known as pinhole borers, are primarily distributed in tropical and subtropical regions around the world and fewer than 10 species are found in wet temperate areas (Wood 1993; Jordal 2015). Several species are economically important, as they penetrate felled timber together with their associated fungi and stain the wood (Browne 1968). Their most significant economic impact is in climate-stressed forests, where several species of *Platypus* and *Crossotarsus* can erupt in outbreaks and overwhelm the stressed host trees, in association with their weakly pathogenic fungal symbionts in the genera *Dryadomyces* and *Raffaelea* (*Ascomycota*, *Ophiostomatales*) (Kubono and Ito 2002; Alfaro et al. 2007; Belhoucine et al. 2011; Ceriani-Nakamurakare et al. 2016; Choi et al. 2022).

*Platypodinae* have entered the symbiosis at least 80 million years ago (Jordal and Cognato 2012), much earlier than the *Scolytinae* and other fungus-farming insects (Jordal 2015). This makes them the oldest known fungus-farming system. Symbiotic fungi have only been studied in a negligible portion of platypodine species. The genera *Dryadomyces*, and *Raffaelea* are considered the primary ambrosia fungi, with a long history of coevolution. Many other fungi are frequently isolated from platypodines, but their specificity and ecological role is poorly understood (Attonaty et al. 2024). Both individual mycangia and galleries are inhabited by multiple fungal species, among them the yeasts from the subphylum *Saccharomycotina* are generally abundant. Across a range of studies, genera such as *Ambrosiozyma*, *Candida*, *Meyerozyma*, *Ogataea*, *Saccharomycopsis*, *Sugiyamaella*, and *Trigonopsis* have been reported (Endoh et al. 2011; Yun et al. 2015; Ceriani-Nakamurakare et al. 2020; Meštrović 2023; Rodrigues et al. 2023). Several yeast strains can utilise lignocellulosic sugars, making them promising candidates for fermentative ethanol production from wood waste (Makopa et al. 2024; Subudhi et al. 2024).

Two native platypodine species, *Platypus cylindrus* and *Treptoplatypus oxyurus*, and one non-native species, *Megaplatypus mutatus*, are present in Europe. *Platypus cylindrus* is nearly universally a secondary borer in freshly dead oaks. Still, it can erupt into outbreaks and contribute to tree mortality in oak stands stressed by drought and mechanical damage (Sousa and Inacio 2005). The invasive *Megaplatypus mutatus* was introduced from South America and has caused mortality in poplars in Italy (Kirkendall and Faccoli 2010). *Treptoplatypus oxyurus* is a rare beetle, found in the French Pyrenees (type locality), Spain, Corsica, Sardinia, southern Italy, Croatia, Greece, India, and possibly Turkey and Iran. It is scarce in Central Europe, occurring in Slovakia and Germany (Kubinec 1983; Gogola 1986; Böhme 1994). This species has a specialised ecology, developing exclusively in large dead trunks of *Abies* spp. in natural forests, and may be concentrated in windthrow gaps, habitats usually absent in managed forests (Žarković et al. 2022).

The mycobiota of *P. cylindrus* and *M. mutatus* is well documented. It is dominated by *Dryadomyces montetyi*, *Raffaelea ambrosiae*, and *Raffaelea quercina* in *P. cylindrus*. Symbionts of *M. mutatus* remain poorly characterised, as the mycangium has yet to be located and sampled. Four different *Raffaelea* have been reported (Ceriani-Nakamurakare et al. 2024), and various species of *Fusarium* and *Graphium* have been isolated from the beetle surface (Attonaty et al. 2024). The mycobiota associated with *T. oxyurus* has been studied using material from France (Cassier et al. 1996) and Croatia (Meštrović 2023). Cassier et al. (1996) reported two unidentified fungal species from the mycangia. Meštrović et al. (2023) identified two yeast species, *Meyerozyma guilliermondii*, a tentative main nutritional fungus, and *Magnusiomyces fungicola*, together with *Graphilbum fragrans* as the principal symbionts.

In this study, we present the results of the most comprehensive and quantitative mycological analysis of the *T. oxyurus* mycobiome, based on samples collected in Slovakia. We provide a description of a new genus and species of the most abundant mycangial symbiont, as well as two novel yeast species.

## Methods

### Sampling and fungal isolation

**Figure 1.**
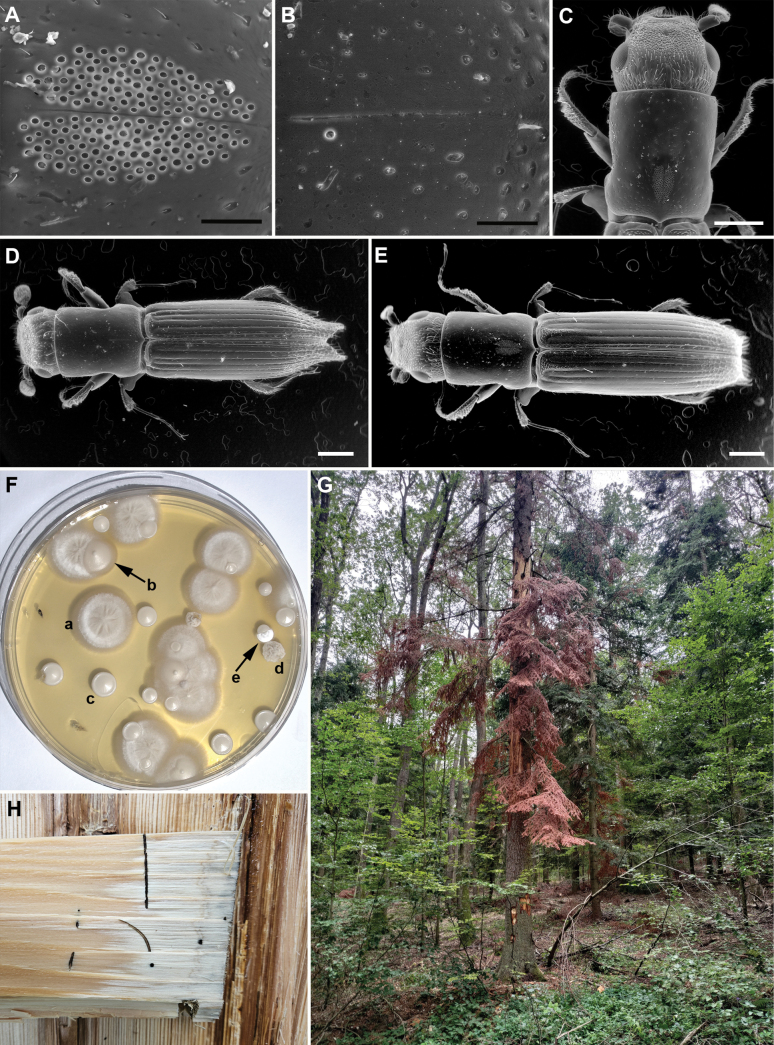
Morphology, habitat and associated fungi of *Treptoplatypus oxyurus*. **A**. Female mycangia. **B**. Male mycangia. **C**. Detail of the female. **D**. Male. **E**. Female. **F**. Malt extract agar plate showing cultivation from a solution obtained from female mycangia: a. *Wilhelmdebeerea oxyuri*, b. *Magnusiomyces fungicola*, c. *Candida schatavii*, d. *Blastobotrys sasensis*, e. *Sugiyamaella casensis*. **G**. Colonised *Abies alba* tree. **H**. Active larval gallery. Scale bars: 10 µm (**A, B**); 500 µm (**C–E**).

A lower trunk section (0.5–3 m above ground, 40 cm in diameter) was collected from a standing, recently dead *Abies alba* tree colonised by *T. oxyurus* (probable colonisation: July–August 2023). The collection took place on October 12, 2023, in Tŕnie near Zvolen, Slovakia (48.627397°N, 19.029675°E). The log was transported to Banská Bystrica and kept outdoors to allow further development. In June 2024, it was transferred to a laboratory in Prague. The wood was in a relatively advanced stage of decay, with noticeable infestation by wood-rotting (white-rot) fungi and significant weight loss. Teneral adults, larvae, and gallery fragments of *T. oxyurus* were excised from the wood, and additional adults, freshly emerging from the wood, were captured. The beetles were identified according to Pfeffer (1995). Their morphology was documented using scanning electron microscopy (SEM) following Kolařík and Vohník (2018) (Fig. 1).

Three types of specimens were used: (1) crushed thoraces of female adults containing mycangia, (2) larvae, and (3) active galleries, i.e. parts of galleries containing feeding larvae. The thorax without digestive tube, aseptically crushed using forceps, living larvae, or gallery segments (1 × 1 mm, containing weed and adjacent ambrosia layer) were vortexed for 2 min in 1 × PBS solution, and the resulting suspension was plated onto Malt Extract Agar (MEA; HiMedia, Mumbai, India; composition: malt extract 20 g, glucose 20 g, peptone 1 g, agar 20 g, H_2_O 1 L) with 10×, 100×, and 1000 × serial dilutions, and incubated for 7–21 days at 25 °C. Alternatively, crushed thoraces, larvae, or gallery fragments were streaked directly onto MEA plates without dilution, as recommended by Batra (1985). See Table 1 for the number of analysed specimens.

**Table 1.** Relative frequency of fungi isolated from larva surface, female thoraxes with mycangia, and walls of active galleries. The values indicate the relative number of CFUs obtained in a particular sample (e.g., a single larva) and are given as (min) mean (max). For instance, *C. schatavii* accounted for between 13% and 92% of all the CFUs obtained from larvae. The total number of larvae was 12.

Fungus	larva (n = 12)	female thorax (includes mycetangia) (n = 12)	active gallery (n = 5)
*Blastobotrys sasensis*	0	(0) 5 (7)	0
*Candida schatavii*	(13) 71 (92)	(11) 33 (53)	(18) 21 (24)
*Magnusiomyces fungicola*	(4) 12 (31)	(7) 10 (16)	(38) 44 (48)
*Ophiostoma pityokteinis*	(1) 1 (1)	0	(4) 6 (6)
*Sugiyamaella casensis*	0	(0) 2 (7)	0
*Wilhelmdebeerea oxyuri*	(3) 16 (56)	(37) 50 (74)	(25) 28 (29)

The fungal colonies obtained were assigned to morphotypes based on colony morphology and microscopy. Representatives of each morphotype were subcultured and identified using ITS barcoding. The DNA was isolated from 1–3-day-old colonies grown on MEA following the methods described by Kolařík et al. (2023). PCR and sequencing were performed using the primers ITS1F/ITS4, as outlined by Kolařík et al. (2017). Identification was based on morphological features and the comparison of ITS barcode sequences with reference sequences deposited in the NCBI database. Representative monosporic strains were deposited in the Culture Collection of Fungi (CCF, Department of Botany, Faculty of Science, Charles University, Prague, Czechia) and the Culture Collection of Yeasts (CCY, Institute of Chemistry, Slovak Academy of Sciences, Bratislava, Slovakia) (Table 1). Holotype specimens were deposited in the National Museum Herbarium (PRM, Prague, Czechia) and in the CCY.

### Morphological, physiological and biochemical characterisation

For the ophiostomatoid fungus CCF 6802, cultures were grown on MEA at 15 °C and 25 °C, with or without autoclaved host tree twigs in order to induce ascocarp or synnemata formation. Microscopic preparations were made in water, and observed using an Axio Scope.A1 microscope (Carl Zeiss, Jena, Germany) under differential interference contrast (DIC) illumination. The ontogeny of conidia was observed using glass slide cultures (Cole et al. 1969). Measurements were made from photographs taken with a ProgRes SpeedXTcore 5 digital camera (Jenoptik Optical Systems, Jena, Germany) using PROGRES image-processing software. Thirty measurements were made for each taxonomically relevant structure. Averages, ranges, and standard deviations were calculated for the measurements and are presented as (min–)mean ± SD(–max). Growth characteristics were determined in three replicates after 14 days of cultivation at 5, 10, 15, 20, 25, 30, 35, and 37 °C, in the dark, on MEA. Agar disks (2 mm diam.) were cut from actively growing colony margins and placed in the centre of the plate. Cycloheximide tolerance was tested on MEA supplemented with 100, 250, and 500 mg/L cycloheximide (Sigma-Aldrich Corp.).

The characteristics of the yeasts were determined using the methods described by Kurtzman et al. (2011). The micromorphology was studied using an Eclipse 80i microscope (Nikon, Japan), and images were captured using a Sony C-IMX250 colour camera (Japan). Sexual reproduction was observed on Malt Extract Agar (MEA; Merck, Germany), Corn Meal Agar (CMA; Merck, Germany), and Yeast Malt Agar (YMA; 3 g yeast extract, 3 g malt extract, 5 g peptone, 10 g glucose, 20 g agar, and 1 L H_2_O) at regular intervals for up to 21 days. Assimilation tests were performed using both liquid and solid Yeast Nitrogen Base (YNB) and Yeast Carbon Base (YCB) (Biolife, Milano, Italy). In the liquid media, yeasts were grown aerobically at 28 °C on a shaker (100 rpm). The cell biomass was measured by its absorbance (660 nm) at regular intervals for a period of 21 days. The absorbance of strains grown in the presence of carbon and nitrogen compounds was compared to that of strains grown in a solution without these substances (control). Assimilation on the solid media was performed using 24-well plates: the respective medium containing a carbon or nitrogen compound was inoculated with 5 µL of the yeast suspension (10^7^ cells/mL). The yeasts were grown at their optimal temperature (28 °C) for 21 days. The carbon and nitrogen compounds (Merck, Germany) were tested at a concentration of 1%. The assimilation of nitrite was tested in a concentration of 0.25% KNO_2_. See Supplementary material 13 for the list of substrates tested. The yeasts were also inoculated on those media without carbon and nitrogen compounds (control). Citrate assimilation was tested using Christensen’s citrate medium (pH 6.7), consisting of 0.5 g yeast extract, 0.1 g l-cysteine hydrochloride, 3 g sodium citrate, 0.2 g dextrose, 1 g monopotassium phosphate, 5 g sodium chloride, 0.012 g phenol red, and 15 g agar per 1 L of deionised water. Cadaverine assimilation was tested at a 4 mM concentration in YCB (pH 5.6). The growth of yeasts was tested at 5, 10, 20, 28, 30, 35 and 37 °C. A temperature of 28 °C was determined to be the most suitable for both yeast cultures.

### Whole genomic sequencing

Chromosomal DNA was isolated using Quick-DNA Fungal/Bacterial Kits (Zymo Research, California, USA). Library preparation (2 × 300 bp, Illumina paired-end) was performed, and sequencing was done on a NextSeq 2000 instrument (Illumina, 100x coverage) following the manufacturer’s protocol at the Genomics and Bioinformatics Core Facility at the Institute of Molecular Genetics of the CAS. The quality of the raw sequencing data was assessed using FASTQC v. 0.11.9 (Andrews 2010) (Accessed on 13 Oct. 2024), and low-quality reads were filtered out using TRIMMOMATIC v. 0.39 (Bolger et al. 2014) based on the quality control results (FASTQC 0.11.9). The high-quality reads were then assembled de novo using SPADES v4.0.0 (Bankevich et al. 2012). Genome assembly quality was assessed using QUAST v. 5.2.0 (Gurevich et al. 2013), and completeness was evaluated with BUSCO v. 5.7.1.1 (Seppey et al. 2019) against the fungi_odb10.2019-11-20 dataset. The genome identities were confirmed by comparing extracted ITS barcode sequences. The genome details are provided in Table 2. The genomes have been deposited in the NCBI database under the bioproject code PRJEB102645.

**Table 2.** Genome sequence data of the type strains of three novel species.

Taxon	Accession number	BUSCO [%]	contigs N50	contigs N90	# N’s	Largest contig	contigs (>= 500 bp)	GC [%]	Total length (genome size, bp)
*Wilhelmdebeerea oxyuri*	ERZ28561635	96.7%	88640	28725	0.00	320914	604	53.8	31505904
*Blastobotrys sasensis*	ERZ28669457	98.2%	412251	168054	0.00	1168760	65	52.1	13044204
*Sugiyamaella casensis*	ERZ28669458	91	352652	117945	0.00	1987081	133	43.8	17419851

### Molecular taxonomy

The ophiostomatoid fungus strain CCF 6802, and the two yeasts CCF 6841 and CCF 6842, could not be assigned to any known species and were subjected to detailed taxonomic analyses. The ITS barcode was obtained as described above. The large-subunit rDNA (LSU) sequence was obtained using the procedure described by Kolařík et al. (2017). TEF1α, the second largest subunit of RNA polymerase II (RPB2), and β-tubulin (TUB2), were extracted from the whole-genome sequence (WGS). Its phylogenetic position was investigated based on a concatenated dataset of four genes (ITS, LSU, TEF1α, and RPB2) and WGS data. A four-gene reference dataset from de Beer et al. (2022), updated to include the recently described genus *Hausneria* (Crous et al. 2024), was used for comparison. BLASTn searches were conducted in the NCBI GenBank database to identify the most closely related sequences for all the genes; however, these searches did not yield any matches closer than those already included in the de Beer et al. (2022) and Crous et al. (2024) datasets. Since only rDNA sequences have been published for some *Intubia* species, we extracted TEF1α and RPB2 sequences from the whole-genome assembly GCA_020002355.1 of *I. oerlemansii*. In accordance with de Beer et al. (2022), *Afroraffaelea ambrosiae* and *Chrysosphaeria jan-nelii* were excluded from the dataset. Our analyses confirmed that these taxa fall far outside the CCF 6802 lineage (*Afroraffaelea* within *Raffaelea* and *Chrysosphaeria* within *Hawksworthiomyces*) and display highly divergent sequences, resulting in long branches and a reduced phylogenetic resolution. *Pyricularia grisea* was used as the outgroup. The final dataset comprised 98 taxa (Suppl. materials 1, 6, 7). Single-gene datasets were separately aligned using MAFFT with the G-INS-i strategy and then concatenated. The original alignment contained 1003 characters from the ITS region, 798 from LSU, 486 from TEF1α, and 1096 from RPB2, totalling 3445 characters. Automatic curation using GBLOCKS v0.91b (Talavera 2007) under less stringent conditions resulted in the loss of 69% of positions. Thus, hyper variable positions were removed manually, and the final dataset had 2830 positions (ITS – 525 bp, LSU – 816 bp, TEF1α – 420 bp, RPB2 - 1 066 bp), comprising 1102 parsimony-informative sites, 266 singleton sites, and 1506 constant sites. Bayesian inference (BI) analyses were conducted using MRBAYES 3.0 (Ronquist and Huelsenbeck 2003), with 10 million generations, and the burn-in was estimated in TRACER v1.5 (Rambaut et al. 2018). The best nucleotide substitution models for each partition were determined using JMODELTEST v2.1.1 (Darriba et al. 2012): ITS = GTR+F+R4, LSU = TNe+I+G4, TEF1α = TIM2+F+I+G4, and RPB2 = GTR+F+I+G4. Maximum likelihood (ML) analyses were performed in IQ-TREE v2.1.3 (Minh et al. 2020), using an automated model selection for each partition via MODELFINDER (Kalyaanamoorthy et al. 2017). Branch support was assessed using both the ultrafast bootstrap approximation and the SH-aLRT test (parameters: -b 1000 -alrt 1000). For the phylogenomic reconstruction, we analysed 78 genomes, representing all the available sequenced species of the family *Ophiostomataceae*, together with four outgroup species (*Cryphonectria parasitica*, *Diaporthe ampelina*, *Phaeoacremonium minimum*, and *Pyricularia grisea*), all retrieved from the NCBI GenBank database (accessed 20 November 2024) (Suppl. material 2). The analysis was conducted using the ARBOPHYL pipeline, available at [https://github.com/WesterdijkInstitute/ArboPhyl]. This pipeline integrates the following steps: (i) identification of conserved genes using BUSCO (Manni et al. 2021), (ii) sequence alignment with MAFFT v7 (Katoh and Standley 2013), (iii) alignment trimming with TRIMAL (Capella-Gutiérrez et al. 2009), and (iv) model selection and phylogenetic tree reconstruction with IQ-TREE v2.1.3. Branch supports were again assessed using the ultrafast bootstrap and SH-aLRT methods (parameters: -b 1000 -alrt 1000). In addition, to obtain more informative measures of branch support, gene and site concordance factors (gCF and sCF) (Lanfear and Hahn 2024) were calculated in IQ-TREE v3.0.0. The gene concordance factors were computed from the set of single-locus trees using the command ‘iqtree3 -t output.nex.treefile --gcf singlelocus_trees_combined.trees --prefix gcf’, and the site concordance factors were calculated from the single-locus alignments using ‘iqtree3 -te output.nex.treefile -p MAFFT_output --scfl 100 --prefix sfc -T 10’. The pipeline was run with the following parameters: mode = genome; BUSCO lineage = *Sordariomycetes*; the percentage of shared BUSCO genes among species = 100%; and the required BUSCO genome completeness = retain all genomes. These settings yielded a final dataset of 522 genes, which were used to infer the phylogenomic tree. The genomic sequence was also inspected for the presence of mating-type idiomorphs. Specifically, homologs of MAT1-1 (alpha domain, JX402993) and MAT1-2 (HMG, JX402994) from *Ophiostoma montium* (Tsui et al. 2013) were searched using the BLASTn tool.

The phylogenetic positions of the yeast strains were evaluated using the ITS–LSU rDNA and WGS datasets. ITS barcodes were obtained as described above, and LSU sequences were extracted from the WGS data. For the ITS–LSU rDNA analyses of the genus *Blastobotrys*, the dataset from the most recent taxonomic revision (Visagie et al. 2024), supplemented with *Blastobotrys guizhouensis* (Hu et al. 2025), and outgroup species *Wickerhamiella domercqiae*, was used (Suppl. materials 3, 8, 9). The original alignment (42 taxa, 7394 bp, MAFFT, G-INS-i) was difficult to align due to numerous introns and high ITS sequence variability. After curation in GBLOCKS under less stringent conditions, 907 bp were retained, while manual editing produced 3262 sites, with 2315 constant and 688 parsimony-informative positions. Phylogenetic analyses of *Sugiyamaella* were based on the dataset of Chai et al. (2022), supplemented with *Sugiyamaella amazoniana* and *Sugiyamaella bielyi* (Souza et al. 2023), using *Tortispora* as the outgroup (Chai et al. 2022) (Suppl. materials 4, 10, 11). This dataset comprised 62 sequences and 4495 sites. As in the case of *Blastobotrys*, the dataset was difficult to align. After curation with GBLOCKS v0.91b under less stringent conditions, only 696 sites, mainly from the 5.8S and LSU regions, were retained. Manual editing produced a final dataset of 1148 nucleotide sites, including 582 constant and 415 parsimony-informative positions.

Phylogenetic analyses were conducted using both Bayesian (MB) and Maximum Likelihood (ML) approaches, as described for the genus *Wilhelmdebeerea*. The ITS and LSU regions were treated as two partitions. The best-fit substitution models were GTR+F+I+R5 (ITS) and GTR+F+I+R3 (LSU) for *Blastobotrys*, and GTR+F+I+R4 (both partitions) for *Sugiyamaella*. For the phylogenomic analysis, 184 reference genomes of species belonging to the *Dipodascomycetes*, together with 10 genomes from related yeast lineages, were retrieved from the NCBI GenBank (accessed on 15 April 2025) (Suppl. material 5). The analysis was conducted using the same ARBOPHYL pipeline and the parameters were as described above for the *Ophiostomatales*, except that the BUSCO lineage was set to *Saccharomycetes*. This analysis yielded a dataset of 1399 conserved single-copy orthologs, which were concatenated for phylogenomic tree construction. *Lipomyces kockii* was used as an outgroup.

### Biogeography based on the GlobalFungi database

The biogeography of the main fungal symbionts was studied using the workflow of Réblová et al. (2022) in the GlobalFungi 5 database (Větrovský et al. 2020). ITS1 and ITS2 rDNA regions were extracted using the ITSX v1.1.3 extractor (Bengtsson-Palme et al. 2013) implemented in the SEED2 v2.1.3. software (Větrovský et al. 2018). We subjected all unique ITS1 and ITS2 haplotypes to an exact-match identity search in GlobalFungi, which searches for sequences identical in both length and sequence. In addition, we also used a BLASTn group search to find the most similar sequences and used a sequence identity of 100–99.5% as the threshold for assignment to individual species.

## Results

### Community composition

We studied fungi collected from the surface of larvae, the female thorax (including mycangium; hereafter referred to as mycangium), and the active part of the larval gallery where larvae were present. These galleries were cream to light brown in colour. Across all studied substrates, we identified five dominant symbionts, which we quantified using the dilution method and characterised by morphology and ITS barcoding (Fig. 1F). *Candida schatavii* dominated in the larvae samples, was the second most frequent symbiont in the mycangia, and was less common in the galleries. Another representative of *Saccharomycotina*, *Magnusiomyces fungicola*, dominated in the galleries and was less frequent on the larvae or in the mycangia. A member of *Ophiostomatales*, represented by the strain CCF 6802 and described in this paper as *Wilhelmdebeerea oxyuri*, dominated in the mycangia and was also common in the galleries. The other fungi, *Ophiostoma pityokteinis*, *Blastobotrys* sp. CCF 6841 (described below as *B. sasensis*) and *Sugiyamaella* sp. CCF 6842 (described below as *Sugiyamaella casensis*) represented a minor component of the fungal community (Table 1). The GenBank accession numbers for the representative strains are PX523829, PX523830 (*C. schatavii*CCF 6799 = CCY 26-26-30, CCF 6800 = CCY 26-26-26), PX523831 (*M. fungicola*CCF 6801 = CCY 99-2-1), PX523828 (*B. sasensis*CCF 6841 = CCY 100-1-1), PX523827 (*S. casensis*CCF 6842 = CCY 101-1-1), PX523832, PX523833 (*W. oxyuri*CCF 6802, CCF 6803) and PX523834 (*O. pityokteinis* TOX-8).

### Phylogeny

**Figure 2.**
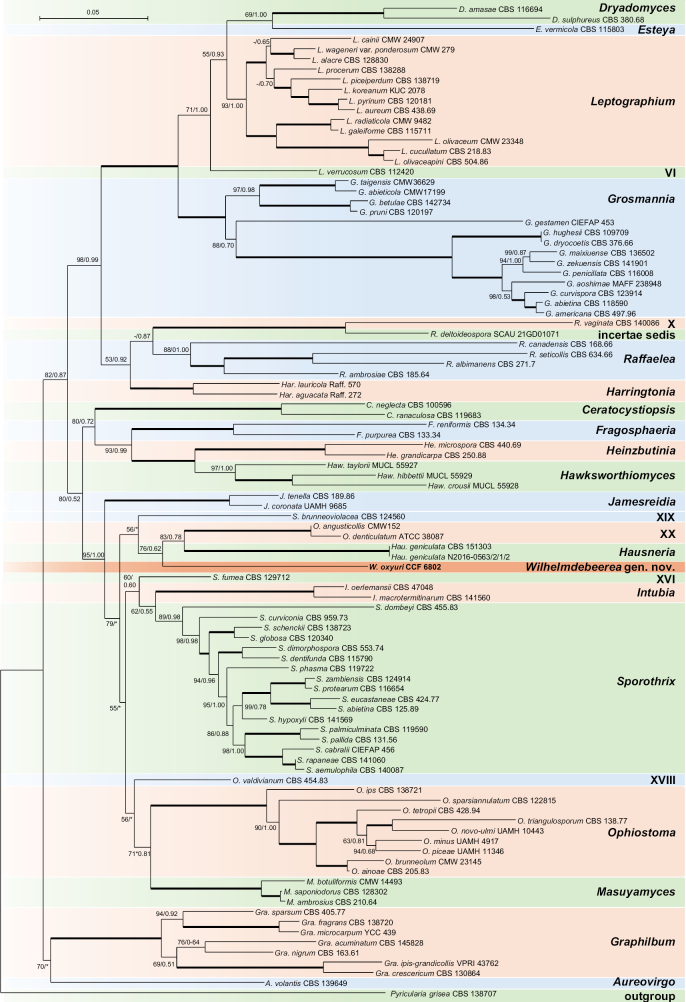
Phylogenetic position of *Wilhelmdebeerea oxyuri* was resolved using a four-gene dataset (ITS, LSU, TEF1α, RPB2). The tree was obtained using the Maximum-likelihood method in IQ-TREE2. Node support is provided as Maximum-likelihood bootstrap/Bayesian posterior probability; values ≥ 50/0.5 are shown. An asterisk denotes disagreement in the topology between two phylogenetic methods. Branches marked with “+” were shortened twice. Branches with full statistical support (100/1.00) are shown in bold. Numbering of the undescribed genera follows de Beer et al. (2022). *Pyricularia grisea* was used as the outgroup.

**Figure 3.**
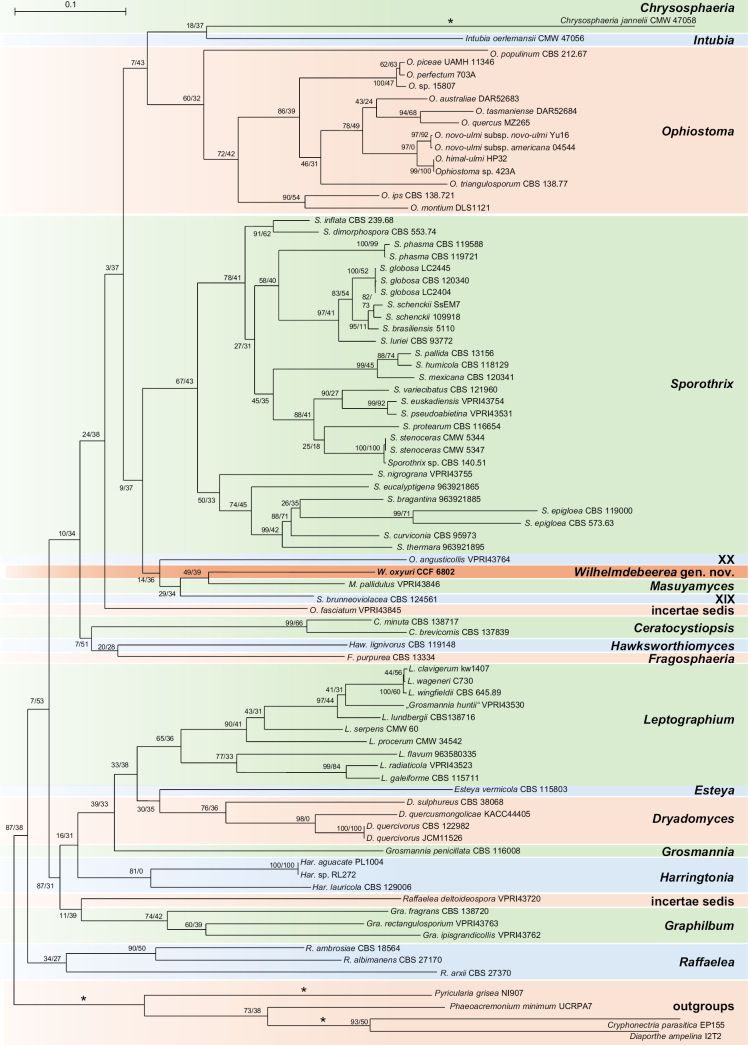
Phylogenetic position of *Wilhelmdebeerea oxyuri* was resolved using 522 BUSCO genes. The tree was obtained using the Maximum-likelihood method in IQ-TREE2. Node support is reported as GFC/SCF. Branches marked with an asterisk were shortened twice. *Cryphonectria parasitica*, *Diaporthe ampelina*, *Magnaporthe grisea*, *Magnaporthe poae* and *Phaeoacremonium minimum* were included as outgroup taxa.

We focused on the phylogenetic position of unidentified fungi represented by strains CCF 6802 (*Ophiostomatales*), CCF 6841, and CCF 6842 (*Dipodascales*), described below as a new species. For CCF 6802, the best hits in the NCBI GenBank and UNITE databases were ≤ 83% for ITS, ≤ 97% for LSU, 96% for TEF1α, 85% for RPB2, and 76% for TUB2, matching various species of *Harringtonia*, *Hawksworthiomyces*, *Masuyamyces*, *Ophiostoma*, *Raffaelea*, and *Sporothrix*. Phylogenetically, this strain formed a separate lineage related to several taxa, depending on the dataset and analytical method used. In the multilocus ML analysis, it clustered with the clade containing *Hausneria geniculata*, *Ophiostoma angusticollis* and *O. denticulatum*, whereas in the MB analysis, its sister clade was the genus *Intubia*. Additional related taxa included *Sporothrix brunneoviolacea*, *Sporothrix fumea*, and *Ophiostoma valdivianum* (Fig. 2). Phylogenomic analyses resolved *Masuhomyces pallidulus* as the sister taxon (Fig. 3).

**Figure 4.**
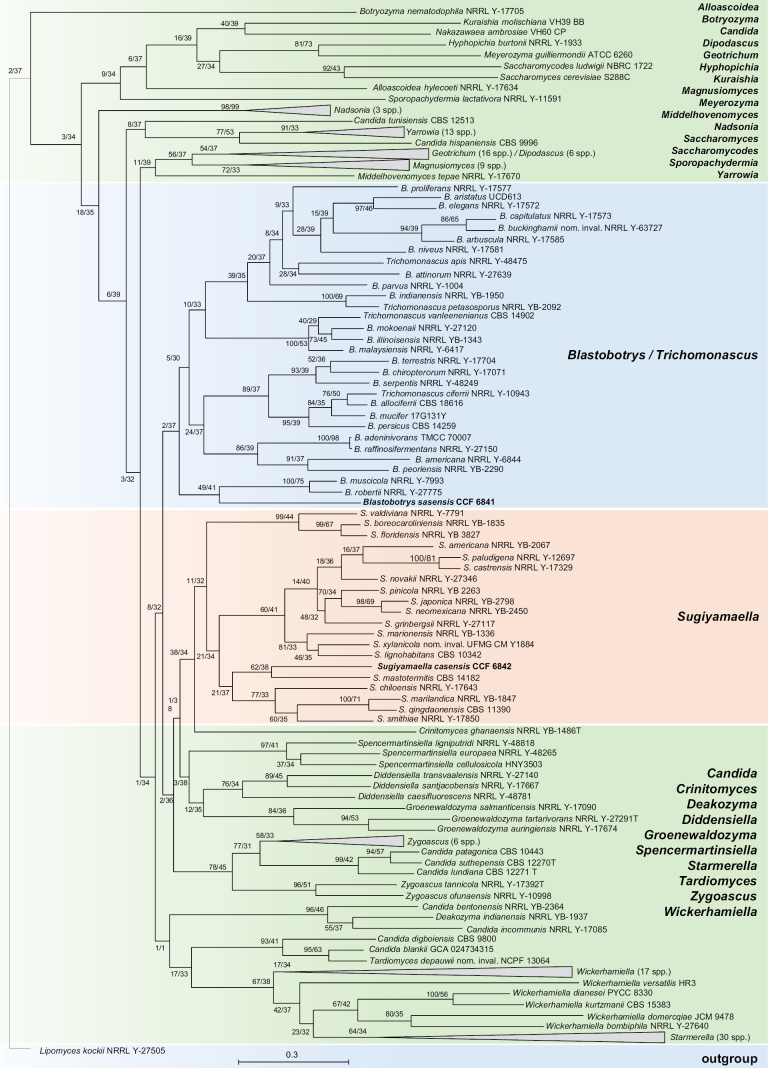
Phylogenetic position of *Blastobotrys sasensis* and *Sugiyamaella casensis* was resolved using 1399 BUSCO genes and 195 genomes representing *Dipodascomycetes* and related lineages. The tree was obtained using the Maximum-likelihood method in IQ-TREE2. Node support is reported as GFC/SCF. *Lipomyces kockii* was included as the outgroup.

For *Blastobotrys sasensis*CCF 6841, the best ITS hit was *Blastobotrys adeninivorans* CBS 8244 (KY101746), with 85% identity and 60% coverage. For LSU (500 bp), the best hit was *Blastobotrys muscicola* CBS 10338 (KY106208), with 96.1% identity and 81% coverage. For *Sugiyamaella casensis*CCF 6842, the best ITS hit was *Sugiyamaella marionensis* NRRL YB-1336 (NR_111237), with 86.7% identity and 80% coverage, while the LSU (500 bp) best hit was *Sugiyamaella mastotermitis* CBS 14182 (NG_058230) with 95.58% identity and 89% coverage. In both yeasts, a low ITS coverage was observed because the ITS2 region of CCF 6841 and the ITS1 region of CCF 6842 were highly divergent, preventing reliable alignment. Based on phylogenetic analyses, both species were placed in the family *Trichomonascaceae*, within the order *Dipodascales* (Fig. 4).

**Figure 5.**
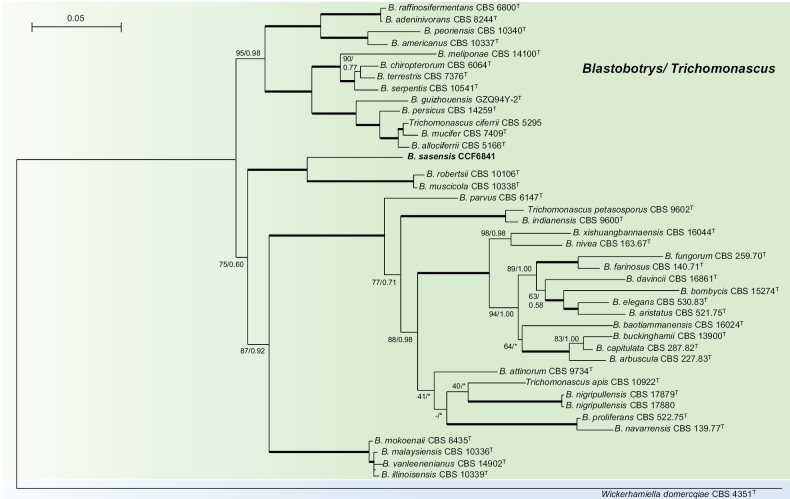
Phylogenetic position of *Blastobotrys sasensis* was resolved using the ITS-LSU rDNA dataset. The tree was obtained using the Maximum likelihood method in IQ-TREE2. The statistical support for nodes is given as Maximum likelihood bootstrap/Bayesian posterior probability; values ≥ 50/0.5 are shown. The branch marked with “+” was shortened twice. An asterisk denotes disagreement in the topology between two phylogenetic methods. The branches with full statistical support (100/1.00) are shown in bold. *Wickerhamiella domercqiae* was used as the outgroup.

**Figure 6.**
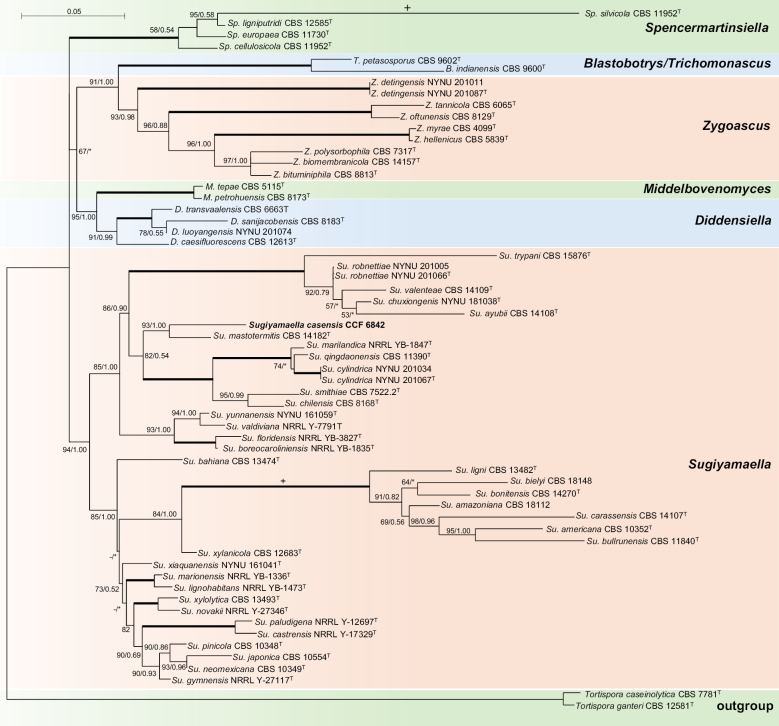
Phylogenetic position of *Sugiyamaella casensis* was resolved using the ITS-LSU rDNA dataset. The tree was obtained using the Maximum-likelihood method in IQ-TREE2, and node support is provided as maximum-likelihood bootstrap/Bayesian posterior probability. The values ≥ 50/0.5 are shown. The branch marked with “+” was shortened twice. An asterisk denotes disagreement in the topology between two phylogenetic methods. The branches with full statistical support (100/1.00) are shown in bold. *Tortispora* spp. were used as the outgroup.

The phylogenomic tree, based on 1,399 genes, placed *B. sasensis* within the monophyletic genus *Blastobotrys*, as a lineage sister to *B. muscicola* and *B. robertii*. *Sugiyamaella casensis* was placed in the monophyletic genus *Sugiyamaella*, as a sister species to *S. mastotermitis*. MB and ML phylogenetic analyses of the concatenated ITS-LSU dataset confirmed this placement. The sister lineages were again *B. muscicola* and *B. robertii* for *B. sasensis* and *S. mastotermitis* for *S. casensis* (Figs 5, 6). Across all methods, *Sugiyamaella* was consistently recovered as a monophyletic genus.

### Biogeography

Based on the GlobalFungi database, ITS sequences identical to those of *W. oxyuri* were found in conifer deadwood samples from forests in Europe. Specifically, in 27 *Picea abies* samples collected in the Helsinki area, Finland (Korhonen et al. 2022), and in two samples from conifer trees (*Picea abies* or *Abies sibirica*) in the Sverdlovsk Oblast, European Russia (Mikryukov et al. 2021). When the sequence identity threshold was relaxed to 99.5–100%, the number of samples from the Finnish study increased to 42.

*Candida schatavii* was detected in five samples collected from deadwood in a European forest biome. Specifically, in four samples from *Picea abies* in the Helsinki area (Korhonen et al. 2022) and one from an undetermined tree in the Czech Republic (Baldrian et al. 2016).

An exact-hit search for *Blastobotrys sasensis* returned 54 samples, all from deadwood collected in forest biomes in Europe and Asia. Specifically, in 53 samples of *Picea abies* from the Helsinki area (Korhonen et al. 2022) and one sample from an undetermined tree in the Asian part of Russia (Mikryukov et al. 2021).

The GlobalFungi database does not include records of *Magnusiomyces fungicola* and *Sugiyamaella casensis* (best hits ≤ 93%). Interestingly, detections of *C. schatavii*, *B. sasensis*, and *W. oxyuri* from Finland originated from the same locations, the same study, and in some cases, the same samples (Suppl. material 12).

### Taxonomy

#### Wilhelmdebeerea

Taxon classificationAnimalia

M. Kolařík gen. nov.

64628AFC-8FA1-5D7E-A44C-68C81570884D

861308

##### Etymology.

In honour of Wilhelm de Beer, for his contributions to mycology.

##### Type species.

*Wilhelmdebeerea oxyuri* M. Kolařík.

##### Diagnosis.

Known from the asexual state only. It produces both hyalorhinocladiella-like and leptographium-like asexual states. Such a combination of characteristics is rare in *Ophiostomatales* and is known only in several phylogenetically unrelated species.

#### Wilhelmdebeerea oxyuri

Taxon classificationAnimalia

M. Kolařík sp. nov.

B85B9A0A-AACF-51B6-8AC8-07748CED13D0

861309

Fig. 7

##### Etymology.

The epithet *oxyuri* refers to the specific beetle associated with this fungus.

##### Diagnosis.

*Wilhelmdebeerea* is a monotypic genus and the species and genus diagnoses are identical until additional species are found.

##### Type.

SLOVAKIA • Zvolen region, Tŕnie, 48.627397°N, 19.029675°E; alt. 665 m.; from larva of *Treptoplatypus oxyurus* feeding in the base (40 cm diam) of a decaying *Abies alba*; 10. June 2024; leg. M. Knížek, J. Vakula, M. Zúbrik, isol. M. Kolařík Tox-2 (***holotype*** PRM 963297 dried culture CCF 6802 on MEA, ***isotypes*** PRM 963298, 963299, dried culture CCF 6802 on MEA with cycloheximide, culture ex type CCF 6802). Another representative strain – CCF 6803, the same source as CCF 6802, but isolated from mycangia of a female adult. Sequence accessions: ITS - PX523832, LSU - PV061845, , rpb2 - PV067587, tef1-α - PV067588, tub2 - PV067589, WGS - ERZ28561635.

**Figure 7.**
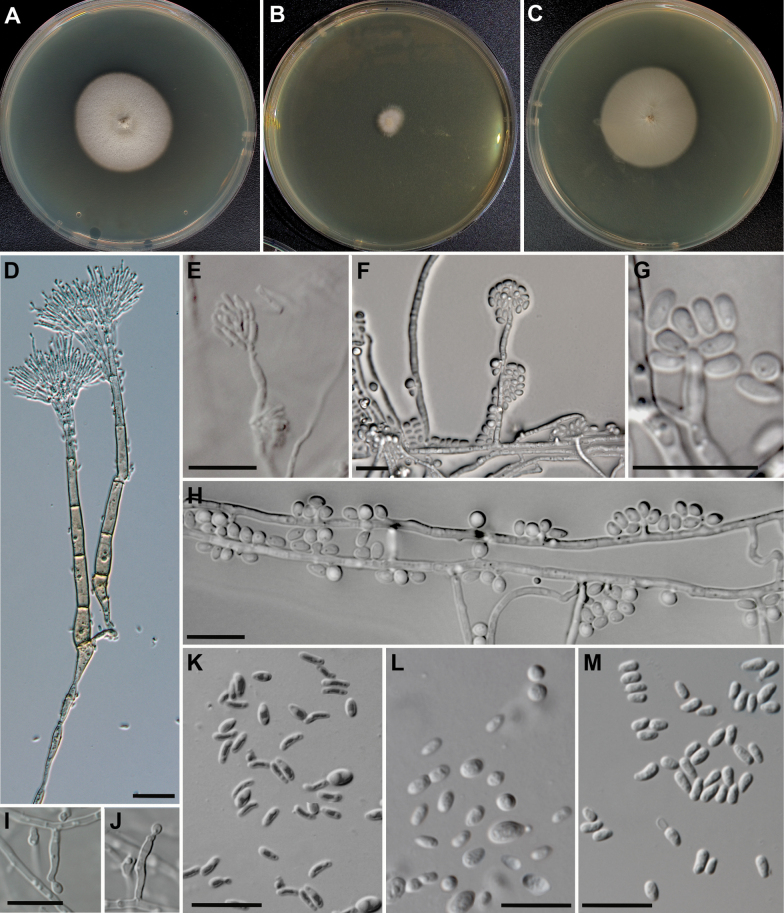
Morphology of *Wilhelmdebeerea oxyuri*. **A**. MEA, 14 d, 25 °C. **B**. MEA, 14 d. 5 °C. **C**. MEA with 500 mg/L cycloheximide, 25 °C. 14 d. **D**. Leptographium-like anamorph. **E**. Hyalorhinocladiella-like anamorph, taken from the MEA agar plate. **F–H**. Hyalorhinocladiella-like anamorph taken from slide culture. **K**. Conidia of hyalorhinocladiella-like anamorph, taken from the MEA agar plate. **L**. Hyalorhinocladiella-like anamorph taken from slide culture. **M**. Conidia of leptographium-like anamorph. Scale bars: 20 µm (**D**); 10 µm (**E–M**).

##### Description.

The species description is based on the type strain. The strain CCF 6803 has the same morphology. Cultural characteristics after 14 d: Colonies with optimal growth at 25 °C (33 mm, 14 d). Growth at 5 °C 8.5 mm, no growth at 35 °C. Colonies at 25 °C are cream with light brown zones, and the margin is narrow. Hyphae are hyaline to olive brown in colour, smooth, submerged in the medium; aerial mycelium is sparse, cream coloured. The strain grows well (32 mm in 14 d) on MEA with 500 mg/L of cycloheximide. ***Sexual morph*** is not observed. ***Asexual morph*** on MEA is mononematous, of the hyalorhinocladiella-like and leptographium-like type. ***Hyalorhinocladiella*-*like conidiophores***, arising directly from submerged or aerial mycelium, are smooth, hyaline, micronematous or semimicronematous, 5.0–45.0 × 1.0–3.0 µm; ***conidia*** are variable in shape, hyaline, cylindrical, allantoid to broadly ellipsoidal, or clavate (1.5–) 2.7–3.9 ± 0.66 (–4.8) × (0.8–) 1.0–2.0 ± 0.30 (–3.0) µm. ***Leptographium*-*like anamorph*. *Conidiophores*** are macronematous, light olivaceous or olivaceous, smooth, arising from substrate hyphae, solitary or loosely compacted, resembling cream sporodochia, (100–) 120–230 (–450) μm in length. Stipe erect, olivaceous, 3–5 septate, (60–) 80.0–180.0 (–280.0) μm long and (3.0–) 4.8–5.2 (–6.2) μm wide at the base; basal cell is often swollen with a foot-like cell. Conidiogenous apparatus is (50.0–) 55.0–65.0 (–200.0) μm long (excluding conidial mass), consisting of 3–5 (mostly 3) series of branches-type B (more than two branches) (Jacobs and Wingfield 2001). Primary branches are (14.0–)15.5–+18.5(–24.0) × (4.0–) 4.4 (–5.0) μm in size. ***Conidiogenous cells*** are hyaline, tapering from base to apex, (6.0–) 9.0 (–15.0) × (0.7–)1.2(–1.3) μm in size. ***Conidia*** are hyaline, clavate (2.6–) 3.3–5.9 ± 0.68 (–7.5) × (1.1–) 1.5–2.3 ± 0.25 (–2.9) μm in size, accumulating around the conidiogenous apparatus as a creamy mucilaginous mass. ***Chlamydospores*** are absent.

##### Distribution.

*Wilhelmdebeerea oxyuri* was cultured from a single locality in Slovakia. Further investigations are required to determine the strength of its association with *T. oxyurus*. Based on GlobalFungi data, the species has been detected in 45 deadwood substrate samples from boreal forest ecosystems in Finland and Russia (Mikryukov et al. 2021; Korhonen et al. 2022) (see Biogeography section, Suppl. material 12). The host records suggest that *W. oxyuri* may be common, but poorly sampled, in coniferous wood in boreal Eurasia, not only in *Abies*, the exclusive host of *T. oxyurus*. Its distribution overlaps with *B. sasensis* and *C. schatavii*.

##### Notes.

The genomic sequence of monosporic ex-type strains contains both MAT gene idiomorphs, and the species can be considered homothallic. In particular, it possesses the MAT1-1 gene sequence, which has an 83% identity, and the MAT1-2 gene, which has an 84% identity, to the reference sequences of *Ophiostoma montium*.

#### Blastobotrys sasensis

Taxon classificationAnimaliaSaccharomycetalesTrichomonascaceae

M. Kolařík & R. Vadkertiová sp. nov.

669D9355-CC79-5394-BBFC-366804C49C57

861310

Fig. 8

##### Etymology.

The epithet sasensis is derived from the acronym SAS (Slovak Academy of Sciences) combined with the Latin suffix -*ensis* (i.e., originating from). The name honours the institution’s role in advancing scientific research in Slovakia.

##### Diagnosis.

*Blastobotrys sasensis* differs from the closely related species *B. muscicola* by its ability to utilise d-xylose, erythritol, sorbose, glucitol, and ribitol. Another related species, *B. robertii*, differs by its ability to assimilate glycerol (Suppl. material 13: table SS1). It also differs from both closely related species in its origin. *B. muscicola* was isolated from a moss growing on a fallen log in USA (Kurtzman 2007) and *B. robertii* was isolated from rotten pine wood (*Pinus sylvestris*) in the Netherlands (Middelhoven and Kurtzman 2007).

**Figure 8.**
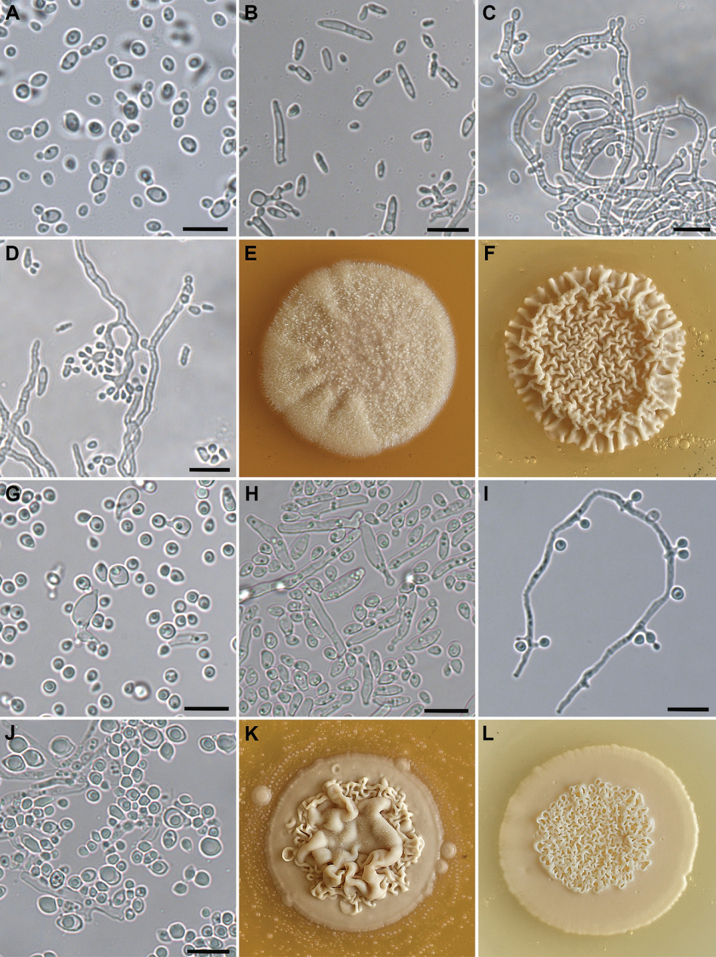
Morphology of two new yeast species. *Blastobotrys sasensis*. **A**. Budding cells, 24 h., 28 °C. **B**. Spindle-shaped cells YM, 5 d 28 °C. **C**. Mycelia with blastoconidia, MEA 7 d, 28 °C. **D**. Multiple budding of mother cell, MEA 8 d, 28 °C. **E**. Colony morphology on YMA cultivated for 21 d, 22 °C (diameter is 22 mm). **F**. colony morphology on MEA cultivated for 21 days (diameter is 24 mm). *Sugiyamaella casensis*. **G**. budding cells, 7 d., ME medium, 28 °C. **H**. short chains of mycelia with blastoconidia, YM medium, 7 d 28 °C. **I**. mycelium with blastoconidia, MEA 8 d, 28 °C. **J**. yeast cells with different shapes and sizes of lipid particles, MEA 21 d, 28 °C. **K**. colony morphology on MEA cultivated for 21 d, 22 °C (diameter is 30 mm). The surface of the MEA is dotted with blastoconidia that are ejected upon maturity. **L**. colony morphology on YMA cultivated for 21 days (diameter is 27 mm). Scale bars: 10 µm.

##### Type.

SLOVAKIA • Zvolen region, Tŕnie, 48.627397°N, 19.029675°E; alt. 665 m.; from thorax of *Treptoplatypus oxyurus* feeding in the base (40 cm diam) of a decaying *Abies alba*; 10. June 2024; leg. M. Knížek, J. Vakula, M. Zúbrik, isol. M. Kolařík Tox-13 (***holotype***CCY 100-1-1, culture preserved in a metabolically inactive state in the Culture Collection of Yeasts, Bratislava, Slovakia, ex-type culture CCF 6841). Sequence Accessions: ITS – PX523827, LSU - PX591253, WGS - ERZ28669457.

##### Description.

On YMA, after 7 days of incubation at 28 °C, the culture is slightly beige, dry and wrinkled. On MEA under the same incubation conditions, the culture is white, dry and powdery. On YMA, after 21 days of cultivation, the colony is raised, crateriform, cerebriform, with an undulate margin and a cream-coloured centre. On MEA, after 21 days of cultivation, the colony is raised, filamentous, and fluffy, with an undulate margin and a cream-coloured centre (Fig. 8E, F). In both ME medium and YM, after 7 days of incubation at 28 °C, the ***cells*** are oval to elongate, often spindle-shaped, 1.5–3 × 2.5–17 µm (Fig. 8A, B). A sediment and a thick pellicle are formed in both media after 7 days of incubation at 28 °C. ***Conidiophores*** with primary and secondary conidia are formed on MEA after 7 days, abundant hyphae are present. Multiple budding of mother cells occurs (Fig. 8C, D). No sexual reproduction is observed.

Physiological and biochemical characteristics. Fermentation of glucose and d-xylose is absent. The following carbon compounds are assimilated: glucose, fructose, mannose, galactose, maltose, saccharose, lactose, raffinose, d-xylose, cellobiose, trehalose, soluble starch, melibiose, l-sorbose, salicin, ribitol, d-mannitol, d-glucitol, erythritol, glycerol, n-acetyl-d-glucosamine (w), succinic acid, and citrate. The other compounds- melezitose, l-arabinose, inulin, l-rhamnose, d-arabinose, d-ribose, xylitol, *myo*-inositol, ethanol, methanol, glycerol and dl-lactate - are not assimilated. The nitrogen compounds - ethylamine, l-lysine, cadaverine and n-acetyl-d-glucosamine are assimilated, whereas nitrate, nitrite, and creatinine are not assimilated. The urease reaction is negative. Starch-like polysaccharides are not produced. Growth in a vitamin-free medium (w), 10% NaCl+ 5% glucose and 50% glucose is positive. Growth at a temperature range from 5 °C to 30 °C is positive, growth at 35 °C is negative.

##### Distribution.

The species is currently known only from a culture at the type locality in Slovakia. The NCBI GenBank contains the LSU sequence KF617761, which has a 99.4% identity and may represent our species. It is a sequence of an uncultured fungus obtained from *Picea mariana* forest soil in Alaska by Taylor et al. (2014). Based on the GlobalFungi exact hit, it was found in 54 samples, all originating from deadwood of *Picea abies* (53 samples) or an undetermined tree (one sample) collected in Finland (53 samples) by Ovaskainen (2010) and Korhonen (2022) and in the Asian part of Russia (Mikryukov et al. 2021). This suggests that it is associated with conifers in temperate and boreal Eurasia, not necessarily with *T. oxyurus*, which is specific to *Abies alba*.

##### Notes.

Based on the phylogenomic and ITS-LSU rDNA analyses, it forms a lineage sister to *B. muscicola* and *B. robertii* (Figs 4, 5).

#### Sugiyamaella casensis

Taxon classificationAnimaliaSaccharomycetalesTrichomonascaceae

M. Kolařík & R. Vadkertiová sp. nov.

7C291BBF-AB6A-5C34-B321-2612CB9A89BE

861311

Fig. 8

##### Etymology.

The epithet casensis is derived from the acronym CAS (Czech Academy of Sciences) combined with the Latin suffix -ensis (i.e., originating from). The name honours the institution’s role in advancing scientific research in the Czech Republic.

##### Diagnosis.

*Sugiyamaella casensis* differs from the closely related species *Sugiyamaella mastotermitis* in its inability to ferment saccharides and to grow at 40 °C. Moreover, unlike *S. mastotermitis*, it assimilates d-ribose, ribitol and glycerol (Suppl. material 13). The strain of *S. mastotermitis* was isolated from the gut contents of the termite *Mastotermes darwiniensis* (laboratory culture) in Germany (Handel et al. 2016), whereas *S. casensis* was isolated from the larvae of *Treptoplatypus oxyurus* in Slovakia.

##### Type.

SLOVAKIA • Zvolen region, Tŕnie, 48.627397°N, 19.029675°E; alt. 665 m.; from thorax of *Treptoplatypus oxyurus* feeding in the base (40 cm diam) of a decaying *Abies alba*; 10. June 2024; leg. M. Knížek, J. Vakula, M. Zúbrik, isol. M. Kolařík Tox-17 (***holotype***CCY 101-1-1, culture preserved in a metabolically inactive state in the Culture Collection of Yeasts, Bratislava, Slovakia, ex-type culture CCF 6842). Sequence accessions: ITS – PX523828, LSU - PX591254, WGS - ERZ28669458.

##### Description.

On YMA, after 7 days of incubation at 28 °C, the culture is white, soft and slightly wrinkled. On MEA, under the same incubation conditions, the culture is white, dry and powdery. On YMA, after 21 days of cultivation, the colony is soft, with a flat undulate margin and a raised, rugose centre, cream in colour. On MEA, after 21 days of cultivation, the colony is cream-coloured, raised, with a flat, undulate margin and an elevated centre, slightly wrinkled, with a filamentous growth in the upper layer (Fig. 8K). In both ME and YM media, after 7 days of incubation at 28 °C, the ***cells*** are globose, oval to ovoidal (1.1–4.2 × 2.7–12 µm), some of them with a blunt end, and occur singly or in pairs. Short hyphae with ***blastoconidia*** occur (Figs 8G, 8H). Sediment and slight cloudiness are formed in both media. True hyphae with blastoconidia, arising from denticles, are formed on MEA after 7 days at 28 °C (Fig. 6I). Lipid particles of various shapes and sizes are formed after 21 days at 25 °C (Fig. 6J). No sexual reproduction is observed.

Physiological and biochemical characteristics. Fermentation of glucose and d-xylose is absent. The following carbon compounds are assimilated: glucose, fructose, mannose, galactose, maltose, saccharose, raffinose, melezitose, d -xylose, l -arabinose, cellobiose, trehalose, soluble starch, melibiose, l-rhamnose, d -sorbose, d -ribose, salicin, xylitol, ribitol, d -mannitol, d -glucitol, erythritol, *myo*-inositol, ethanol, glycerol, n-acetyl- d -glucosamine, succinic acid, and citrate. The other carbon compounds - lactose, inulin, d -arabinose, methanol and dl-lactate- are not assimilated. The nitrogen compounds: ethylamine and n-acetyl- d -glucosamine are assimilated, whereas nitrate, nitrite, l-lysine, cadaverine, and creatinine are not assimilated. The urease reaction is negative. Starch-like polysaccharides are not produced. Growth in a vitamin-free medium, 10% NaCl+ 5% glucose (w) and 50% glucose is positive. Growth at 5 and 10 °C is weak. Growth at a range from 20 °C to 30 °C is positive. Growth at 35 °C is negative.

##### Distribution.

It is currently documented solely from the type locality in Slovakia. Further investigations are required to determine whether its distribution corresponds to the range of its insect vector, *T. oxyurus*. No identical or similar (≥ 90%) sequences were found in the GlobalFungi database.

##### Notes.

Based on the phylogenomic and ITS-LSU rDNA analyses, it forms a lineage sister to *S. mastotermitis* (Fig. 4, 6).

## Discussion

In our study, we identified three fungi numerically dominant in the thorax, which contains mycangia, as well as in the microhabitat of *Treptoplatypus oxyurus* galleries in fir (*Abies alba*). This includes the new species and genus *Wilhelmdebeerea oxyuri*, within the *Ophiostomatales*, and two yeast species, *Magnusiomyces fungicola* and *Candida schatavii*. Two additional yeast species, isolated at much lower frequencies, are described here as new species of the genera *Sugiyamaella* and *Blastobotrys*.

*Wilhelmdebeerea* is distinguished by the concurrent production of leptographium-like and hyalorhinocladiella-like asexual morphs. Within *Ophiostomatales*, leptographium-like morphs are typical of *Grosmannia* and *Leptographium*, are rare in *Ophiostoma*, and are absent in other genera. The co-occurrence of leptographium-like and hyalorhinocladiella-like morphs, or morphologically similar sporothrix-like morphs, is uncommon, having been observed only in some species of *Grosmannia* and *Leptographium*, and in *O. valdivianum* (de Beer et al. 2016; de Beer et al. 2022).

The phylogenetic position of *W. oxyuri* remains unresolved, as different genetic datasets and analytical methods yield conflicting placements. Consistently recovered relatives include *Hausneria geniculata*, which is associated with *Alnus*-feeding bark beetles in Norway and possesses a pesotum-like and hyalorhinocladiella-like/sporothrix-like anamorph (Crous et al. 2024). Other consistently related taxa were *O. angusticollis* and *O. denticulatum*, which are associated with Northern Hemisphere bark beetles and possess sporothrix-like anamorphs (Villarreal et al. 2005; de Beer et al. 2016). The next related genus is *Intubia*, characterized by a hyalorhinocladiella-like anamorph and by its occurrence in abandoned termite combs in Africa (Nel et al., 2021). Additional related taxa include *S. brunneoviolacea* (Europe; soil, roots) and *S. fumea* (South Africa; *Eucalyptus*, *Phoracantha* beetle galleries), both of which produce sporothrix-like anamorphs (Madrid et al. 2010; Kamgan Nkuekam et al. 2012). Finally, *O. valdivianum*, associated with *Nothofagus* in Chile, exhibits both sporothrix-like and leptographium-like morphs (Butin and Aquilar 1984). Phylogenomic analyses, while limited in taxon sampling, resolve *M. pallidulus* as a sister taxon. The genus *Masuyamyces* is characterised by a hyalorhinocladiella-like anamorph, sometimes occurring alongside a sympodial pesotum-like anamorph (de Beer et al. 2022).

Our phylogenomic analysis represents the most comprehensive sampling of *Ophiostomatales* taxa to date. Except for taxa not included in de Beer et al. (2022), our results confirm the genus-level classification proposed by these authors. Furthermore, our analysis corroborates the distinct phylogenetic positions of *R. deltoideospora* and *Masuyamyces*, a finding initially suggested by the four-gene analyses in our study and in that of de Beer et al. (2022).

Two yeast species isolated with low frequencies were found to represent new members of the family *Trichomonascaceae* within the order *Dipodascales*. *Blastobotrys* (syn. *Trichomonascus*) is a yeast genus currently comprising 39 accepted species (Visagie et al. 2023; Visagie et al. 2024; Hu et al. 2025). Its members have been isolated from diverse substrates, including soil, cave sediment, plant surfaces, and animal guts, but the ecological specialisation of many species remains unclear (Kurtzman and Robnett 2007). Among insect-associated species, *Blastobotrys baotianmanensis* was isolated from the gut of the ground beetle *Pterostichus gebleri* (Chai et al. 2020). *Blastobotrys meliponae* was isolated from stingless bees of the genus *Melipona* (Crous et al. 2016). Together, these findings suggest that the insect gut may be a significant ecological niche for members of this genus. *Blastobotrys* species are generally osmotolerant, xerotolerant, and thermotolerant, and some have known biotechnological uses (Visagie et al. 2023). Our results confirm the osmotolerance of *B. sasensis*, as it exhibited positive growth in the presence of 50% glucose and on a medium with 10% NaCl + 5% glucose. However, it was not thermotolerant, as it did not grow above 30 °C.

The genus *Sugiyamaella* currently comprises 34 described species (Kurtzman 2007; Kurtzman and Robnett 2007; Morais et al. 2013; Handel et al. 2016; Sena et al. 2017; Huang et al. 2018; Crous et al. 2019; Shi et al. 2021; Chai et al. 2022). Members of *Sugiyamaella* have been isolated from wood-ingesting insects and insect frass, as well as from rotting wood, forest soil, mushrooms, and peat (Kurtzman 2007; Wang et al. 2010; Houseknecht et al. 2011; Kurtzman 2011; Morais et al. 2013; Handel et al. 2016; Tanahashi and Hawes 2016; Sena et al. 2017; Huang et al. 2018; Kachalkin et al. 2024). Notably, *Sugiyamaella chiloensis* was previously detected in individuals of *T. oxyurus* in Croatia (Meštrović 2023).

Both new yeast species utilised a wide range of carbon sources. Of the 34 carbon compounds tested, *B. sasensis* utilised 23 and *S. casensis* utilised 29; however, neither species fermented saccharides. The bark and wood of trees contain various types of saccharides in different amounts. The bark of *Abies alba* was found to contain monosaccharides such as glucose (9.00 g/kg dry bark), galactose (1.06 g/kg), fructose (9.76 g/kg), mannose (<0.01 g/kg), and arabinose (<0.01 g/kg), as well as the disaccharide saccharose (0.38 g/kg) and the trisaccharide raffinose (0.07 g/kg) (Bianchi et al. 2015). Di Lella et al. (2019) compared the carbohydrate composition of wood in living *Abies alba* trees with that of coarse woody debris at various stages of decomposition. The authors detected six monosaccharides (glucose, fructose, galactose, arabinose, mannose, and xylose), four disaccharides (sucrose, maltose, trehalose, and cellobiose), and two sugar alcohols (myo-inositol and sorbitol) in different concentrations. Among these, glucose (0.19–3.04 g/kg), sucrose (0.21–1.96 g/kg), fructose (0.13–0.58 g/kg), and trehalose (0.01–0.39 g/kg) were the most abundant. The concentrations of mannose, arabinose, and xylose did not exceed 0.005 g/kg in living wood. However, during decomposition, the concentrations of mannose, xylose, and arabinose increased significantly (up to 0.22 g/kg, 0.41 g/kg, and 0.76 g/kg, respectively). Our results demonstrate that both new species can assimilate nearly all the saccharides detected in the bark and wood by Bianchi et al. (2015) and Di Lella et al. (2019). The sole exceptions were l-arabinose and the sugar alcohol myo-inositol, which were assimilated only by *S. casensis*. This indicates strong adaptations for life in deadwood.

Some studies have questioned whether the insect-inhabiting species of the genera *Blastobotrys* and *Sugiyamaella* are more closely related to wood or to wood-ingesting insects, in which they participate in the digestion of various compounds (Kurtzman 2007; Houseknecht et al. 2011; Shi et al. 2021). Although the assimilation spectrum of the described new yeast species corresponded to the composition of the bark and wood (live and decomposing) and although they were isolated with very little frequency, we can only speculate that the species are more likely associated with wood than with wood-ingesting insects.

Many *Sugiyamaella* species are potential xylanase producers, and some can ferment d-xylose, highlighting their economic potential for producing bioethanol and xylitol from plant waste (Morais et al. 2013; Lara et al. 2014; Handel et al. 2016; Sena et al. 2017; Shi et al. 2021). Consequently, *Sugiyamaella* species are relevant not only as wood-decomposing organisms but also as promising candidates for applications in food, medicine, and biofuel production. Both yeast strains described assimilate xylose, and their biotechnological potential remains to be verified.

Phylogenetic analyses based on ITS and LSU sequences have previously suggested that *Sugiyamaella* is polyphyletic or paraphyletic. Specifically, ITS–LSU phylogenies have revealed two major clades: one clustering around *S. smithiae* (the type species) and the other around *S. americana*, with several *Sugiyamaella* species intermixed with taxa from the genera *Diddensiella*, *Middenhovenomyces*, and *Spencermartinsiella* (Sena et al. 2017; Shi et al. 2021; Chai et al. 2022). However, our phylogenomic analyses based on whole-genome sequencing (WGS) and a concatenated ITS–LSU dataset do not support this view. In our results, all *Sugiyamaella* species were monophyletic, with *Crinitomyces*, *Diddensiella*, *Middenhovenomyces*, and *Spencermartinsiella* appearing as well-supported yet independent sister lineages (Fig. 4, 6). The discrepancies among previous studies may be attributable to the high variability of the ITS region, which can lead to homoplasy. Indeed, when we analysed uncurated ITS–LSU datasets, we failed to recover strong statistical support for the monophyly of *Sugiyamaella* (data not shown). Such analytical artifacts can be mitigated by excluding hypervariable regions or including additional loci, as demonstrated in our study.

To our knowledge, this is the first phylogenomic study of the *Dipodascales* with comprehensive taxon sampling. Our results also highlight systematic inconsistencies in public databases. These include the incorrect placement of several *Dipodascus* species within *Geotrichum*, the need to merge particular *Candida* species (e.g., *C. lundiana*) into *Zygoascus*, and the polyphyletic nature of *Wickerhamiella* (Fig. 4).

The mycobiota of *T. oxyurus* was first described by Cassier et al. (1996), however, they did not deposit strains and did not provide either DNA data or detailed morphological observations. From female mycangia, they reported a *Graphium* sp. that was morphologically similar to, but distinct from, *Ophiostoma montium*. This morphology differs from that of *Wilhelmdebeerea* but closely resembles that of *Ophiostoma pityokteinis*, which we identified. Further studies on this beetle in Croatia (Meštrović 2023) used ITS barcoding to investigate cultures from beetle individuals and galleries. However, colony-forming units were not counted and, therefore, quantitative data on fungal abundances are lacking. The most frequently isolated fungi included *Meyerozyma guilliermondii*, *Graphilbum fragrans*, *Fomitopsis pinicola*, and *Magnusiomyces fungicola*. The author suggested that *M. guilliermondi* is probably an ambrosia fungus of *T. oxyurus*, as it was most frequently isolated from both adults and beetle galleries.

In our study, the dominant fungus in the mycangia was *W. oxyuri*, followed by two yeast species. According to the literature, both yeasts are rare and can be characterised as entomophilous and fungicolous. The first of these, *Candida schatavii*, has retained the genus name *Candida* as a valid name (www.mycobank.org), but phylogenetic analyses have suggested that it is a member of the genus *Kurtzmaniella* (Lopes et al. 2019; Schoch et al. 2020; Arrey et al. 2021). The type strain of *Candida schatavii* was isolated from a fruiting body of *Fomitopsis pinicola* in Czechia (Kocková-Kratochvílová and Ondrušová 1971). The only other verified findings, supported by DNA data, are from frass in Cerambycid beetle tunnels in a *Betula* tree in Moscow Oblast, Russia (GenBank accession no. PP410359) (Kachalkin et al. 2024), and from a grasshopper in Bulgaria (Dimitrov et al. 2024). *Magnusiomyces fungicola* has so far been found only in ascocarps of *Nectria cinnabarina* (de Hoog and Smith 2004). The only other record of this species in the literature, NCBI GenBank, or UNITE is from *T. oxyurus* in Croatia (Meštrović 2023).

The other fungus isolated, *O. pityokteinis*, has also been previously isolated from *Abies alba*, specifically from a bark beetle, and is currently known only from Poland near the border with Slovakia (Jankowiak et al. 2019). Thus, its presence in *Abies* and in association with *T. oxyuri* is not surprising.

All well-researched platypodine beetles have been found to engage in symbiosis with the genus *Raffaelea*, often with multiple species (Mayers et al. 2022). No such fungus was isolated in our study. Difficulties in identifying the primary ambrosial symbiont are widespread in the literature on *Platypodinae* ambrosia beetles. The issue is both methodological and biological. Most current and historical approaches have failed to distinguish between beetle tissues and their microhabitats, making it unclear whether fungal isolates originated from the mycangium, other body parts such as the gut or surface, or from the surrounding wood, which often harbours numerous fungi that do not contribute nutritionally to the beetle (Villari 2025). The biological difficulty stems from the ontogeny of the symbiotic interaction. Evidence is emerging that in most, if not all, ambrosia beetles, the mycangium only contains viable inoculum of the symbiont in young females, particularly during transport (Hulcr and Skelton 2023). In other life stages, the symbiont is often missing, overshadowed by other fungi. Isolation from indiscriminate body parts and life stages, therefore, produces fungi that can be positively misleading. The primary symbiont of platypodine beetles can be determined. Still, it requires repeated, quantitative sampling from mycangia at the appropriate developmental stage, ideally corroborated by replicated, quantitative sampling from ephemeral, active galleries (Li et al. 2018; Skelton et al. 2018). These conditions were only partially available in this study; they will be examined in greater detail in the future.

The specificity of the association between identified fungi and the beetle remains unclear, given the discordant records of identical DNA markers in samples outside the beetle’s range. Our fungal community also differed from those found in *T. oxyurus* in Croatia or France, except for *M. fungicola* in Croatia. The fact that two of its three known findings are associated with *T. oxyurus* suggests a non-random, strong association that warrants further study. The co-occurrence of *C. schatavii*, *B. sasensis*, and *W. oxyuri* is certainly not coincidental, as these rare fungi were found together despite being almost absent among the 84,972 samples in the GlobalFungi database.

Our work represents the first detailed study of a scarce European ambrosia beetle, unique in both its distribution and ecology. The abundance of several new or poorly known fungal species suggests that the ambrosia beetle ecosystem continues to yield discoveries. The disjunct distribution of the previous findings and the absence of ambrosia morphology in these fungi suggest that either *T. oxuyrus* vectors unorthodox symbionts or that the search for the *bona fide* mutualist continues.

## Supplementary Material

XML Treatment for Wilhelmdebeerea

XML Treatment for Wilhelmdebeerea oxyuri

XML Treatment for Blastobotrys sasensis

XML Treatment for Sugiyamaella casensis

## Additional information

### Use of AI

I consulted AI tools to improve the grammar and style of my manuscript.

### Adherence to national and international regulations

All the fungal strains used in this study have been legally obtained, respecting the Convention on Biological Diversity (Rio Convention).

### Author ORCIDs

Miroslav Kolařík https://orcid.org/0000-0003-4016-0335

Renata Vadkertiová https://orcid.org/0000-0002-1401-5604

Miloš Knížek https://orcid.org/0000-0003-3221-9448

František Sklenář https://orcid.org/0000-0001-8714-8558

Jozef Vakula https://orcid.org/0000-0001-8662-1817

Milan Zúbrik https://orcid.org/0000-0003-3627-5121

Michal Kolář https://orcid.org/0000-0002-4593-1525

Jiří Hulcr https://orcid.org/0000-0002-8706-4618

### Data availability

All of the data that support the findings of this study are available in the main text or Supplementary Information.

## Supplementary materials

Supplementary material 1 DNA sequences (ITS, LSU rDNA, TEF1α, RPB2) used in the molecular phylogenetic analysis of *Wilhelmdebeerea* Data type docx Explanation note List of DNA sequences (ITS, LSU rDNA, TEF1α, RPB2) used for the molecular phylogenetic analysis of *Wilhelmdebeerea*. https://binary.pensoft.net/file/1542062This dataset is made available under the Open Database License (http://opendatacommons.org/licenses/odbl/1.0/). The Open Database License (ODbL) is a license agreement intended to allow users to freely share, modify, and use this Dataset while maintaining this same freedom for others, provided that the original source and author(s) are credited. Miroslav Kolařík, Renata Vadkertiová, Miloš Knížek, František Sklenář, Jozef Vakula, Milan Zúbrik, Michal Kolář, Jiří Hulcr

Supplementary material 2 List of whole genomic sequences used in the phylogenomic study of *Wilhelmdebeerea* Data type docx Explanation note List of whole-genome sequences included in the phylogenomic study of *Wilhelmdebeerea*. https://binary.pensoft.net/file/1542063This dataset is made available under the Open Database License (http://opendatacommons.org/licenses/odbl/1.0/). The Open Database License (ODbL) is a license agreement intended to allow users to freely share, modify, and use this Dataset while maintaining this same freedom for others, provided that the original source and author(s) are credited. Miroslav Kolařík, Renata Vadkertiová, Miloš Knížek, František Sklenář, Jozef Vakula, Milan Zúbrik, Michal Kolář, Jiří Hulcr

Supplementary material 3 DNA sequences (ITS, LSU rDNA) used in the molecular phylogenetic analysis of *Blastobotrys* Data type docx Explanation note List of DNA sequences (ITS, LSU rDNA) used for the molecular phylogenetic analysis of *Blastobotrys*. https://binary.pensoft.net/file/1542064This dataset is made available under the Open Database License (http://opendatacommons.org/licenses/odbl/1.0/). The Open Database License (ODbL) is a license agreement intended to allow users to freely share, modify, and use this Dataset while maintaining this same freedom for others, provided that the original source and author(s) are credited. Miroslav Kolařík, Renata Vadkertiová, Miloš Knížek, František Sklenář, Jozef Vakula, Milan Zúbrik, Michal Kolář, Jiří Hulcr

Supplementary material 4 DNA sequences (ITS, LSU rDNA) used in the molecular phylogenetic analysis of *Sugiyamaella* Data type docx Explanation note List of DNA sequences (ITS, LSU rDNA) used for the molecular phylogenetic analysis of *Sugiyamaella*. https://binary.pensoft.net/file/1542065This dataset is made available under the Open Database License (http://opendatacommons.org/licenses/odbl/1.0/). The Open Database License (ODbL) is a license agreement intended to allow users to freely share, modify, and use this Dataset while maintaining this same freedom for others, provided that the original source and author(s) are credited. Miroslav Kolařík, Renata Vadkertiová, Miloš Knížek, František Sklenář, Jozef Vakula, Milan Zúbrik, Michal Kolář, Jiří Hulcr

Supplementary material 5 List of whole genomic sequences used in the phylogenomic study of *Saccharomycetales* Data type docx https://binary.pensoft.net/file/1542066This dataset is made available under the Open Database License (http://opendatacommons.org/licenses/odbl/1.0/). The Open Database License (ODbL) is a license agreement intended to allow users to freely share, modify, and use this Dataset while maintaining this same freedom for others, provided that the original source and author(s) are credited. Miroslav Kolařík, Renata Vadkertiová, Miloš Knížek, František Sklenář, Jozef Vakula, Milan Zúbrik, Michal Kolář, Jiří Hulcr

Supplementary material 6 Full alignment of ITS, LSU rDNA, TEF1α and RPB2 sequences used for phylogenetic analysis of *Wilhelmdebeerea* Data type fasta Explanation note Full alignment of ITS, LSU rDNA, TEF1α and RPB2 sequences for *Wilhelmdebeerea* phylogenetic analysis. https://binary.pensoft.net/file/1542067This dataset is made available under the Open Database License (http://opendatacommons.org/licenses/odbl/1.0/). The Open Database License (ODbL) is a license agreement intended to allow users to freely share, modify, and use this Dataset while maintaining this same freedom for others, provided that the original source and author(s) are credited. Miroslav Kolařík, Renata Vadkertiová, Miloš Knížek, František Sklenář, Jozef Vakula, Milan Zúbrik, Michal Kolář, Jiří Hulcr

Supplementary material 7 Curated alignment of ITS, LSU rDNA, TEF1α and RPB2 sequences used for phylogenetic analysis of *Wilhelmdebeerea* Data type fas Explanation note Curated alignment of ITS, LSU rDNA, TEF1α and RPB2 sequences for *Wilhelmdebeerea* phylogenetic analysis. https://binary.pensoft.net/file/1542068This dataset is made available under the Open Database License (http://opendatacommons.org/licenses/odbl/1.0/). The Open Database License (ODbL) is a license agreement intended to allow users to freely share, modify, and use this Dataset while maintaining this same freedom for others, provided that the original source and author(s) are credited. Miroslav Kolařík, Renata Vadkertiová, Miloš Knížek, František Sklenář, Jozef Vakula, Milan Zúbrik, Michal Kolář, Jiří Hulcr

Supplementary material 8 Full alignment of ITS and LSU rDNA sequences used for phylogenetic analysis of *Blastobotrys* Data type fas Explanation note Full alignment of ITS and LSU rDNA sequences for *Blastobotrys* phylogenetic analysis. https://binary.pensoft.net/file/1542069This dataset is made available under the Open Database License (http://opendatacommons.org/licenses/odbl/1.0/). The Open Database License (ODbL) is a license agreement intended to allow users to freely share, modify, and use this Dataset while maintaining this same freedom for others, provided that the original source and author(s) are credited. Miroslav Kolařík, Renata Vadkertiová, Miloš Knížek, František Sklenář, Jozef Vakula, Milan Zúbrik, Michal Kolář, Jiří Hulcr

Supplementary material 9 Curated alignment of ITS and LSU rDNA sequences used for phylogenetic analysis of *Blastobotrys* Data type fas Explanation note Curated alignment of ITS and LSU rDNA sequences for *Blastobotrys* phylogenetic analysis. https://binary.pensoft.net/file/1542070This dataset is made available under the Open Database License (http://opendatacommons.org/licenses/odbl/1.0/). The Open Database License (ODbL) is a license agreement intended to allow users to freely share, modify, and use this Dataset while maintaining this same freedom for others, provided that the original source and author(s) are credited. Miroslav Kolařík, Renata Vadkertiová, Miloš Knížek, František Sklenář, Jozef Vakula, Milan Zúbrik, Michal Kolář, Jiří Hulcr

Supplementary material 10 Full alignment of ITS and LSU rDNA sequences used for phylogenetic analysis of *Sugiyamaella* Data type fas Explanation note Full alignment of ITS and LSU rDNA sequences for *Sugiyamaella* phylogenetic analysis. https://binary.pensoft.net/file/1542071This dataset is made available under the Open Database License (http://opendatacommons.org/licenses/odbl/1.0/). The Open Database License (ODbL) is a license agreement intended to allow users to freely share, modify, and use this Dataset while maintaining this same freedom for others, provided that the original source and author(s) are credited. Miroslav Kolařík, Renata Vadkertiová, Miloš Knížek, František Sklenář, Jozef Vakula, Milan Zúbrik, Michal Kolář, Jiří Hulcr

Supplementary material 11 Curated alignment of ITS and LSU rDNA sequences used for phylogenetic analysis of *Sugiyamaella* Data type fasta Explanation note Curated alignment of ITS and LSU rDNA sequences for *Sugiyamaella* phylogenetic analysis. https://binary.pensoft.net/file/1542072This dataset is made available under the Open Database License (http://opendatacommons.org/licenses/odbl/1.0/). The Open Database License (ODbL) is a license agreement intended to allow users to freely share, modify, and use this Dataset while maintaining this same freedom for others, provided that the original source and author(s) are credited. Miroslav Kolařík, Renata Vadkertiová, Miloš Knížek, František Sklenář, Jozef Vakula, Milan Zúbrik, Michal Kolář, Jiří Hulcr

Supplementary material 12 Geography of main mycangial symbionts, studied using GlobalFungi database Data type docx https://binary.pensoft.net/file/1542073This dataset is made available under the Open Database License (http://opendatacommons.org/licenses/odbl/1.0/). The Open Database License (ODbL) is a license agreement intended to allow users to freely share, modify, and use this Dataset while maintaining this same freedom for others, provided that the original source and author(s) are credited. Miroslav Kolařík, Renata Vadkertiová, Miloš Knížek, František Sklenář, Jozef Vakula, Milan Zúbrik, Michal Kolář, Jiří Hulcr

Supplementary material 13 Physiological characteristic of the new yeast species Data type docx Explanation note **table SS1**. Physiological and biochemical properties of new species. **table SS2**. Comparison of physiological and biochemical properties of two new species with their closely related species. Physiological and biochemical characteristics of new yeast species and comparison with closely related taxa. https://binary.pensoft.net/file/1542074This dataset is made available under the Open Database License (http://opendatacommons.org/licenses/odbl/1.0/). The Open Database License (ODbL) is a license agreement intended to allow users to freely share, modify, and use this Dataset while maintaining this same freedom for others, provided that the original source and author(s) are credited. Miroslav Kolařík, Renata Vadkertiová, Miloš Knížek, František Sklenář, Jozef Vakula, Milan Zúbrik, Michal Kolář, Jiří Hulcr
