# Supplementary material for: The ambrosial mycobiota of Treptoplatypus oxyurus (Coleoptera, Platypodidae): a unique island of fungal diversity revealing Wilhelmdebeerea oxyuri gen. et sp. nov. (Ophiostomatales), and two new yeast species Blastobotrys sasensis sp. nov., and Sugiyamaella casensis sp. nov. (Dipodascales)
Source: IMA Fungus. 2026 Feb 16;17:e177075. doi: 10.3897/imafungus.17.177075 (PMC12930180; doi:10.3897/imafungus.17.177075)
Supplement: Supplementary material 1 — DNA sequences (ITS, LSU rDNA, TEF1α, RPB2) used in the molecular phylogenetic analysis of Wilhelmdebeerea [file imafungus-17-e177075-s001.docx]

**Supplementary table 1.** DNA sequences used in the molecular phylogenetic analysis of *Wilhelmdebeerea*.

| **Current name** | **Strain** | **Type** | **Isolated from** | **Locality** | **Sequence Accession no.** | | | | **Reference** |
| --- | --- | --- | --- | --- | --- | --- | --- | --- | --- |
|  |  |  |  |  | **ITS** | **LSU** | **TEF1-α** | **RPB2** |  |
| *Aureovirgo volantis* | CBS139649 | P | *Cyrtogenius* *africus* on *Euphorbia ingens* | South Africa | OM501369 | OM514700 | OM631743 | OM631579 | (de Beer et al. 2022) |
| *Ceratocystiopsis* *ranaculosa* | CBS119683 | T | *Pinus echinata* | North Carolina, USA | OM501375 | OM514705 | OM631748 | OM631584 | (de Beer et al. 2022) |
| *C. neglecta* | CBS100596 |  | *Hylurgops palliatus* | Germany | OM501373 | OM514704 | OM631746 | OM631582 | (de Beer et al. 2022) |
| *Dryadomyces* *amasae* | CBS116694 | T | *Amasa concitatus* on angiosperm | Taiwan | - | MT629750 | OM631750 | OM631585 | (de Beer et al. 2022) |
| *D. sulphureus* | CBS380.68 |  | *Xyleborus saxesenii* gallery in *Populus* *deltoides* | Kansas, USA | MT633077 | MT629768 | OM631755 | OM631589 | (Procter et al. 2020; de Beer et al. 2022) |
| *Esteya vermicola* | CBS115803 |  | *Scolytus intricatus* on *Quercus* | Czech Republic | OM501377 | OM514707 | OM631756 | OM631590 | (de Beer et al. 2022) |
| *Graphilbum crescericum* | CBS130864 | T | *Hylurgops palliatus* on *Pinus radiata* | Spain | OM501403 | OM514749 | OM631779 | OM631604 | (de Beer et al. 2022) |
| *Gra. microcarpum* | YCC439 | T | *Cryphalus montanus* | Japan | OM501405 | OM514751 | OM631781 | OM631606 | (de Beer et al. 2022) |
| *Gra. fragrans* | CBS138720 | T | *Pinus patula* | South Africa | OM501404 | OM514750 | OM631780 | OM631605 | (de Beer et al. 2022) |
| *Gra. sparsum* | CBS405.77 | T | bark beetle gallery on *Picea glauca* | Alaska, USA | OM501409 | OM514755 | - | OM631608 | (de Beer et al. 2022) |
| *Gra. nigrum* | CBS163.61 | A | *Abies lasiocarpa* | Colorado, USA | OM501406 | OM514752 | - | OM631607 | (de Beer et al. 2022) |
| *Gra. acuminatum* | CBS145828 | T | *Ips acuminatus* gallery on *Pinus sylvestris* | Poland | MN548902 | - | MN548952 | - | (Jankowiak et al. 2020) |
| *Gra. ipis-grandicollis* | VPRI43762 |  | *Ips grandicollis* gallery on *Pinus* *radiata* | Australia | MW046071 | MW046117 | MW066405 | - | (Trollip et al. 2021) |
| *Grosmannia americana* | CBS497.96 | T | *Dendroctonus simplex* on *Larix* *laricina* | Vermont, USA | OM501385 | OM514718 | OM631765 | OM631595 | (de Beer et al. 2022) |
| *G. aoshimae* | MAFF238948 | T | *Polygraphus* *proximus* on *Abies* *mariesii* | Japan | OM501386 | OM514719 | OM631766 | OM631596 | (de Beer et al. 2022) |
| *G. dryocoetis* | CBS376.66 | T | *Dryocoetes confusus* on *Abies* *lasiocarpa* | Canada | OM501392 | OM514727 | OM631768 | OM631597 | (de Beer et al. 2022) |
| *G. maixiuense* | CBS136502 | T | *Polygraphus poligraphus*, *Ips shangrila* in *Picea* *crassifolia* | China | OM501396 | MN644474 | MN647900 | OM631599 | (Yin et al. 2020; de Beer et al. 2022) |
| *G. penicillata* | CBS116008 |  | *Picea abies* | Norway | OM501397 | OM514737 | OM631774 | OM631600 | (de Beer et al. 2022) |
| *G. abietina* | CBS118590 | T | *Picea engelmannii* | Canada | OM501383 | OM514713 | OM631763 | OM631594 | (de Beer et al. 2022) |
| *G. hughesii* | CBS109709 | T | *Aquilaria* sp. | Vietnam | OM501395 | OM514732 | OM631772 | OM631598 | (de Beer et al. 2022) |
| *G. curvispora* | CBS123914 | T | *Picea abies* | Norway | OM501391 | OM514726 | EU979347 | - | (Jacobs et al. 2010; de Beer et al. 2022) |
| *G. zekuensis* | CBS141901 | T | *Bakerdania* sp. in gallery of *Ips nitidus* | China | MH121683 | MH121683 | MH124546 | - | (Chang et al. 2020) |
| *G. betulae* | CBS142734 | T | *Scolytus ratzeburgi* on *Betula* *verrucosa* | Poland | KY801840 |  | KY801817 | - | (Jankowiak et al. 2017) |
| *G. pruni* | CBS120197 | P | *Polygraphus ssiori* on *Prunus* *jamasakura* | Japan | OM501398 | OM514740 | OM631775 | - | (de Beer et al. 2022) |
| *G. abieticola* | CMW17199 | T | *Dryocoetes hectographus* on *Abies* *mariesii* | Japan | OM501382 | OM514712 | OM631761 | - | (de Beer et al. 2022) |
| *G. taigensis* | CMW36629 |  | *Ips typographus* on *Picea abies* | Russia | OM501400 | OM514744 | OM631777 | - | (de Beer et al. 2022) |
| *G. gestamen* | CIEFAP453 | T | *Nothofagus dombeyi* | Argentina | KT362234 | KT362232 | KT381300 | - | (de Errasti et al. 2016) |
| *Harringtonia* *aguacate* | Raff. sp. 272 |  | *Persea americana* | Florida, USA | MT633065 | MT629748 | OM631783 | OM631613 | (Procter et al. 2020; de Beer et al. 2022) |
| *Har. lauricola* | Raff. sp. 570 |  | *Xyleborus* sp. on *Persea* sp. | Florida, USA | MT633071 | MT629759 | OM631784 | OM631614 | (Procter et al. 2020; de Beer et al. 2022) |
| *Hausneria geniculata* | CBS 151303 |  | *Dryocoetes alni* on *Alnus incana* | Norway | PP410007 | PP410007 | PP400438 | - | (Crous et al. 2024) |
| *Hau. geniculata* | N2015-1555/2/3/2 |  | *Dryocoetes alni* on *Alnus incana* | Norway | PP410008 | PP410008 | PP400439 | - | (Crous et al. 2024) |
| *Hawksworthiomyces crousii* | MUCL55928 | T | Bamboo chips | South Korea | KX396551 | KX396548 | OM652622 | OM631609 | (De Beer et al. 2016; de Beer et al. 2022) |
| *Haw. hibbettii* | MUCL55929 | T | *Trachymyrmex* sp. | Texas, USA | KX396550 | KX396547 | OM652623 | OM631610 | (De Beer et al. 2016; de Beer et al. 2022) |
| *Haw. taylorii* | MUCL55927 | T | *Eucalyptus* pole | South Africa | KX396549 | KX396546 | OM652624 | OM631612 | (De Beer et al. 2016; de Beer et al. 2022) |
| *Heinzbutinia grandicarpa* | CBS250.88 | T | *Quercus* *robur* | Poland | OM501412 | OM514757 | OM631786 | OM631616 | (de Beer et al. 2022) |
| *He. microspora* | CBS440.69 | T | *Quercus* sp. | Virginia, USA | - | OM514758 | OM631787 | OM631617 | (de Beer et al. 2022) |
| *Intubia macrotermitinarum* | CBS141560 | T | *Termitomyces* fungal comb of *Macrotermes* *natalensis* | South Africa | MT637025 | - | - | - | (Nel et al. 2021) |
| *I. oerlemansii* | CBS47048 | T | *Termitomyces* fungal comb of *Macrotermes* *natalensis* | South Africa | MT637024 | - | extracted from WGS GCA_020002355.1 (CBS:141565) | extracted from WGS GCA_020002355.1 (CBS:141565) | (Nel et al. 2021) |
| *Jamesreidia coronata* | UAMH9685 | T | *Pinus* sp. | Canada | OM501413 | OM514759 | OM631788 | OM631618 | (de Beer et al. 2022) |
| *J. tenella* | CBS189.86 |  | *Pinus* sp. | Colorado, USA | OM501414 | OM514760 | OM631789 | - | (de Beer et al. 2022) |
| *Leptographium. aureum* | CBS438.69 | A | *Pinus* *contorta* var. *latifolia* | Canada | OM501387 | OM514720 | OM631793 | OM631621 | (de Beer et al. 2022) |
| *L. cucullatum* | CBS218.83 | T | *Ips typographus* | Norway | OM501423 | OM514724 | OM631801 | OM631626 | (de Beer et al. 2022) |
| *L. olivaceapini* | CBS504.86 | E | *Pinus ponderosa* in *Dendroctonus* sp. | Arizona, USA | OM501433 | OM514736 | OM631810 | OM631635 | (de Beer et al. 2022) |
| *L. olivaceum* | CMW23348 |  | *Pinus sylvestris* | Finland | OM501434 | OM514735 | OM631811 | - | (de Beer et al. 2022) |
| *L. pyrinum* | CBS120181 | T | *Dendroctonus* *adjunctus* | USA | OM501445 | OM514781 | OM631819 | OM631642 | (de Beer et al. 2022) |
| *L. galeiforme* | CBS115711 | E | *Pinus* *sylvestris* | Scotland | OM501428 | OM514731 | OM631806 | OM631631 | (de Beer et al. 2022) |
| *L. radiaticola* | CMW9482 |  | *Hylurgus* *ligniperda* on *Pinus radiata* | Chile | OM501446 | OM514742 | OM631820 | OM631643 | (de Beer et al. 2022) |
| *L. koreanum* | KUC2078 | T | *Tomicus piniperda* on *Pinus* *koraiensis* | South Korea | OM501431 | OM514733 | OM631808 | OM631633 | (de Beer et al. 2022) |
| *L. piceiperdum* | CBS138719 |  | *Picea glauca* | Canada | OM501435 | OM514738 | OM631812 | OM631636 | (de Beer et al. 2022) |
| *L. procerum* | CBS138288 | E | *Dendroctonus valens* on *Pinus resinosa* | Maine, USA | OM501442 | OM514778 | OM631816 | OM631639 | (de Beer et al. 2022) |
| *L. alacre* | CBS128830 | T | *Pinus pinaster* | Portugal | OM501417 | OM514717 | OM631792 | OM631620 | (de Beer et al. 2022) |
| *L. wageneri var. ponderosum* | CMW279 |  | *Pinus* sp. | USA | OM501458 | OM514746 | OM631831 | OM631651 | (de Beer et al. 2022) |
| *L. cainii* | CMW24907 |  | *Picea* sp. | Canada | OM501389 | OM514722 | OM631797 | OM631622 | (de Beer et al. 2022) |
| *L. verrucosum* | CBS112420 | T | *Xyleborus dryographus* | Germany | OM501456 | OM514787 | OM631830 | OM631650 | (de Beer et al. 2022) |
| *Masuyamyces ambrosius* | CBS210.64 |  | wood of *Pinus sylvestris* | Netherlands | OM501465 | OM514793 | OM631836 | OM631656 | (de Beer et al. 2022) |
| *M. botuliformis* | CMW14493 |  | *Cryphalus jeholensis* | Japan | OM501471 | OM514799 | OM631837 | - | (de Beer et al. 2022) |
| *M. saponiodorus* | CBS128302 |  | *Pinus sylvestris* | Russia | OM501512 | OM514838 | OM631838 | OM631657 | (de Beer et al. 2022) |
| *Ophiostoma ainoae* | CBS205.83 | T | *Picea abies* | Norway | OM501463 | OM514791 | - | - | (de Beer et al. 2022) |
| *O. brunneolum* | CMW23145 | A | *Picea abies* | Russia | OM501472 | OM514800 | OM631843 | OM631663 | (de Beer et al. 2022) |
| *O. ips* | CBS138721 |  | *Pinus taeda* | Louisiana, USA | OM501486 | OM514812 | OM631853 | OM631671 | (de Beer et al. 2022) |
| *O. minus* | UAMH4917 |  | *Dendroctonus ponderosae* on *Pinus flexillis* | Canada | OM501497 | OM514821 | OM631860 | OM631677 | (de Beer et al. 2022) |
| *O. piceae* | UAMH11346 |  | Pine saw timber | Canada | MT633062 | MT629745 | OM631869 | OM631686 | (Procter et al. 2020; de Beer et al. 2022) |
| *O. sparsiannulatum* | CBS122815 |  | *Pinus taeda* | Georgia, USA | OM501515 | OM514841 | OM631880 | OM631696 | (de Beer et al. 2022) |
| *O. novo-ulmi s* | UAMH10443 |  | *Ulmus* sp. | Iran | OM501504 | OM514829 | OM631866 | OM631684 | (de Beer et al. 2022) |
| *O. triangulosporum* | CBS138.77 | T | *Araucaria angustifolia* | Brazil | OM501519 | OM514845 | OM631884 | OM631700 | (de Beer et al. 2022) |
| *O. tetropii* | CBS428.94 |  | *Picea abies* | Austria | OM501517 | OM514843 | OM631882 | OM631698 | (de Beer et al. 2022) |
| *O. denticulatum* | ATCC38087 |  | *Gnathotrichus* sp. on *Pinus* sp. | Colorado, USA | OM501480 | OM514807 | - | - | (de Beer et al. 2022) |
| *O. angusticollis* | CMW152 |  | *Pinus banksiana* | Wisconsin, USA | OM501466 | OM514794 | OM631840 | OM631660 | (de Beer et al. 2022) |
| *R. albimanens* | CBS271.7 | T | *Platypus externedentatus* in *Ficus sycomorus* | South Africa | MT633066 | MT629749 | OM631890 | OM631705 | (Procter et al. 2020; de Beer et al. 2022) |
| *R. ambrosiae* | CBS185.64 | T | *Platypus cylindrus* tunnel in *Quercus* sp. | England, UK | MT633067 | MT629751 | OM631891 | OM631706 | (Procter et al. 2020; de Beer et al. 2022) |
| *R. canadensis* | CBS168.66 | T | *Platypus wilsonii* in *Pseudotsuga menziessii* | Canada | GQ225699 | MT629755 | - | - | (Kim et al. 2009; Procter et al. 2020) |
| *R. seticollis* | CBS634.66 | T | *Tsuga canadensis* | New York, USA | MT633076 | MT629766 | OM631895 | OM631711 | (Procter et al. 2020; de Beer et al. 2022) |
| *R. deltoideospora* | SCAU 21GD01071 |  | *Pinus* sp. | Canada | PP760100 | PP792734 | PP819561 | - | Chang et al. (2021) |
| *R. vaginata* | CBS140086 | T | *Lanurgus* sp. on *Olea capensis* | South Africa | KT192602 | KT182932 | - | - | (Musvuugwa et al. 2015) |
| *Sporothrix aemulophila* | CBS140087 | T | *Rapanea melanophloeos* | South Africa | OM501527 | OM514854 | OM631897 | - | (de Beer et al. 2022) |
| *S. cabralii* | CIEFAP456 | T | *Nothofagus pumilio* | Argentina | OM501533 | OM514861 | OM631903 | OM631716 | (de Beer et al. 2022) |
| *S. rapaneae* | CBS141060 | T | *Rapanea melanophloeos* | South Africa | OM501558 | OM514885 | OM631926 | OM631735 | (de Beer et al. 2022) |
| *S. dentifunda* | CBS115790 | T | *Quercus* wood | Hungary | OM501535 | OM514865 | OM631907 | OM631718 | (de Beer et al. 2022) |
| *S. dimorphospora* | CBS553.74 | T | soil |  | OM501536 | OM514866 | OM631908 | OM631719 | (de Beer et al. 2022) |
| *S. abietina* | CBS125.89 | T | *Pseudohylesinus gallery on Abies vejari* | Mexico | OM501526 | OM514853 | OM631896 | OM631713 | (de Beer et al. 2022) |
| *S. eucastaneae* | CBS424.77 | T | Canker on *Castanea dentata* | North Carolina, USA | OM501539 | OM514868 | OM631910 | OM631720 | (de Beer et al. 2022) |
| *S. protearum* | CBS116654 |  | *Protea caffra* | South Africa | OM501557 | OM514884 | OM631925 | OM631734 | (de Beer et al. 2022) |
| *S. zambiensis* | CBS124914 | T | *Protea caffra* | Zambia | OM501567 | OM514894 | OM631932 | OM631741 | (de Beer et al. 2022) |
| *S. pallida* | CBS131.56 | T | *Stemonitis fusca* | Japan | OM501550 | OM514878 | OM631921 | OM631729 | (de Beer et al. 2022) |
| *S. palmiculminata* | CBS119590 | T | *Protea repens* | South Africa | OM501551 | OM514879 | OM631922 | OM631730 | (de Beer et al. 2022) |
| *S. globosa* | CBS120340 | T | *Human face* | Spain | OM501541 | OM514872 | OM631913 | OM631723 | (de Beer et al. 2022) |
| *S. schenckii* | CBS138723 |  | clinical isolate | South Africa | OM501560 | OM514887 | OM631928 | OM631737 | (de Beer et al. 2022) |
| *S. phasma* | CBS119722 | T | *Protea laurifolia* | South Africa | OM501552 | OM514880 | OM631923 | OM631731 | (de Beer et al. 2022) |
| *S. curviconia* | CBS959.73 | T | *Terminalia ivorensis* | Ivory Coast | OM501534 | OM514864 | OM631906 | - | (de Beer et al. 2022) |
| *S. dombeyi* | CBS455.83 | T | *Nothofagus* sp. | Chile | OM501537 | OM514867 | - | - | (de Beer et al. 2022) |
| *S. fumea* | CBS129712 | T | *Phoracantha* sp. galleries on *Eucalyptus* | South Africa | OM501540 | OM514869 | OM631911 | OM631721 | (de Beer et al. 2022) |
| *S. brunneoviolacea* | CBS124560 | P | soil | Spain | OM501532 | OM514860 | OM631902 | - | (de Beer et al. 2022) |
| *S. hypoxyli* | CBS141569 | T | *Hypoxylon petriniae* on *Fraxinus* wood | Netherlands | MT637058 | MW012948 | - | - | (Crous et al. 2021; Nel et al. 2021) |
| ***Wilhelmdebeerea oxyuri*** | CCF6802 | T | *Treptoplatypus oxyurus* | Slovakia | PX523832 | [PV061845](https://www.ncbi.nlm.nih.gov/nuccore/PV061845) | PV067588 | PV067587 | This study |
| *Pyricularia grisea* | CBS138707 and Y34 | T | *Digitaria* sp. | Delaware, UDSA | NR_172230 | MH877665 | AY849694 | OL496643 | (Klaubauf et al. 2014; Vu et al. 2019) |
| *Fragosphaeria*  *purpurea* | CBS133.34 | A | *Fagus* sp. | England, UK | OM501379 | OM514710 | OM631759 | OM631592 | (de Beer et al. 2022) |
| *F. reniformis* | CBS134.34 | A | *Fagus* sp. | England, UK | OM501381 | - | OM631760 | OM631593 | (de Beer et al. 2022) |
| *Ophiostoma valdivianum* | CMW449 | T | *Nothofagus alpiva* | Chile | - | OM514849 | OM631886 | - | (de Beer et al. 2022) |

**Taxa excluded from the final analysis**

| *Afroraffaelea ambrosiae* | CBS141678 | T | *Premnobius cavipennis* | Florida, USA | OM632703 | OM584293 | OM631576 | OM631577 | (de Beer et al. 2022) |
| --- | --- | --- | --- | --- | --- | --- | --- | --- | --- |
| *Chrysosphaeria jan-nelii* | CBS141570 | T | *Termitomyces fungal* comb of *Macrotermes natalensis* | South Africa W.J. Nel MT637038 MT637006 | MT637038 | MT637006 | - | - | (Nel et al. 2021) |

**References**

Crous PW, Hernández-Restrepo M, Schumacher R, Cowan DA, Maggs-Kölling G, Marais E, Wingfield MJ, Yilmaz N, Adan O, Akulov A (2021) New and interesting fungi. 4. Fungal Systematics and Evolution 7: 255. doi:10.3114/fuse.2021.07.13.

Crous PW, Jurjević Ž, Balashov S, De la Peña-Lastra S, Mateos A, Pinruan U, Rigueiro-Rodríguez A, Osieck ER, Altés A, Czachura P, Esteve-Raventós F, Gunaseelan S, Kaliyaperumal M, Larsson E, Luangsa-Ard JJ, Moreno G, Pancorbo F, Piątek M, Sommai S, Somrithipol S, Asif M, Delgado G, Flakus A, Illescas T, Kezo K, Khamsuntorn P, Kubátová A, Labuda R, Lavoise C, Lebel T, Lueangjaroenkit P, Maciá-Vicente JG, Paz A, Saba M, Shivas RG, Tan YP, Wingfield MJ, Aas T, Abramczyk B, Ainsworth AM, Akulov A, Alvarado P, Armada F, Assyov B, Avchar R, Avesani M, Bezerra JL, Bhat JD, Bilański P, Bily DS, Boccardo F, Bozok F, Campos JC, Chaimongkol S, Chellappan N, Costa MM, Dalecká M, Darmostuk V, Daskalopoulos V, Dearnaley J, Dentinger BTM, De Silva NI, Dhotre D, Carlavilla JR, Doungsa-Ard C, Dovana F, Erhard A, Ferro LO, Gallegos SC, Giles CE, Gore G, Gorfer M, Guard FE, Hanson SÅ, Haridev P, Jankowiak R, Jeffers SN, Kandemir H, Karich A, Kisło K, Kiss L, Krisai-Greilhuber I, Latha KPD, Lorenzini M, Lumyong S, Manimohan P, Manjón JL, Maula F, Mazur E, Mesquita NLS, Młynek K, Mongkolsamrit S, Morán P, Murugadoss R, Nagarajan M, Nalumpang S, Noisripoom W, Nosalj S, Novaes QS, Nowak M, Pawłowska J, Peiger M, Pereira OL, Pinto A, Plaza M, Polemis E, Polhorský A, Ramos DO, Raza M, Rivas-Ferreiro M, Rodriguez-Flakus P, Ruszkiewicz-Michalska M, Sánchez A, Santos A, Schüller A, Scott PA, Şen I, Shelke D, Śliwa L, Solheim H, Sonawane H, Strašiftáková D, Stryjak-Bogacka M, Sudsanguan M, Suwannarach N, Suz LM, Syme K, Taşkın H, Tennakoon DS, Tomka P, Vaghefi N, Vasan V, Vauras J, Wiktorowicz D, Villarreal M, Vizzini A, Wrzosek M, Yang X, Yingkunchao W, Zapparoli G, Zervakis GI, Groenewald JZ (2024) Fungal Planet description sheets: 1614-1696. Fungal Syst Evol. 13: 183-440. doi: 10.3114/fuse.2024.13.11.

De Beer ZW, Marincowitz S, Duong TA, Kim J-J, Rodrigues A, Wingfield MJ (2016) Hawksworthiomyces gen. nov.(Ophiostomatales), illustrates the urgency for a decision on how to name novel taxa known only from environmental nucleic acid sequences (ENAS). Fungal biology 120: 1323-1340.

de Beer ZW, Procter M, Wingfield MJ, Marincowitz S, Duong TA (2022) Generic boundaries in the Ophiostomatales reconsidered and revised. Studies in Mycology 101: 57-120. doi:10.3114/sim.2022.101.02.

de Errasti A, de Beer ZW, Coetzee M, Roux J, Rajchenberg M, Wingfield MJ (2016) Three new species of Ophiostomatales from Nothofagus in Patagonia. Mycological Progress 15: 17.

Chang R, Duong TA, Taerum SJ, Wingfield MJ, Zhou X, de Beer ZW (2020) Ophiostomatoid fungi associated with mites phoretic on bark beetles in Qinghai, China. IMA fungus 11: 15. doi:10.1186/s43008-020-00037-9.

Chang R, Zhang X, Si H, Zhao G, Yuan X, Liu T, Bose T, Dai M (2021) Ophiostomatoid species associated with pine trees (*Pinus* spp.) infested by *Cryphaluspiceae* from eastern China, including five new species. MycoKeys. 2021 Oct 13;83:181-208. doi: 10.3897/mycokeys.83.70925

Jacobs K, Krokene P, Solheim H, Wingfield MJ (2010) Two new species of *Leptographium* from *Dryocetes authographus* and *Hylastes cunicularius* in Norway. Mycological Progress 9: 69-78. doi:10.1007/s11557-009-0620-6.

Jankowiak R, Solheim H, Bilański P, Marincowitz S, Wingfield MJ (2020) Seven new species of *Graphilbum* from conifers in Norway, Poland, and Russia. doi:10.1080/00275514.2020.1778375.

Jankowiak R, Strzałka B, Bilański P, Linnakoski R, Aas T, Solheim H, Groszek M, de Beer ZW (2017) Two new Leptographium spp. reveal an emerging complex of hardwood-infecting species in the Ophiostomatales. Antonie Van Leeuwenhoek 110: 1537-1553.

Kim K-H, Choi Y-J, Seo S-T, Shin H-D (2009) Raffaelea quercus-mongolicae sp. nov. associated with Platypus koryoensis on oak in Korea. Mycotaxon 110: 189-197.

Klaubauf S, Tharreau D, Fournier E, Groenewald JZ, Crous PW, De Vries RP, Lebrun M-H (2014) Resolving the polyphyletic nature of *Pyricularia* (Pyriculariaceae). Studies in Mycology 79: 85-120. doi:10.1016/j.simyco.2014.09.004

Mullineux T, Hausner G (2009) Evolution of rDNA ITS1 and ITS2 sequences and RNA secondary structures within members of the fungal genera Grosmannia and Leptographium. Fungal Genetics and Biology 46: 855-867.

Musvuugwa T, De Beer ZW, Duong TA, Dreyer LL, Oberlander KC, Roets F (2015) New species of Ophiostomatales from Scolytinae and Platypodinae beetles in the Cape Floristic Region, including the discovery of the sexual state of Raffaelea. Antonie Van Leeuwenhoek 108: 933-950.

Nel WJ, De Beer ZW, Wingfield MJ, Poulsen M, Aanen DK, Wingfield BD, Duong TA (2021) Phylogenetic and phylogenomic analyses reveal two new genera and three new species of ophiostomatalean fungi from termite fungus combs. Mycologia 113: 1199-1217. doi:10.1080/00275514.2021.1950455

Procter M, Nel WJ, Marincowitz S, Crous PW, Wingfield MJ (2020) A new species of Raffaelea from beetle-infested Leucaena leucocephala. Fungal Systematics and Evolution 6: 305-314. doi:10.3114/fuse.2020.06.16.

Trollip C, Carnegie AJ, Dinh Q, Kaur J, Smith D, Mann R, Rodoni B, Edwards J (2021) Ophiostomatoid fungi associated with pine bark beetles and infested pines in south-eastern Australia, including Graphilbum ipis-grandicollis sp. nov. IMA fungus 12: 24. doi:10.1186/s43008-021-00076-w.

Vu D, Groenewald M, De Vries M, Gehrmann T, Stielow B, Eberhardt U, Al-Hatmi A, Groenewald J, Cardinali G, Houbraken J (2019) Large-scale generation and analysis of filamentous fungal DNA barcodes boosts coverage for kingdom fungi and reveals thresholds for fungal species and higher taxon delimitation. Studies in Mycology 92: 135-154.

Yin M, Wingfield MJ, Zhou X, de Beer ZW (2020) Phylogenetic re-evaluation of the *Grosmannia penicillata* complex (Ascomycota, Ophiostomatales), with the description of five new species from China and USA. Fungal biology 124: 110-124. doi:10.1016/j.funbio.2019.12.003.
