# Supplementary material for: The ambrosial mycobiota of Treptoplatypus oxyurus (Coleoptera, Platypodidae): a unique island of fungal diversity revealing Wilhelmdebeerea oxyuri gen. et sp. nov. (Ophiostomatales), and two new yeast species Blastobotrys sasensis sp. nov., and Sugiyamaella casensis sp. nov. (Dipodascales)
Source: IMA Fungus. 2026 Feb 16;17:e177075. doi: 10.3897/imafungus.17.177075 (PMC12930180; doi:10.3897/imafungus.17.177075)
Supplement: Supplementary material 2 — List of whole genomic sequences used in the phylogenomic study of Wilhelmdebeerea [file imafungus-17-e177075-s002.docx]

**Supplementary material 2.** List of whole genomic sequences used in the phylogenomic study of *Wilhelmdebeerea*.

| **Current name** | **Strain no.** | **Assembly Accession** | **Number of Scaffolds** | **Reference** |
| --- | --- | --- | --- | --- |
| *Ceratocystiopsis brevicomis* | CBS 137839 | GCA_002778105.1 | 200 | Vanderpool et al. (2018) |
| *Ceratocystiopsis minuta* | CBS 138717 | GCA_001676865.1 | 903 |  |
| *Dryadomyces quercivorus* | CBS 122982 | GCA_002778125.1 | n.a. | Wingfield et al. (2016a) |
| *Dryadomyces quercivorus* | JCM 11526 | GCA_001662465.1 | 26 |  |
| *Dryadomyces quercus-mongolicae* | KACC44405 | GCA_002215975.1 | 43 | Vanderpool et al. (2018) |
| *Dryadomyces sulphureus* | CBS 380.68 | GCA_002778055.1 | 157 |  |
| *Esteya vermicola* | CBS 115803 | GCA_002778215.1 | n.a. | unpublished |
| *Fragosphaeria purpurea* | CBS 133.34 | GCA_002778095.1 | n.a. | Jeon et al. (2017) |
| *Graphilbum cf. rectangulosporium* | VPRI43763 | GCA_019925645.1 | 116 |  |
| *Graphilbum fragrans* | CBS 138720 | GCA_001513895.1 | 79 | Vanderpool et al. (2018) |
| *Graphilbum ipis-grandicollis* | VPRI43762 | GCA_019925625.1 | 177 |  |
| *Grosmannia clavigera* | kw1407 | GCA_000143105.2 | 289 | Vanderpool et al. (2018) |
| *Grosmannia huntii* | VPRI43530 | GCA_019925595.1 | 253 |  |
| *Grosmannia penicillata* | CBS 116008 | GCA_001938055.1 | 198 | Vanderpool et al. (2018) |
| *Grosmannia serpens* | CMW60 | GCA_029299595.1 | 689 |  |
| *Harringtonia aguacate* | PL1004 | GCA_014183095.1 | 368 | Trollip et al. (2021) |
| *Harringtonia lauricola* | CBS 129006 | GCA_004153705.1 | n.a. |  |
| *Hawksworthiomyces lignivorus* | CBS 119148 | GCA_021396355.1 | 12113 | Wingfield et al. (2015b) |
| *Chrysosphaeria jannelii* | CMW47058 | GCA_020002325.1 | 1002 |  |
| *Intubia oerlemansii* | CMW47056 | GCA_020002355.1 | 1058 | Trollip et al. (2021) |
| *Leptographium flavum* |  | GCA_963580335.1 | 7 |  |
| *Leptographium galeiforme* | CBS 115711 | GCA_004028395.1 | 868 | DiGuistini et al. (2011) |
| *Leptographium lundbergii* | CBS 138716 | GCA_001455505.1 | 411 |  |
| *Leptographium procerum* | CMW34542 | GCA_000806385.1 | 2687 | Trollip et al. (2021) |
| *Leptographium radiaticola* | VPRI43523 | GCA_019925535.1 | 84 |  |
| *Leptographium wageneri* | C730 | GCA_029299265.1 | n.a. | Wingfield et al. (2016b) |
| *Leptographium wingfieldii* | CBS645.89 | GCA_029299245.1 | 1101 |  |
| *Ophiostoma angusticollis* | VPRI43764 | GCA_019925545.1 | 316 | Fijarczyk et al. (2022) |
| *Ophiostoma australiae* | DAR52683 | GCA_022392945.1 | 27 |  |
| *Ophiostoma fasciatum* | VPRI43845 | GCA_019925495.1 | 35 | Zhang et al. (2020) |
| *Ophiostoma himal-ulmi* | HP32 | GCA_029299165.1 | n.a. |  |
| *Ophiostoma ips* | CBS 138721 | GCA_002917055.1 | 349 | Zhang et al. (2020) |
| *Ophiostoma montium* | DLS1121 | GCA_029299015.1 | 656 |  |
| *Ophiostoma novo-ulmi* subsp. *americana* | 04-544 | GCA_029298995.1 | n.a. | Huang et al. (2020) |
| *Ophiostoma novo-ulmi* subsp. *novo-ulmi* | Yu16 | GCA_029298875.1 | n.a. |  |
| *Ophiostoma pallidulum* | VPRI43846 | GCA_019925425.1 | 458 | Nel et al. (2021) |
| *Ophiostoma perfectum* | 703A | GCA_029298845.1 | 274 |  |
| *Ophiostoma piceae* | UAMH 11346 | GCA_000410735.1 | 45 | Nel et al. (2021) |
| *Ophiostoma populinum* | CBS212.67 | GCA_029298855.1 | n.a. |  |
| *Ophiostoma quercus* | MZ2-65 | GCA_029298795.1 | 140 | unpublished |
| *Ophiostoma sp. 15807* | SP_15807 | GCA_029298765.1 | 660 | unpublished |
| *Ophiostoma* sp. 423A | SP_423A | GCA_029298775.1 | n.a. | Wingfield et al. (2015a) |
| *Ophiostoma tasmaniense* | DAR52684 | GCA_022392925.1 | 45 |  |
| *Ophiostoma triangulosporum* | CBS138.77 | GCA_029298705.1 | n.a. | van der Nest et al. (2014) |
| *Raffaelea albimanens* | CBS 271.70 | GCA_002778245.1 | n.a. |  |
| *Raffaelea ambrosiae* | CBS 185.64 | GCA_002778195.1 | 68 | Trollip et al. (2021) |
| *Raffaelea arxii* | CBS 273.70 | GCA_002778165.1 | 124 |  |
| *Raffaelea deltoideospora* | VPRI43720 | GCA_019925385.1 | 114 | Fijarczyk et al. (2022) |
| *Raffaelea* sp. RL272 | RL272 | GCA_002777955.1 | 414 |  |
| *Sporothrix bragantina* |  | GCA_963921885.1 | n.a. | Fijarczyk et al. (2022) |
| *Sporothrix brasiliensis* | 5110 | GCA_000820605.1 | n.a. |  |
| *Sporothrix brasiliensis* | 5110 | GCF_000820605.1 | n.a. | Trollip et al. (2021) |
| *Sporothrix brunneoviolacea* | CBS 124561 | GCA_021396205.1 | 7065 |  |
| *Sporothrix cf. nigrograna* | VPRI43755 | GCA_019925305.1 | 124 | Trollip et al. (2022) |
| *Sporothrix curviconia* | CBS 959.73 | GCA_016097085.2 | n.a. |  |
| *Sporothrix dimorphospora* | CBS 553.74 | GCA_021397985.1 | 5177 | Trollip et al. (2021) |
| *Sporothrix epigloea* | CBS 119000 | GCA_963923935.1 | n.a. |  |
| *Sporothrix epigloea* | CBS 573.63 | GCA_963923925.1 | n.a. | Fijarczyk et al. (2022) |
| *Sporothrix eucalyptigena* |  | GCA_963921865.1 | n.a. |  |
| *Sporothrix euskadiensis* | VPRI43754 | GCA_019925375.1 | 91 | Wingfield et al. (2017) |
| *Sporothrix globosa* | CBS 120340 | GCA_001630435.1 | 24 |  |
| *Sporothrix globosa* | LC2404 | GCA_021396195.1 | 917 | Fijarczyk et al. (2022) |
| *Sporothrix globosa* | LC2445 | GCA_021396295.1 | 530 |  |
| *Sporothrix humicola* | CBS 118129 | GCA_021396245.1 | 1907 | Fijarczyk et al. (2022) |
| *Sporothrix inflata* | CBS 239.68 | GCA_021396225.1 | 1215 |  |
| *Sporothrix luriei* | CBS 937.72 | GCA_021398005.1 | 5177 | Fijarczyk et al. (2022) |
| *Sporothrix mexicana* | CBS 120341 | GCA_021396375.1 | 25656 |  |
| *Sporothrix pallida* | CBS 131.56 | GCA_021396235.1 | 1225 | Trollip et al. (2021) |
| *Sporothrix phasma* | CBS 119588 | GCA_016097075.2 | n.a. |  |
| *Sporothrix phasma* | CBS 119721 | GCA_011037845.1 | 277 | Fijarczyk et al. (2022) |
| *Sporothrix protearum* | CBS 116654 | GCA_016097115.2 | n.a. |  |
| *Sporothrix pseudoabietina* | VPRI43531 | GCA_019925295.1 | 50 | Haridas et al. (2013) |
| *Sporothrix schenckii* | SsEM7 | GCA_002837075.1 | 181 |  |
| *Sporothrix schenckii* | 1099-18 | GCA_000961545.1 | n.a. | Fijarczyk et al. (2022) |
| *Sporothrix* sp. CBS 140.51 | SP_CBS140.51 | GCA_029298735.1 | 797 |  |
| *Sporothrix stenoceras* | CMW 5347 | GCA_042257725.1 | 139 | Fijarczyk et al. (2022) |
| *Sporothrix stenoceras* | CMW 5344 | GCA_042257735.1 | 155 |  |
| *Sporothrix thermara* |  | GCA_963921895.1 | n.a. | Fijarczyk et al. (2022) |
| *Sporothrix variecibatus* | CBS 121960 | GCA_016097105.2 | n.a. |  |
| ***Wilhelmdebeerea oxyuri*** | **CCF 3802** | ERZ28561635 | 535 | Fijarczyk et al. (2022) |

**References**

Aylward J, Wilson AM, Visagie CM, Spraker J, Barnes I, Buitendag C, Ceriani C, Del Mar Angel L, Du Plessis D, Fuchs T (2024) IMA Genome–F19: A genome assembly and annotation guide to empower mycologists, including annotated draft genome sequences of Ceratocystis pirilliformis, Diaporthe australafricana, Fusarium ophioides, Paecilomyces lecythidis, and Sporothrix stenoceras. IMA fungus 15: 12. doi:10.1186/s43008-024-00142-z.

DiGuistini S, Wang Y, Liao NY, Taylor G, Tanguay P, Feau N, Henrissat B, Chan SK, Hesse-Orce U, Alamouti SM, Tsui CK, Docking RT, Levasseur A, Haridas S, Robertson G, Birol I, Holt RA, Marra MA, Hamelin RC, Hirst M, Jones SJ, Bohlmann J, Breuil C (2011) Genome and transcriptome analyses of the mountain pine beetle-fungal symbiont Grosmannia clavigera, a lodgepole pine pathogen. Proc Natl Acad Sci U S A 108: 2504-2509. doi:10.1073/pnas.1011289108.

Du W, Giosa D, Wei J, Giuffrè L, Shi G, El Aamri L, D’Alessandro E, Hafidi M, de Hoog S, Romeo O (2022) Long-read PacBio genome sequencing of four environmental saprophytic Sporothrix species spanning the pathogenic clade. BMC genomics 23: 506. doi:10.1186/s12864-022-08736-w.

Fijarczyk A, Hessenauer P, Hamelin RC, Landry CR (2022) Lifestyles shape genome size and gene content in fungal pathogens. Biorxiv: 2022.2008. 2024.505148. doi:10.1101/2022.08.24.505148.

Gomez OM, Alvarez LC, Muñoz JF, Misas E, Gallo JE, Jimenez MdP, Arango M, McEwen JG, Hernandez O, Clay OK (2018) Draft genome sequences of two Sporothrix schenckii clinical isolates associated with human sporotrichosis in Colombia. Genome announcements 6: 10.1128/genomea. 00495-00418. doi:10.1128/genomeA.00495-18.

Haridas S, Wang Y, Lim L, Massoumi Alamouti S, Jackman S, Docking R, Robertson G, Birol I, Bohlmann J, Breuil C (2013) The genome and transcriptome of the pine saprophyte Ophiostoma piceae, and a comparison with the bark beetle-associated pine pathogen Grosmannia clavigera. BMC genomics 14: 373. doi:10.1186/1471-2164-14-373.

Huang M, Ma Z, Zhou X (2020) Comparative genomic data provide new insight on the evolution of pathogenicity in Sporothrix species. Frontiers in Microbiology 11: 565439. doi:10.3389/fmicb.2020.565439.

Jeon J, Kim K-T, Song H, Lee G-W, Cheong K, Kim H, Choi G, Lee Y-H, Stewart JE, Klopfenstein NB (2017) Draft genome sequence of the fungus associated with oak wilt mortality in South Korea, Raffaelea quercus-mongolicae KACC44405. Genome announcements 5: 10.1128/genomea. 00797-00717.

Liu F, Chen S, Ferreira MA, Chang R, Sayari M, Kanzi AM, Wingfield BD, Wingfield MJ, Pizarro D, Crespo A (2019) Draft genome sequences of five Calonectria species from Eucalyptus plantations in China, Celoporthe dispersa, Sporothrix phasma and Alectoria sarmentosa. IMA fungus 10: 22. doi:10.1186/s43008-019-0023-5.

Nel WJ, De Beer ZW, Wingfield MJ, Poulsen M, Aanen DK, Wingfield BD, Duong TA (2021) Phylogenetic and phylogenomic analyses reveal two new genera and three new species of ophiostomatalean fungi from termite fungus combs. Mycologia 113: 1199-1217. doi:10.1080/00275514.2021.1950455

Teixeira MM, de Almeida LG, Kubitschek-Barreira P, Alves FL, Kioshima ÉS, Abadio AK, Fernandes L, Derengowski LS, Ferreira KS, Souza RC (2014) Comparative genomics of the major fungal agents of human and animal Sporotrichosis: Sporothrix schenckii and Sporothrix brasiliensis. BMC genomics 15: 943. doi:10.1186/1471-2164-15-943.

Trollip C, Carnegie AJ, Dinh Q, Kaur J, Smith D, Mann R, Rodoni B, Edwards J (2021) Ophiostomatoid fungi associated with pine bark beetles and infested pines in south-eastern Australia, including Graphilbum ipis-grandicollis sp. nov. IMA fungus 12: 24. doi:10.1186/s43008-021-00076-w.

Trollip C, Carnegie AJ, Rodoni B, Edwards J (2022) Draft genome sequences for three Ophiostoma species acquired during revisions of Australian plant pathogen reference collections. Microbiology resource announcements 11: e00175-00122. doi:10.1128/mra.00175-22.

van der Nest MA, Beirn LA, Crouch JA, Demers JE, De Beer ZW, De Vos L, Gordon TR, Moncalvo J-M, Naidoo K, Sanchez-Ramirez S (2014) Draft genomes of Amanita jacksonii, Ceratocystis albifundus, Fusarium circinatum, Huntiella omanensis, Leptographium procerum, Rutstroemia sydowiana, and Sclerotinia echinophila. IMA fungus 5: 472-486. doi:10.5598/imafungus.2014.05.02.11.

Vanderpool D, Bracewell RR, McCutcheon JP (2018) Know your farmer: ancient origins and multiple independent domestications of ambrosia beetle fungal cultivars. Molecular Ecology 27: 2077-2094.

Wingfield BD, Ades PK, Al-Naemi FA, Beirn LA, Bihon W, Crouch JA, De Beer ZW, De Vos L, Duong TA, Fields CJ (2015a) Draft genome sequences of Chrysoporthe austroafricana, Diplodia scrobiculata, Fusarium nygamai, Leptographium lundbergii, Limonomyces culmigenus, Stagonosporopsis tanaceti, and Thielaviopsis punctulata. IMA fungus 6: 233-248. doi:10.5598/imafungus.2015.06.01.15.

Wingfield BD, Ambler JM, Coetzee MP, De Beer ZW, Duong TA, Joubert F, Hammerbacher A, McTaggart AR, Naidoo K, Nguyen HD (2016a) IMA Genome-F 6: Draft genome sequences of Armillaria fuscipes, Ceratocystiopsis minuta, Ceratocystis adiposa, Endoconidiophora laricicola, E. polonica and Penicillium freii DAOMC 242723. IMA fungus 7: 217-227. doi:10.5598/imafungus.2016.07.01.11.

Wingfield BD, Barnes I, De Beer ZW, De Vos L, Duong TA, Kanzi AM, Naidoo K, Nguyen HD, Santana QC, Sayari M (2015b) Draft genome sequences of Ceratocystis eucalypticola, Chrysoporthe cubensis, C. deuterocubensis, Davidsoniella virescens, Fusarium temperatum, Graphilbum fragrans, Penicillium nordicum, and Thielaviopsis musarum. IMA fungus 6: 493-506. doi:10.5598/imafungus.2015.06.02.13.

Wingfield BD, Berger DK, Steenkamp ET, Lim H-J, Duong TA, Bluhm BH, De Beer ZW, De Vos L, Fourie G, Naidoo K (2017) Draft genome of Cercospora zeina, Fusarium pininemorale, Hawksworthiomyces lignivorus, Huntiella decipiens and Ophiostoma ips. IMA fungus 8: 385-396. doi:10.5598/imafungus.2017.08.02.10.

Wingfield BD, Duong TA, Hammerbacher A, van der Nest MA, Wilson A, Chang R, Wilhelm de Beer Z, Steenkamp ET, Wilken PM, Naidoo K (2016b) Draft genome sequences for Ceratocystis fagacearum, C. harringtonii, Grosmannia penicillata, and Huntiella bhutanensis. IMA fungus 7: 317-323. doi:10.5598/imafungus.2016.07.02.11.

Zhang Y, Zhang J, Vanderpool D, Smith JA, Rollins JA (2020) Genomic and transcriptomic insights into Raffaelea lauricola pathogenesis. BMC genomics 21: 570. doi:10.1186/s12864-020-06988-y.
