# Supplementary material for: The ambrosial mycobiota of Treptoplatypus oxyurus (Coleoptera, Platypodidae): a unique island of fungal diversity revealing Wilhelmdebeerea oxyuri gen. et sp. nov. (Ophiostomatales), and two new yeast species Blastobotrys sasensis sp. nov., and Sugiyamaella casensis sp. nov. (Dipodascales)
Source: IMA Fungus. 2026 Feb 16;17:e177075. doi: 10.3897/imafungus.17.177075 (PMC12930180; doi:10.3897/imafungus.17.177075)
Supplement: Supplementary material 3 — DNA sequences (ITS, LSU rDNA) used in the molecular phylogenetic analysis of Blastobotrys [file imafungus-17-e177075-s003.docx]

**Supplementary material 3.** DNA sequences used in the molecular phylogenetic analysis of *Blastobotrys*.

| **Taxon name** | **Strains** | **Location** | **Source** | **Sequence Accession no.** | | **Reference** |
| --- | --- | --- | --- | --- | --- | --- |
|  |  |  |  | **ITS** | **LSU** |  |
| *Blastobotrys adeninivorans* | CBS 8244 = NRRL Y-17692 = IPO 10858 = IGC 4638 = PYCC 4638 (ex-type) | The Netherlands | Soil | KY101746 | DQ442697 | (Kurtzman and Robnett 2007; Vu et al. 2016) |
| *Blastobotrys allociferrii* | CBS 5166 = NRRC 10194 = IPO 10194 (ex-type) | Germany | Human | LC158134 | LC158143 | unpublished |
| *Blastobotrys americanus* | CBS 10337 = NRRL Y-6844 = LRB 70B3 (ex-type) | USA: Kansas | Unknown | KY101748 | DQ442699 | (Kurtzman and Robnett 2007; Vu et al. 2016) |
| *Blastobotrys arbustula* | CBS 227.83 = NRRL Y-17585 (ex-type) | Finland | Indoor air | OL772655 | DQ442689 | (Kurtzman and Robnett 2007; Visagie et al. 2023) |
| *Blastobotrys aristatus* | CBS 521.75 = NRRL Y-17579 = ATCC 34215 = CCM F-410 = HPM 2672 = UAMH 4665 (ex-type) | Czech Republic | Mouldy plaster | OL772656 | DQ442686 | (Kurtzman and Robnett 2007; Visagie et al. 2023) |
| *Blastobotrys attinorum* | CBS 9734 = NRRL Y-27639 = UNESP-S156 (ex-type) | Brazil: Sao Paulo | Fungal garden of nests of the leaf-cutting ant (*Atta* *sexdens*) | KY101749 | AY442294 | (Vu et al. 2016) |
| *Blastobotrys baotianmanensis* | CBS 16024 = CICC 33083 (ex-type) | China: Henan Province, Baotianman Nature Reserve | Gut of ground beetle (*Pterostichus* *gebleri*) | KU128710 | KU128725 | (Chai et al. 2020) |
| *Blastobotrys bombycis* | CBS 15274 (ex-type) | India: Dharwad | Silkworm (*Bombyx* *mori*) | OL772657 | OL772653 | (Visagie et al. 2023) |
| *Blastobotrys buckinghamii* | CBS 13900 = NRRL Y-63727 = yHAB 196 (ex-type) | USA: Michigan, Taquamenon Falls State Park | Mushroom associated with American beech (*Fagus* *grandifolia*) | OL772658 | OL772654 | (Visagie et al. 2023) |
| *Blastobotrys capitulata* | CBS 287.82 = NRRL Y-17573 (ex-type) | South Africa | Flower, decaying tissue of candelabra tree (*Euphorbia* *ingens*) | OL772659 | DQ442688 | (Kurtzman and Robnett 2007; Visagie et al. 2023) |
| *Blastobotrys chiropterorum* | CBS 6064 = NRRL Y-17071 (ex-type) | Columbia | Liver of bat (*Mormoops* *megalophylla*) | KY101750 | DQ442682 | (Kurtzman and Robnett 2007; Vu et al. 2016) |
| *Blastobotrys davincii* | CBS 16861 = DAOMC 251467 = CMW 56638 = CN 00263 (ex-type) | Canada: Ontario, Stittsville | House dust | MW367648 | MW367634 | (Visagie et al. 2023) |
| *Blastobotrys elegans* | CBS 530.83A = NRRL Y-17572 (ex-type) | Finland | Indoor air | OL772660 | DQ442687 | (Kurtzman and Robnett 2007; Visagie et al. 2023) |
| *Blastobotrys farinosus* | CBS 140.71 = NRRL Y-17593 = IGC 4592 = ICM 2935 (ex-type) | The Netherlands | *Hirneola auricula-judae* | OL772661 | DQ442685 | (Kurtzman and Robnett 2007; Visagie et al. 2023) |
| *Blastobotrys fungorum* | CBS 259.70 = CMW 17165 = UAMH 3678 (ex-type) | Germany | Old *Fomes fomentarius* basidiome | KX590837 | KX590883 | (de Beer et al. 2016) |
| *Blastobotrys guizhouensis* | CGMCC 2.7784 | China | fermented food | PQ373576 | PP192707 | (Hu et al. 2025) |
| *Blastobotrys illinoisensis* | CBS 10339 = NRRL YB-1343 (ex-type) | USA: Illinois, Marion, Wohlwend farm | Tree | KY101751 | DQ442696 | (Kurtzman and Robnett 2007; Vu et al. 2016) |
| *Blastobotrys indianensis* | CBS 9600 = NRRL YB-1950 (ex-type) | USA: Indiana, Spencer, McCormick’s Creek State Park | White fungus associated with pine | KY101752 | DQ442692 | (Kurtzman and Robnett 2007; Vu et al. 2016) |
| *Blastobotrys malaysiensis* | CBS 10336 = NRRL Y-6417 = EMMONS 53,539A (ex-type) | Malaysia | Cave soil | KY101753 | DQ442695 | (Kurtzman and Robnett 2007; Vu et al. 2016) |
| *Blastobotrys meliponae* | CBS 14100 = URM7224 (ex-type) | Brazil: Pernambuco, Recife | Honey | KT448719 | KR779217 | (Crous et al. 2016) |
| *Blastobotrys mokoenaii* | CBS 8435 = NRRL Y-27120 (ex-type) | South Africa | Soil | KY101754 | DQ442694 | (Kurtzman and Robnett 2007; Vu et al. 2016) |
| *Blastobotrys mucifer* | CBS 7409 = CCY 29–170-1 = IFO 10918 (ex-type) | Brazil: Manaus | Liver of toad (*Rhinella granulosa*) | KY102217 | KY106587 | (Vu et al. 2016) |
| *Blastobotrys muscicola* | CBS 10338 = NRRL Y-7993 (ex-type) | USA: Louisiana | Moss on fallen log | KY101755 | DQ442680 | (Kurtzman and Robnett 2007 ; Vu et al. 2016) |
| *Blastobotrys navarrensis* | CBS 139.77 = ATCC 36953 = IJFM 2642 = UAMH 4664 (ex-type) | Spain: Pamplona | Black pepper (*Piper nigrum*) | OK623478 | OK623486 | (Palma et al. 2022) |
| *Blastobotrys nigripullensis* | CBS 17880 = DTO 455-G9 | The Netherlands | Oak wood from an ancient Roman ship | OQ876815 | OQ876820 | (Visagie et al. 2024) |
| *Blastobotrys nigripullensis* | CBS 17879 = DTO 457-E3 (ex-type) | The Netherlands | Oak wood from an ancient Roman ship | OQ876814 | OQ876819 | (Visagie et al. 2024) |
| *Blastobotrys nivea* | CBS 163.67 = NRRL Y-17581 = ATCC 18420 = HPM 26 = UAMH 4663 = MUCL 6078 (ex-type) | Germany | Municipal compost | OL772662 | DQ442690 | (Kurtzman and Robnett 2007; Visagie et al. 2023) |
| *Blastobotrys parvus* | CBS 6147 = NRRL Y-10004 (ex-type) | Antarctic Ocean | Seawater | KY101757 | DQ442693 | (Kurtzman and Robnett 2007) |
| *Blastobotrys peoriensis* | CBS 10340 = NRRL YB-2290 (ex-type) | USA: Illinois, Peoria | Unknown | KY101758 | DQ442700 | (Kurtzman and Robnett 2007; Vu et al. 2016) |
| *Blastobotrys persicus* | CBS 14259 = IBRC-M30238 (ex-type) | Iran: Ilam | Soil | OL772663 | KU659141 | (Visagie et al. 2023) |
| *Blastobotrys proliferans* | CBS 522.75 = NRRL Y-17577 = ATCC 34216 = CCM F-493 = HPM 2673 = UAMH 4666 (ex-type) | Brazil | Mite-infested nut (*Bertholletia* *excelsa*) | EU343812 | DQ442684 | \| (Kurtzman and Robnett 2007 ; Vu et al. 2016) \| \| --- \| |
| *Blastobotrys raffinosifermentans* | CBS 6800 = NRRL Y-27150 (ex-type) | Unknown | Unknown | KY101759 | DQ442698 | (Kurtzman and Robnett 2007; Vu et al. 2016) |
| *Blastobotrys robertii* | CBS 10106 = NRRL Y-27775 (ex-type) | The Netherlands | Rotten pine wood (*Pinus sylvestris*) | KY101760 | DQ839395 | (Middelhoven et al. 2000; Vu et al. 2016) |
| *Blastobotrys serpentis* | CBS 10541 = NRRL Y-48249 = MTCC 8332 = W113A = YS W113A (ex-type) | India: Hyderabad City | Trinket snake gut | KY101761 | AM410667 | (Bhadra et al. 2008; Vu et al. 2016 ) |
| ***Blastopotrys sasensis*** | **CCF 6841** | Slovakia | *Treptoplatypus* *oxyurus* | PX523827 | PX591253 | This study |
| *Blastobotrys terrestris* | CBS 7376 = NRRL Y-17704 = CSIR Y914 = IFO 10859 = IGC 5133 = PYCC 5133 (ex-type) | South Africa: Barberton | Soil | KY101762 | DQ442683 | (Vu et al. 2016) |
| *Blastobotrys vanleenenianus* | CBS 14902 (ex-type) | The Netherlands | Soil | MG986487 | MG986492 | (Groenewald et al. 2018) |
| *Blastobotrys xishuangbannaensis* | CBS 16044 = CICC 33360 (ex-type) | China: Yunnan Province, Jinghong | Rotting wood | MK682811 | MK682809 | (Chai et al. 2020) |
| *Trichomonascus apis* | CBS 10922 = NRRL Y-48475 = NCAIM Y01848 (ex-type) | Hungary | Mouldy honeycomb | KY105699 | EU790643 | (Peter et al. 2009; Vu et al. 2016 ) |
| *Trichomonascus ciferrii* | CBS 5295 = NRRL Y-10943 = ATCC 58443 = CCRC 21427 = Goto TH-26 = IFO 1854 = IGC 4164 = IMI 34464 | The Netherlands | Pig | AY493435 | DQ442681 | (Pryce et al. 2003; Kurtzman and Robnett 2007) |
| *Trichomonascus petasosporus* | CBS 9602 = NRRL Y-B2092 (ex-type) | USA: Missouri, Salem | White Oak (*Quercus* sp) | KY105704 | DQ442691 | (Kurtzman and Robnett 2007 ; Vu et al. 2016) |
| *Wickerhamiella domercqiae* | CBS 4351 = NRRL Y-6692 (ex-type) | South Africa | Wine vat | DQ911463 | DQ438240 | (Kurtzman 2007; Kurtzman and Robnett 2007 ) |

**References**

Bhadra B, Singh PK, Rao RS, Shivaji S (2008) Blastobotrys serpentis sp. nov., isolated from the intestine of a Trinket snake (Elaphe sp., Colubridae). FEMS yeast research 8: 492-498.

Crous PW, Wingfield MJ, Richardson DM, Le Roux JJ, Strasberg D, Edwards J, Roets F, Hubka V, Taylor PWJ, Heykoop M, Martin MP, Moreno G, Sutton DA, Wiederhold NP, Barnes CW, Carlavilla JR, Gene J, Giraldo A, Guarnaccia V, Guarro J, Hernandez-Restrepo M, Kolarik M, Manjon JL, Pascoe IG, Popov ES, Sandoval-Denis M, Woudenberg JHC, Acharya K, Alexandrova AV, Alvarado P, Barbosa RN, Baseia IG, Blanchette RA, Boekhout T, Burgess TI, Cano-Lira JF, Cmokova A, Dimitrov RA, Dyakov MY, Duenas M, Dutta AK, Esteve-Raventos F, Fedosova AG, Fournier J, Gamboa P, Gouliamova DE, Grebenc T, Groenewald M, Hanse B, Hardy G, Held BW, Jurjevic Z, Kaewgrajang T, Latha KPD, Lombard L, Luangsa-ard JJ, Lyskova P, Mallatova N, Manimohan P, Miller AN, Mirabolfathy M, Morozova OV, Obodai M, Oliveira NT, Ordonez ME, Otto EC, Paloi S, Peterson SW, Phosri C, Roux J, Salazar WA, Sanchez A, Sarria GA, Shin HD, Silva BDB, Silva GA, Smith MT, Souza-Motta CM, Stchigel AM, Stoilova-Disheva MM, Sulzbacher MA, Telleria MT, Toapanta C, Traba JM, Valenzuela-Lopez N, Watling R, Groenewald JZ (2016) Fungal Planet description sheets: 400-468. Persoonia 36: 316-458. doi:10.3767/003158516x692185.

de Beer ZW, Duong T, Wingfield M (2016) The divorce of *Sporothrix* and *Ophiostoma*: solution to a problematic relationship. Studies in mycology 83: 165-191.

Groenewald M, Lombard L, de Vries M, Lopez AG, Smith M, Crous PW (2018) Diversity of yeast species from Dutch garden soil and the description of six novel Ascomycetes. FEMS yeast research 18: foy076.

Hu S, Zhu Q-Y, Zhu H-Y, Liu J-Y, Shi Y, Qiu Y-J, Wen Z, Li A-H, Han P-J, Bai F-Y (2025) Yeast diversity in traditional fermented foods of ethnic minorities in China, with the descriptions of four new yeast species. IMA fungus 16: e146163. doi:10.3897/imafungus.16.146163.

Chai C-Y, Jia R-R, Chen C-Y, Hui F-L (2020) *Blastobotrys baotianmanensis* sp. nov. and *Blastobotrys xishuangbannaensis* f.a., sp. nov., two novel yeast species associated with insects and rotting wood. International Journal of Systematic and Evolutionary Microbiology 70: 4217-4223. doi:10.1099/ijsem.0.004275.

Kurtzman CP (2007) New anamorphic yeast species: *Candida infanticola* sp. nov., *Candida polysorbophila* sp. nov., *Candida transvaalensis* sp. nov. and *Trigonopsis californica* sp. nov. Antonie van Leeuwenhoek 92: 221-231.

Kurtzman CP, Robnett CJ (2007) Multigene phylogenetic analysis of the *Trichomonascus*, *Wickerhamiella* and *Zygoascus* yeast clades, and the proposal of *Sugiyamaella* gen. nov. and 14 new species combinations. FEMS yeast research 7: 141-151. doi:10.1111/j.1567-1364.2006.00157.x.

Middelhoven WJ, Guého E, De Hoog GS (2000) Phylogenetic position and physiology of *Cerinosterus cyanescens*. Antonie van Leeuwenhoek, International Journal of General and Molecular Microbiology 77: 313-320.

Palma M, Vieira E, Pataco M, Sá-Correia I (2022) Characterization of a new *Blastobotrys navarrensis* strain indicates that it is not a later synonym of Blastobotrys proliferans. International journal of systematic and evolutionary microbiology 72: 005388.

Peter G, Tornai-Lehoczki J, Dlauchy D (2009) *Trichomonascus* *apis* sp. nov., a heterothallic yeast species from honeycomb. International journal of systematic and evolutionary microbiology 59: 1550-1554.

Pryce T, Palladino S, Kay I, Coombs G (2003) Rapid identification of fungi by sequencing the ITS1 and ITS2 regions using an automated capillary electrophoresis system. Medical mycology 41: 369-381.

Visagie CM, Boekhout T, Theelen B, Dijksterhuis J, Yilmaz N, Seifert KA (2023) Da Vinci's yeast: *Blastobotrys davincii* f.a., sp. nov. Yeast 40: 7-31. doi:10.1002/yea.3816.

Visagie CM, Meijer M, Kraak B, Groenewald M, Houbraken J, Theelen B, Vorst Y, Boekhout T (2024) *Blastobotrys nigripullensis*, a new yeast species isolated from a fungal outbreak on an ancient Roman shipwreck in the Netherlands. Antonie Van Leeuwenhoek 117: 22. doi:10.1007/s10482-023-01898-x.

Vu D, Groenewald M, Szöke S, Cardinali G, Eberhardt U, Stielow B, De Vries M, Verkleij G, Crous P, Boekhout T (2016) DNA barcoding analysis of more than 9 000 yeast isolates contributes to quantitative thresholds for yeast species and genera delimitation. Studies in mycology 85: 91-105.
