# Supplementary material for: The ambrosial mycobiota of Treptoplatypus oxyurus (Coleoptera, Platypodidae): a unique island of fungal diversity revealing Wilhelmdebeerea oxyuri gen. et sp. nov. (Ophiostomatales), and two new yeast species Blastobotrys sasensis sp. nov., and Sugiyamaella casensis sp. nov. (Dipodascales)
Source: IMA Fungus. 2026 Feb 16;17:e177075. doi: 10.3897/imafungus.17.177075 (PMC12930180; doi:10.3897/imafungus.17.177075)
Supplement: Supplementary material 4 — DNA sequences (ITS, LSU rDNA) used in the molecular phylogenetic analysis of Sugiyamaella [file imafungus-17-e177075-s004.docx]

**Supplementary table 4.** DNA sequences used in the molecular phylogenetic analysis of *Sugiyamaella*.

| **Species** | **Strain** | **Locality** | **Isolated from** | **Sequence Accession no.** | | **Reference** |
| --- | --- | --- | --- | --- | --- | --- |
|  |  |  |  | **ITS** | **LSU** |  |
| *Blastobotrys indianensis* | CBS 9600ᵀ | USA | white fungus | NR_153638 | NG_055333 | (Kurtzman and Robnett 2007; Vu et al. 2016) |
| *Diddensiella caesifluorescens* | CBS 12613ᵀ | Hungary | rotten wood | JF895509 | GU195654 | (Péter et al. 2012) |
| *D. sanijacobensis* | CBS 8183ᵀ | USA | fallen trunk | NR_151808 | NG_058985 | (Vu et al. 2016) |
| *D. transvaalensis* | CBS 6663ᵀ | South Africa | forest litter | - | DQ442702 | (Kurtzman and Robnett 2007) |
| *D. luoyangensis* | NYNU 201062ᵀ | China | rotten wood | MW374289 | MW362346 | (Chai et al. 2022) |
| *D. luoyangensis* | NYNU 201074 | China | rotten wood | MW374461 | MW374460 | (Chai et al. 2022) |
| *Middelbovenomyces petrohuensis* | CBS 8173ᵀ | Chile | rotten trunk | NR_156314 | NG_055211 | (Péter et al. 2012; Vu et al. 2016) |
| *Middelbovenomyces tepae* | CBS 5115ᵀ | Chile | decaying tepa tree | NR_154200 | NG_055181 | (Péter et al. 2012; Vu et al. 2016) |
| *Spencermartinsiella cellulosicola* | CBS 11952ᵀ | China | rotten wood | NR_151783 | NG_055207 | (Péter et al. 2012; Vu et al. 2016) |
| *Sp. europaea* | CBS 11730ᵀ | Hungary | rotten wood | NR_111481 | NG_042528 | (Schoch et al. 2014) |
| *Sp. ligniputridi* | CBS 12585ᵀ | Hungary | rotten wood | NR_155842 | NG_055382 | (Péter et al. 2012; Vu et al. 2016) |
| *Sp. silnicola* | CBS 11952ᵀ | Brazil | rotten wood | KT222943 | KC906243 | (Morais et al. 2013) |
| *Sugiyamaella amazoniana* | CBS 18112 | Brazil | beetle gut | MZ254647 | MZ261923 |  |
| *S. americana* | CBS 10352ᵀ | USA | frass | NR_137759 | DQ438193 | (Kurtzman and Robnett 2007; Houseknecht et al. 2011) |
| *Su. ayubii* | CBS 14108ᵀ | Brazil | rotten wood | NR_155796 | KR184132 | (Vu et al. 2016; Sena et al. 2017) |
| *Su. bahiana* | CBS 13474ᵀ | Brazil | rotten wood | NR_155810 | KC959941 | (Vu et al. 2016; Sena et al. 2017) |
| *Su. bielyi* | CBS 18148 | Brazil | beetle gut | MZ261922 | MZ254646.2 | (Sena et al. 2017; Souza et al. 2023) |
| *Su. bonitensis* | CBS 14270ᵀ | Brazil | rotten wood | NR_155798 | KT006004 | (Vu et al. 2016; Sena et al. 2017) |
| *Su. boreocarolinensis* | NRRL YB-1835ᵀ | USA | frass | NR_165963 | DQ438221 | (Kurtzman and Robnett 2007; Vu et al. 2016) |
| *Su. bullrunensis* | CBS 11840ᵀ | USA | insect | NR_111543 | HM208601 | (Houseknecht et al. 2011) |
| ***Su. casensis*** | **CCF 6842^T^** | Slovakia | ambrosia beetle | PX523828 | PX591254 | This study |
| *Su. castrensis* | NRRL Y-17329ᵀ | Chile | rotten wood | NR_111229 | DQ438195 | (Kurtzman and Robnett 2007; Schoch et al. 2014) |
| *Su. carassensis* | CBS 14107ᵀ | Brazil | rotten wood | NR_155808 | KX550111 | (Vu et al. 2016; Sena et al. 2017) |
| *Su. chilensis* | CBS 8168ᵀ | Chile | rotten wood | DQ911454 | DQ438217 | (Kurtzman and Robnett 2007) |
| *Su. chuxiongensis* | NYNU 181038ᵀ | China | rotten wood | MK682800 | MK682795 | (Shi et al. 2021) |
| *Su. cylindrica* | NYNU 201067ᵀ | China | rotten wood | MW368732 | MW368731 | (Chai et al. 2022) |
| *Su. cylindrica* | NYNU 201034 | China | rotten wood | OM501585 | OM501589 | (Chai et al. 2022) |
| *Su. floridensis* | NRRL YB-3827ᵀ | USA | frass | NR_111230 | DQ438222 | (Kurtzman and Robnett 2007; Schoch et al. 2014) |
| *Su. gynbergii* | NRRL Y-27117ᵀ | Chile | insect | KY102116 | DQ438199 | (Kurtzman and Robnett 2007; Vu et al. 2016) |
| *Su. japonica* | CBS 10554ᵀ | Japan | frass | NR_111239 | DQ438202 | (Kurtzman and Robnett 2007; Schoch et al. 2014) |
| *Su. ligni* | CBS 13482ᵀ | Brazil | rotten wood | KX550112 | KX550112 | (Sena et al. 2017) |
| *Su. lignohabitans* | NRRL YB-1473ᵀ | USA | decayed log | NR_119622 | DQ438198 | (Kurtzman and Robnett 2007; Schoch et al. 2014) |
| *Su. marionensis* | NRRL YB-1336ᵀ | USA | decayed log | NR_111237 | DQ438197 | (Kurtzman and Robnett 2007; Schoch et al. 2014) |
| *Su. marilandica* | NRRL YB-1847ᵀ | USA | frass | NR_165965 | DQ438219 | (Kurtzman and Robnett 2007; Vu et al. 2016) |
| *Su. mastotermitis* | CBS 14182ᵀ | Berlin | termite | NR_156606 | KU883286 | (Handel et al. 2016; Vu et al. 2016) |
| *Su. neomexicana* | CBS 10349ᵀ | USA | frass | NR_165966 | DQ438201 | (Kurtzman and Robnett 2007; Vu et al. 2016) |
| *Su. novakii* | NRRL Y-27346ᵀ | Hungary | rotten wood | NR_111235 | DQ438196 | (Kurtzman and Robnett 2007; Schoch et al. 2014) |
| *Su. paludigena* | NRRL Y-12697ᵀ | Russia | pear | NR_111236 | DQ438194 | (Kurtzman and Robnett 2007; Schoch et al. 2014) |
| *Su. pinicola* | CBS 10348ᵀ | USA | frass | NR_165967 | DQ438200 | (Kurtzman and Robnett 2007) |
| *Su. qingdaonensis* | CBS 11390ᵀ | China | rotten wood | NR_151806 | FJ613527 | (Wang et al. 2010; Vu et al. 2016) |
| *Su. robnettiae* | NYNU 201066ᵀ | China | rotten wood | MW368730 | MW368701 | (Chai et al. 2022) |
| *Su. robnettiae* | NYNU 201005 | China | rotten wood | OM501584 | OM501586 | (Chai et al. 2022) |
| *Su. smithiae* | CBS 7522.2ᵀ | Brazil | soil | DQ911455 | DQ438218 | (Kurtzman and Robnett 2007) |
| *Su. trypani* | CBS 15876ᵀ | Poland | soil | MK388412 | MK387312 | (Crous et al. 2019) |
| *Su. valdiviana* | NRRL Y-7791ᵀ | Chile | rotten wood | NR_111544 | DQ438220 | (Kurtzman and Robnett 2007; Schoch et al. 2014) |
| *Su. valenteae* | CBS 14109ᵀ | Brazil | rotten wood | NR_155797 | KT005999 | (Vu et al. 2016) |
| *Su. xiaguanensis* | NYNU 161041ᵀ | China | rotten wood | KY213802 | KY213817 | (Huang et al. 2018) |
| *Su. xylanicola* | CBS 12683ᵀ | Brazil | rotten wood | KC493642 | KC493642 | (Morais et al. 2013) |
| *Su. xylolytica* | CBS 13493ᵀ | Brazil | rotten wood | KU214874 | KF889433 | (Sena et al. 2017) |
| *Su. yunnanensis* | NYNU 161059ᵀ | China | rotten wood | MT257259 | MT257257 | (Shi et al. 2021) |
| *Tortispora ganteri* | CBS 12581ᵀ | Mexico | necrotic plant tissue | NR_154483 | KC681893 | (Lachance and Kurtzman 2013; Vu et al. 2016) |
| *Tortispora caseinolytica* | CBS 7781ᵀ | USA | necrotic plant tissue | NR_154482 | NG_055343 | (Péter et al. 2012; Vu et al. 2016) |
| *Trichomonascus petasoporus* | CBS 9602ᵀ | USA | frass | NR_155940 | NG_055332 | (Péter et al. 2012; Vu et al. 2016) |
| *Zygoascus biomembranicola* | CBS 14157ᵀ | Japan | viscous gel | NR_156007 | LC060997 | (Nagatsuka et al. 2016; Vu et al. 2016) |
| *Z. bituminiphila* | CBS 8813ᵀ | Canada | tar | NR_137545 | NG_055308 | (Robert et al. 2001; Péter et al. 2012) |
| *Z. hellenicus* | CBS 5839ᵀ | Germany | mastitic bovine udder | NR_111258 | NG_055323 | (Péter et al. 2012; Schoch et al. 2014) |
| *Z. meyrae* | CBS 4099ᵀ | Greece | fermenting grape must | AY447022 | DQ438189 | (Smith et al. 2005; Kurtzman and Robnett 2007) |
| *Z. ohtamaensis* | CBS 8129ᵀ | Japan | soil | N/A | NG_066348 | (Kurtzman and Robnett 2007) |
| *Z. polysorbophila* | CBS 7317ᵀ | Japan | - | NR_160311 | NG_064312 | (Kurtzman 2007; Kurtzman and Robnett 2007) |

**References**

Crous PW, Carnegie A, Wingfield M, Sharma R, Mughini G, Noordeloos ME, Santini A, Shouche Y, Bezerra J, Dima B (2019) Fungal Planet description sheets: 868–950. Persoonia: Molecular Phylogeny and Evolution of Fungi 42: 291.

Handel S, Wang T, Yurkov AM, König H (2016) *Sugiyamaella mastotermitis* sp. nov. and *Papiliotrema odontotermitis* fa, sp. nov. from the gut of the termites *Mastotermes darwiniensis* and *Odontotermes obesus*. International Journal of Systematic and Evolutionary Microbiology 66: 4600-4608. doi:10.1099/ijsem.0.001397.

Houseknecht JL, Hart EL, Suh S-O, Zhou JJ (2011) Yeasts in the *Sugiyamaella* clade associated with wood-ingesting beetles and the proposal of *Candida bullrunensis* sp. nov. International Journal of Systematic and Evolutionary Microbiology 61: 1751-1756. doi:10.1099/ijs.0.026427-0

Huang L-N, Xi Z-W, Li Y, Hui F-L (2018) *Sugiyamaella xiaguanensis* f.a., sp. nov., a yeast species isolated from rotting wood. International Journal of Systematic and Evolutionary Microbiology 68: 3307-3310. doi:10.1099/ijsem.0.002988.

Chai C-Y, Gao W-L, Yan Z-L, Hui F-L (2022) Four new species of Trichomonascaceae (Saccharomycetales, Saccharomycetes) from Central China. MycoKeys 90: 1-18. doi:10.3897/mycokeys.90.83829.

Kurtzman CP (2007) New anamorphic yeast species: Candida infanticola sp. nov., Candida polysorbophila sp. nov., Candida transvaalensis sp. nov. and Trigonopsis californica sp. nov. Antonie Van Leeuwenhoek 92: 221-231.

Kurtzman CP, Robnett CJ (2007) Multigene phylogenetic analysis of the *Trichomonascus*, *Wickerhamiella* and *Zygoascus* yeast clades, and the proposal of *Sugiyamaella* gen. nov. and 14 new species combinations. FEMS yeast research 7: 141-151. doi:10.1111/j.1567-1364.2006.00157.x.

Lachance M-A, Kurtzman C (2013) The yeast genus Tortispora gen. nov., description of Tortispora ganteri sp. nov., Tortispora mauiana fa, sp. nov., Tortispora agaves fa, sp. nov., Tortispora sangerardonensis fa, sp. nov., Tortispora cuajiniquilana fa, sp. nov., Tortispora starmeri fa, sp. nov. and Tortispora phaffii fa, sp. nov., reassignment of Candida caseinolytica to Tortispora caseinolytica fa, comb. nov., emendation of Botryozyma, and assignment of Botryozyma, Tortispora gen. nov. and Trigonopsis to the family Trigonopsidaceae fam. nov. International Journal of Systematic and Evolutionary Microbiology 63: 3104-3114.

Morais CG, Lara CA, Marques S, Fonseca C, Lachance M-A, Rosa CA (2013) *Sugiyamaella xylanicola* sp. nov., a xylan-degrading yeast species isolated from rotting wood. International Journal of Systematic and Evolutionary Microbiology 63: 2356-2360. doi:10.1099/ijs.0.050856-0.

Nagatsuka Y, Ninomiya S, Kiyuna T, Kigawa R, Sano C, Sugiyama J (2016) Yamadazyma kitorensis fa, sp. nov. and Zygoascus biomembranicola fa, sp. nov., novel yeasts from the stone chamber interior of the Kitora tumulus, and five novel combinations in Yamadazyma and Zygoascus for species of Candida. International Journal of Systematic and Evolutionary Microbiology 66: 1692-1704.

Péter G, Dlauchy D, Price NP, Kurtzman CP (2012) Diddensiella caesifluorescens gen. nov., sp. nov., a riboflavin-producing yeast species of the family Trichomonascaceae. International Journal of Systematic and Evolutionary Microbiology 62: 3081-3087. doi:10.1099/ijs.0.042895-0.

Robert V, Bonjean B, Karutz M, Paschold H, Peeters W, Wubbolts MG (2001) Candida bituminiphila, a novel anamorphic species of yeast. International Journal of Systematic and Evolutionary Microbiology 51: 2171-2176.

Sena LM, Morais CG, Lopes MR, Santos RO, Uetanabaro AP, Morais PB, Vital MJ, de Morais MA, Lachance M-A, Rosa CA (2017) d-xylose fermentation, xylitol production and xylanase activities by seven new species of *Sugiyamaella*. Antonie Van Leeuwenhoek 110: 53-67. doi:10.1007/s10482-016-0775-5.

Shi C-F, Zhang K-H, Chai C-Y, Yan Z-L, Hui F-L (2021) Diversity of the genus *Sugiyamaella* and description of two new species from rotting wood in China. MycoKeys 77: 27–39. doi:10.3897/mycokeys.77.60077.

Schoch CL, Robbertse B, Robert V, Vu D, Cardinali G, Irinyi L, Meyer W, Nilsson RH, Hughes K, Miller AN, Kirk PM, Abarenkov K, Aime MC, Ariyawansa HA, Bidartondo M, Boekhout T, Buyck B, Cai Q, Chen J, Crespo A, Crous PW, Damm U, De Beer ZW, Dentinger BTM, Divakar PK, Dueñas M, Feau N, Fliegerova K, García MA, Ge Z-W, Griffith GW, Groenewald JZ, Groenewald M, Grube M, Gryzenhout M, Gueidan C, Guo L, Hambleton S, Hamelin R, Hansen K, Hofstetter V, Hong S-B, Houbraken J, Hyde KD, Inderbitzin P, Johnston PR, Karunarathna SC, Kõljalg U, Kovács GM, Kraichak E, Krizsan K, Kurtzman CP, Larsson K-H, Leavitt S, Letcher PM, Liimatainen K, Liu J-K, Lodge DJ, Jennifer Luangsa-ard J, Lumbsch HT, Maharachchikumbura SSN, Manamgoda D, Martín MP, Minnis AM, Moncalvo J-M, Mulè G, Nakasone KK, Niskanen T, Olariaga I, Papp T, Petkovits T, Pino-Bodas R, Powell MJ, Raja HA, Redecker D, Sarmiento-Ramirez JM, Seifert KA, Shrestha B, Stenroos S, Stielow B, Suh S-O, Tanaka K, Tedersoo L, Telleria MT, Udayanga D, Untereiner WA, Diéguez Uribeondo J, Subbarao KV, Vágvölgyi C, Visagie C, Voigt K, Walker DM, Weir BS, Weiß M, Wijayawardene NN, Wingfield MJ, Xu JP, Yang ZL, Zhang N, Zhuang W-Y, Federhen S (2014) Finding needles in haystacks: linking scientific names, reference specimens and molecular data for Fungi. Database 2014: doi:10.1093/database/bau061.

Smith MT, Robert V, Poot G, Epping W, De Cock A (2005) Taxonomy and phylogeny of the ascomycetous yeast genus Zygoascus, with proposal of Zygoascus meyerae sp. nov. and related anamorphic varieties. International Journal of Systematic and Evolutionary Microbiology 55: 1353-1363.

Souza GF, Barros KO, Alvarenga FB, Santos ARO, Fonseca CR, Abegg MA, Lachance M-A, Rosa CA (2023) *Sugiyamaella bielyi* f.a., sp. nov. and *Sugiyamaella amazoniana* f.a., sp. nov., two yeast species isolated from passalid beetles and rotting wood in Amazonia. International Journal of Systematic and Evolutionary Microbiology 73: 005839. doi:10.1099/ijsem.0.005839.

Vu D, Groenewald M, Szöke S, Cardinali G, Eberhardt U, Stielow B, De Vries M, Verkleij G, Crous P, Boekhout T (2016) DNA barcoding analysis of more than 9 000 yeast isolates contributes to quantitative thresholds for yeast species and genera delimitation. Studies in Mycology 85: 91-105. doi:10.1016/j.simyco.2016.11.007.

Wang S-A, Li F-L, Bai F-Y (2010) *Candida laoshanensis* sp. nov. and *Candida qingdaonensis* sp. nov., anamorphic, ascomycetous yeast species isolated from decayed wood. International Journal of Systematic and Evolutionary Microbiology 60: 1697-1701. doi:10.1099/ijs.0.015230-0.
