# Supplementary material for: The ambrosial mycobiota of Treptoplatypus oxyurus (Coleoptera, Platypodidae): a unique island of fungal diversity revealing Wilhelmdebeerea oxyuri gen. et sp. nov. (Ophiostomatales), and two new yeast species Blastobotrys sasensis sp. nov., and Sugiyamaella casensis sp. nov. (Dipodascales)
Source: IMA Fungus. 2026 Feb 16;17:e177075. doi: 10.3897/imafungus.17.177075 (PMC12930180; doi:10.3897/imafungus.17.177075)
Supplement: Supplementary material 5 — List of whole genomic sequences used in the phylogenomic study of Saccharomycetales [file imafungus-17-e177075-s005.docx]

**Supplementary material 5.** List of whole genomic sequences used in the phylogenomic study of *Dipodascomycetes*.

| **Species** | **Assembly Accession** | **Strain** | **Number of Scaffolds** | **Reference** |
| --- | --- | --- | --- | --- |
| *[Candida] bentonensis* | GCA_030561725.1 | NRRL YB-2364 | 343 | (Opulente et al. 2023) |
| *[Candida] blankii* | GCA_024734315.1 | ABL | - | unpublished |
| *[Candida] digboiensis* | GCA_030570855.1 | CBS 9800 | 1469 | (Opulente et al. 2023) |
| *[Candida] hispaniensis* | GCA_900535975.1 | CBS 9996 | 6 | unpublished |
| *[Candida] incommunis* | GCA_003706695.3 | NRRL Y-17085 | 118 | (Shen et al. 2018) |
| *[Candida] lundiana* | GCA_030563415.1 | CBS 12271 T | 155 | (Opulente et al. 2023) |
| *[Candida] patagonica (nom. inval.)* | GCA_030580475.1 | CBS 10443 | 110 | (Opulente et al. 2023) |
| *[Candida] suthepensis* | GCA_030568895.1 | CBS 12270 T | 181 | (Opulente et al. 2023) |
| *[Candida] tunisiensis* | GCA_030407195.1 | CBS 12513 | 778 | (Opulente et al. 2023) |
| *Alloascoidea hylecoeti* | GCA_001600815.1 | JCM 7604 | 136 | unpublished |
| ***Blastobotrys sasensis*** | ERZ28669457 | CCF 6841 | 65 | This study |
| *Blastobotrys adeninivorans* | GCA_016162255.1 | TMCC 70007 | 31 | unpublished |
| *Blastobotrys allociferrii* | GCA_045529695.1 | CBS 18616 | 4 | (Deroche et al. 2025) |
| *Blastobotrys americana* | GCA_003705795.3 | NRRL Y-6844 | 77 | (Shen et al. 2018) |
| *Blastobotrys arbuscula* | GCA_030564365.1 | NRRL Y-17585 | 1150 | (Opulente et al. 2023) |
| *Blastobotrys aristatus* | GCA_946409495.1 | UCD613 | 4 | unpublished |
| *Blastobotrys attinorum* | GCA_030564265.1 | NRRL Y-27639 | 250 | (Opulente et al. 2023) |
| *Blastobotrys buckinghamii (nom. inval.)* | GCA_030579975.1 | NRRL Y-63727 | 950 | (Opulente et al. 2023) |
| *Blastobotrys capitulatus* | GCA_030564325.1 | NRRL Y-17573 | 1681 | (Opulente et al. 2023) |
| *Blastobotrys elegans* | GCA_030564235.1 | NRRL Y-17572 | 1086 | (Opulente et al. 2023) |
| *Blastobotrys chiropterorum* | GCA_030570055.1 | NRRL Y-17071 | 440 | (Opulente et al. 2023) |
| *Blastobotrys illinoisensis* | GCA_965113335.1 | NRRL YB-1343 | - | unpublished |
| *Blastobotrys indianensis* | GCA_030558855.1 | NRRL YB-1950 | 133 | (Opulente et al. 2023) |
| *Blastobotrys malaysiensis* | GCA_030558815.1 | NRRL Y-6417 | 2553 | (Opulente et al. 2023) |
| *Blastobotrys mokoenaii* | GCA_003705765.3 | NRRL Y-27120 | - | (Shen et al. 2018) |
| *Blastobotrys mucifer* | GCA_036871495.1 | 17G1312 | 8 | (Dai et al. 2024) |
| *Blastobotrys muscicola* | GCA_003705745.3 | NRRL Y-7993 | 456 | (Shen et al. 2018) |
| *Blastobotrys niveus* | GCA_030558825.1 | NRRL Y-17581 | 1459 | (Opulente et al. 2023) |
| *Blastobotrys parvus* | GCA_030564285.1 | NRRL Y-1004 | 126 | (Opulente et al. 2023) |
| *Blastobotrys peoriensis* | GCA_003705735.3 | NRRL YB-2290 | 199 | (Shen et al. 2018) |
| *Blastobotrys persicus* | GCA_030585065.1 | CBS 14259 | 442 | (Opulente et al. 2023) |
| *Blastobotrys proliferans* | GCA_003707485.3 | NRRL Y-17577 | 181 | (Shen et al. 2018) |
| *Blastobotrys raffinosifermentans* | GCA_003705705.2 | NRRL Y-27150 | 140 | (Shen et al. 2018) |
| *Blastobotrys robertii* | GCA_030564385.1 | NRRL Y-27775 | 330 | (Opulente et al. 2023) |
| *Blastobotrys serpentis* | GCA_003705695.2 | NRRL Y-48249 | 285 | (Shen et al. 2018) |
| *Blastobotrys terrestris* | GCA_030674225.1 | NRRL Y-17704 | 3343 | (Opulente et al. 2023) |
| *Botryozyma nematodophila* | GCA_030579355.1 | NRRL Y-17705 | 2720 | (Opulente et al. 2023) |
| *Crinitomyces ghanaensis* | GCA_030581755.1 | NRRL YB-1486T | 209 | (Opulente et al. 2023) |
| *Deakozyma indianensis* | GCA_003706415.3 | NRRL YB-1937 | 71 | (Shen et al. 2018) |
| *Diddensiella caesifluorescens* | GCA_030558955.1 | NRRL Y-48781 | 203 | (Opulente et al. 2023) |
| *Diddensiella santjacobensis* | GCA_030574215.1 | NRRL Y-17667 | 88 | (Opulente et al. 2023) |
| *Diddensiella transvaalensis* | GCA_030555955.1 | NRRL Y-27140 | 94 | (Opulente et al. 2023) |
| *Dipodascus aggregatus* | GCA_030564925.1 | NRRL Y-17564 | 529 | (Opulente et al. 2023) |
| *Dipodascus albidus* | GCA_043388505.1 | NRRL Y-12859 | 200 | (Opulente et al. 2023) |
| *Dipodascus australiensis* | GCA_030563945.1 | NRRL Y-17565 | 468 | (Opulente et al. 2023) |
| *Dipodascus geniculatus* | GCA_030566045.1 | NRRL Y-17628 | 148 | (Opulente et al. 2023) |
| *Dipodascus siamensis* | GCA_030568955.1 | CBS 10929 T | 1224 | (Opulente et al. 2023) |
| *Geotrichum armillariae* | GCA_030674515.1 | NRRL Y-17580 | 4702 | (Opulente et al. 2023) |
| *Geotrichum bryndzae* | GCA_025605225.1 | LMA-1146 | 486 | unpublished |
| *Geotrichum candidum* | GCA_013365045.1 | LMA-244 | 168 | (Perkins et al. 2020) |
| *Geotrichum carabidarum* | GCA_030556585.1 | NRRL Y-27727 | 810 | (Opulente et al. 2023) |
| *Geotrichum citri-aurantii* | GCA_030463015.1 | CBS 176.89T | 3083 | (Opulente et al. 2023) |
| *Geotrichum cucujoidarum* | GCA_030566025.1 | NRRL Y-27731 | 365 | (Opulente et al. 2023) |
| *Geotrichum europaeum* | GCA_030567025.1 | CBS 866.68 | 3338 | (Opulente et al. 2023) |
| *Geotrichum fermentans* | GCA_030564905.1 | NRRL Y-1492 | 820 | (Opulente et al. 2023) |
| *Geotrichum galactomycetum* | GCA_013305805.1 | LMA-1147 | 668 | (Perkins et al. 2020) |
| *Geotrichum ghanense* | GCA_022023575.1 | CBS 11010 | - | unpublished |
| *Geotrichum histeridarum* | GCA_030566085.1 | NRRL Y-27729 | 342 | (Opulente et al. 2023) |
| *Geotrichum klebahnii* | GCA_030570935.1 | NRRL Y-17568 | 373 | (Opulente et al. 2023) |
| *Geotrichum macrosporum* | GCA_030463045.1 | NRRL Y-17586 | 4621 | (Opulente et al. 2023) |
| *Geotrichum phurueaensis* | GCA_030558065.1 | CBS 11418 | 1929 | (Opulente et al. 2023) |
| *Geotrichum reessii* | GCA_013305925.1 | LMA-1148 | 753 | (Perkins et al. 2020) |
| *Geotrichum restrictum* | GCA_030563985.1 | CBS 111234 | 1458 | (Opulente et al. 2023) |
| *Groenewaldozyma auringiensis* | GCA_030574195.1 | NRRL Y-17674 | 94 | (Opulente et al. 2023) |
| *Groenewaldozyma salmanticensis* | GCA_003708315.3 | NRRL Y-17090 | 122 | (Shen et al. 2018) |
| *Groenewaldozyma tartarivorans* | GCA_030574255.1 | NRRL Y-27291T | 511 | (Opulente et al. 2023) |
| *Hyphopichia burtonii* | GCF_001661395.1 | NRRL Y-1933 | 27 | (Riley et al. 2016) |
| *Kuraishia molischiana* | GCA_024271875.1 | VH39_BB | 332 | (Cheng et al. 2023) |
| *Lipomyces kockii* | GCA_030568215.1 | NRRL Y-27505 | 681 | (Opulente et al. 2023) |
| *Magnusiomyces capitatus* | GCA_900497725.1 | - | - | (Brejová et al. 2019a) |
| *Magnusiomyces clavatus* | GCA_000817185.1 | CNRMA 12.647 | 339 | (Vaux et al. 2014) |
| *Magnusiomyces fungicola* | GCA_900654225.1 | - | - | (Brejová et al. 2019b) |
| *Magnusiomyces ingens* | GCA_900497715.1 | - | - | (Brejová et al. 2019a) |
| *Magnusiomyces magnusii* | GCA_030570155.1 | NRRL Y-17563 | 9762 | (Opulente et al. 2023) |
| *Magnusiomyces paraingens* | GCF_902498895.1 | CBS 517.90 | - | (Brejová et al. 2019a) |
| *Magnusiomyces starmeri* | GCA_030578655.1 | NRRL Y-17816 | 4323 | (Opulente et al. 2023) |
| *Magnusiomyces suaveolens* | GCA_900642975.1 | - | - | (Brejová et al. 2019b) |
| *Magnusiomyces tetraspermus* | GCA_030578715.1 | NRRL Y-7288 | 540 | (Opulente et al. 2023) |
| *Meyerozyma guilliermondii* | GCF_000149425.1 | ATCC 6260 | 9 |  |
| *Middelhovenomyces tepae* | GCA_003708105.3 | NRRL Y-17670 | 38 | (Shen et al. 2018) |
| *Nadsonia commutata* | GCA_030563525.1 | NRRL Y-7950T | 479 | (Opulente et al. 2023) |
| *Nadsonia fulvescens* var. *elongata* | GCA_001661315.1 | DSM 6958 | 20 |  |
| *Nadsonia starkeyi-henricii* | GCA_030560865.1 | NRRL YB-3963 | 331 | (Opulente et al. 2023) |
| *Nakazawaea ambrosiae* | GCA_024271865.1 | VH60_CP | 296 | (Cheng et al. 2023) |
| *Saccharomyces cerevisiae* | GCF_000146045.2 | S288C | 16 |  |
| *Saccharomycodes ludwigii* | GCF_020623625.1 | NBRC 1722 | 8 |  |
| *Spencermartinsiella cellulosicola* | GCA_040208345.1 | HNY3503 | 87 | (Barros et al. 2024) |
| *Spencermartinsiella europaea* | GCA_030579595.1 | NRRL Y-48265 | 339 | (Opulente et al. 2023) |
| *Spencermartinsiella ligniputridi* | GCA_030583385.1 | NRRL Y-48818 | 92 | (Opulente et al. 2023) |
| *Sporopachydermia lactativora* | GCA_030579575.1 | NRRL Y-11591 | 597 | (Opulente et al. 2023) |
| *Starmerella apicola* | GCA_030572115.1 | NRRL Y-2481 | 80 | (Opulente et al. 2023) |
| *Starmerella apis* | GCA_030564665.1 | NRRL Y-2482 | 87 | (Opulente et al. 2023) |
| *Starmerella asiatica* | GCA_030572815.1 | CBS 14173 | 462 | (Opulente et al. 2023) |
| *Starmerella bacillaris* | GCA_030020325.1 |  | 5 | (Opulente et al. 2023) |
| *Starmerella bombi* | GCA_030579915.1 | NRRL Y-17081 | 382 | (Opulente et al. 2023) |
| *Starmerella bombicola* | GCA_048771825.1 | SL-2025 |  | unpublished |
| *Starmerella cellae* | GCA_030579775.1 | NRRL Y-27860 | 262 | (Opulente et al. 2023) |
| *Starmerella davenportii* | GCA_030579095.1 | CBS 9069 | 188 | (Opulente et al. 2023) |
| *Starmerella etchellsii* | GCA_030490775.1 | NRRL Y-17084 | - | (Opulente et al. 2023) |
| *Starmerella floricola* | GCA_030579735.1 | NRRL Y-17676 | 224 | (Opulente et al. 2023) |
| *Starmerella floris* | GCA_030555655.1 | NRRL Y-48255 | 5488 | (Opulente et al. 2023) |
| *Starmerella geochares* | GCA_030705075.1 | NRRL Y-17073 | 192 | (Opulente et al. 2023) |
| *Starmerella gropengiesseri* | GCA_030579755.1 | NRRL Y-17142 | 343 | (Opulente et al. 2023) |
| *Starmerella khaoyaiensis* | GCA_030563285.1 | NBRC 104213, DQ400367 | 174 | (Opulente et al. 2023) |
| *Starmerella kuoi* | GCA_030579695.1 | NRRL Y-27208 | 339 | (Opulente et al. 2023) |
| *Starmerella lactis-condensi* | GCA_030568935.1 | NRRL Y-1515 | 147 | (Opulente et al. 2023) |
| *Starmerella magnoliae* | GCA_030762955.1 | MDK-2023a | - | (Opulente et al. 2023) |
| *Starmerella potacharoeniae* | GCA_030566975.1 | NBRC 106439, AB537437 | 209 | (Opulente et al. 2023) |
| *Starmerella powellii* (nom. inval.) | GCA_030568915.1 | CBS 8795T | 84 | (Opulente et al. 2023) |
| *Starmerella ratchasimensis* | GCA_030562965.1 | CBS 10611 | - | (Opulente et al. 2023) |
| *Starmerella riodocensis* | GCA_004124955.1 | NRRL Y-27859 | 1015 | (Kominek et al. 2019) |
| *Starmerella roubikii* | GCA_030584945.1 | CBS 15148 | 158 | (Opulente et al. 2023) |
| *Starmerella scarabaei* | GCA_030565305.1 | CBS 14174 | - | (Opulente et al. 2023) |
| *Starmerella sirachaensis* | GCA_030578495.1 | NBRC 108605, AB617909 | - | (Opulente et al. 2023) |
| *Starmerella sorbosivorans* | GCA_030555795.1 | NCYC 2938 | 615 | (Opulente et al. 2023) |
| *Starmerella stellata* | GCA_030674195.1 | NRRL Y-1446 | 212 | (Opulente et al. 2023) |
| *Starmerella stigmatis* (nom. inval.) | GCA_030563545.1 | CBS 11464 T | 134 | (Opulente et al. 2023) |
| *Starmerella tilneyi* (nom. inval.) | GCA_030566755.1 | CBS 8794 | 193 | (Opulente et al. 2023) |
| *Starmerella vaccinii* | GCA_004125185.1 | NRRL Y-17684 | 254 | (Kominek et al. 2019) |
| *Starmerella vitae* | GCA_030584925.1 | CBS 15147 | 199 | (Opulente et al. 2023) |
| ***Sugiyamaella casensis*** | ERZ28669458 | CCF 6842 | 133 | This study |
| *Sugiyamaella americana* | GCA_030583405.1 | NRRL YB-2067 | 505 | (Opulente et al. 2023) |
| *Sugiyamaella boreocaroliniensis* | GCA_030575195.1 | NRRL YB-1835 | 152 | (Opulente et al. 2023) |
| *Sugiyamaella castrensis* | GCA_030583505.1 | NRRL Y-17329 | 554 | (Opulente et al. 2023) |
| *Sugiyamaella floridensis* | GCA_030556845.1 | NRRL YB-3827 | 128 | (Opulente et al. 2023) |
| *Sugiyamaella grinbergsii* | GCA_030579895.1 | NRRL Y-27117 | 274 | (Opulente et al. 2023) |
| *Sugiyamaella chiloensis* | GCA_030578775.1 | NRRL Y-17643 | 171 | (Opulente et al. 2023) |
| *Sugiyamaella japonica* | GCA_030583365.1 | NRRL YB-2798 | 633 | (Opulente et al. 2023) |
| *Sugiyamaella lignohabitans* | GCF_001640025.1 | CBS 10342 | 4 | (Opulente et al. 2023) |
| *Sugiyamaella marilandica* | GCA_030563205.1 | NRRL YB-1847 | 207 | (Opulente et al. 2023) |
| *Sugiyamaella marionensis* | GCA_043388925.1 | NRRL YB-1336 | 2172 | (Opulente et al. 2023) |
| *Sugiyamaella mastotermitis* | GCA_030572735.1 | CBS 14182 | 604 | (Opulente et al. 2023) |
| *Sugiyamaella neomexicana* | GCA_030555635.1 | NRRL YB-2450 | 1473 | (Opulente et al. 2023) |
| *Sugiyamaella novakii* | GCA_030575175.1 | NRRL Y-27346 | 238 | (Opulente et al. 2023) |
| *Sugiyamaella paludigena* | GCA_030579875.1 | NRRL Y-12697 | 505 | (Opulente et al. 2023) |
| *Sugiyamaella pinicola* | GCA_030565085.1 | NRRL YB-2263 | 611 | (Opulente et al. 2023) |
| *Sugiyamaella qingdaonensis* | GCA_030569235.1 | CBS 11390 | 80 | (Opulente et al. 2023) |
| *Sugiyamaella smithiae* | GCA_030579815.1 | NRRL Y-17850 | 147 | (Opulente et al. 2023) |
| *Sugiyamaella valdiviana* | GCA_030570555.1 | NRRL Y-7791 | - | (Opulente et al. 2023) |
| *Sugiyamaella xylanicola* (nom. inval.) | GCA_001939105.2 | UFMG-CM-Y1884 | - | (Batista et al. 2017) |
| *Tardiomyces depauwii* (nom. inval.) | GCA_038086965.1 | NCPF13064 | - | (Spruijtenburg et al. 2024) |
| *Trichomonascus apis* | GCA_030557665.1 | NRRL Y-48475 | 355 | (Opulente et al. 2023) |
| *Trichomonascus ciferrii* | GCA_030573635.1 | NRRL Y-10943 | 642 | (Opulente et al. 2023) |
| *Trichomonascus petasosporus* | GCA_030580095.1 | NRRL YB-2092 | 375 | (Opulente et al. 2023) |
| *Trichomonascus vanleenenianus* | GCA_030572835.1 | CBS 14902 | 635 | (Opulente et al. 2023) |
| *Wickerhamiella allomyrinae* | GCA_030581455.1 | CBS 13167 | 412 | (Opulente et al. 2023) |
| *Wickerhamiella alocasiicola* | GCA_022577715.1 | PYCC 8427 | - | unpublished |
| *Wickerhamiella australiensis* | GCA_030579635.1 | NRRL Y-27360 | 530 | (Opulente et al. 2023) |
| *Wickerhamiella azyma* | GCA_022577855.1 | PYCC 8333 | - | unpublished |
| *Wickerhamiella azymoides* | GCA_030563245.1 | CBS 10508T | 198 | (Opulente et al. 2023) |
| *Wickerhamiella bombiphila* | GCA_030561035.1 | NRRL Y-27640 | 91 | (Opulente et al. 2023) |
| *Wickerhamiella brachini* | GCA_030565285.1 | CBS 14176 | 96 | (Opulente et al. 2023) |
| *Wickerhamiella cacticola* | GCA_030564425.1 | NRRL Y-27362 | 514 | (Opulente et al. 2023) |
| *Wickerhamiella dianesei* | GCA_022577725.1 | PYCC 8330 | - | unpublished |
| *Wickerhamiella domercqiae* | GCA_001599275.1 | JCM 9478 | 4 | (Shen et al. 2018) |
| *Wickerhamiella galacta* | GCA_030582755.1 | NRRL Y-17645 | 324 | (Opulente et al. 2023) |
| *Wickerhamiella hasegawae* | GCA_030572535.1 | JCM 12559 | 780 | (Opulente et al. 2023) |
| *Wickerhamiella infanticola* | GCA_030574435.1 | NRRL Y-17858 | - | (Opulente et al. 2023) |
| *Wickerhamiella kazuoi* | GCA_030555755.1 | JCM 12558 | 1330 | (Opulente et al. 2023) |
| *Wickerhamiella kurtzmanii* | GCA_030572965.1 | CBS 15383 | 176 | (Opulente et al. 2023) |
| *Wickerhamiella lipophila* | GCA_030582055.1 | NRRL Y-27367 | 207 | (Opulente et al. 2023) |
| *Wickerhamiella musiphila* | GCA_030558175.1 | CBS 10697 | 217 | (Opulente et al. 2023) |
| *Wickerhamiella nectarea* | GCA_022577815.1 | PYCC 8436 | - | unpublished |
| *Wickerhamiella occidentalis* | GCA_030582035.1 | NRRL Y-27364 | 165 | (Opulente et al. 2023) |
| *Wickerhamiella pararugosa* | GCA_023628975.1 | PX1910 | 31 | unpublished |
| *Wickerhamiella parazyma* | GCA_030562575.1 | NRRL Y-48669T | 222 | (Opulente et al. 2023) |
| *Wickerhamiella qilinensis* | GCA_030556915.1 | CBS 13929 | 238 | (Opulente et al. 2023) |
| *Wickerhamiella sergipiensis* | GCA_030563925.1 | CBS 9567 | 269 | (Opulente et al. 2023) |
| *Wickerhamiella shivajii* | GCA_030585105.1 | CBS 15893 | 91 | (Opulente et al. 2023) |
| *Wickerhamiella siamensis* | GCA_030571815.1 | CBS 13331 | - | (Opulente et al. 2023) |
| *Wickerhamiella slavikovae* | GCA_954870865.1 | - | - | unpublished |
| *Wickerhamiella sorbophila* | GCF_002251995.1 | DS02 | - | unpublished |
| *Wickerhamiella spandovensis* | GCA_022577695.1 | PYCC 8431 | - | unpublished |
| *Wickerhamiella vanderwaltii* | GCA_030582415.1 | NRRL Y-17671 | 182 | (Opulente et al. 2023) |
| *Wickerhamiella versatilis* | GCA_030867685.1 | HR3 | 8 | (Opulente et al. 2023) |
| *Yarrowia alimentaria* | GCA_900518985.1 | CBS 10151 | 11 | unpublished |
| *Yarrowia brassicae* | GCA_030585005.1 | CBS 15225 | 407 | (Opulente et al. 2023) |
| *Yarrowia bubula* | GCA_900519075.1 | CBS 12934 | 28 | unpublished |
| *Yarrowia deformans* | GCA_900519085.1 | CBS 2071 | 42 | unpublished |
| *Yarrowia divulgata* | GCA_900519045.1 | CBS 11013 | 19 | unpublished |
| *Yarrowia galli* | GCA_900519055.1 | CBS 9722 | 6 | unpublished |
| *Yarrowia hollandica* | GCA_900519065.1 | CBS 4855 | 18 | unpublished |
| *Yarrowia keelungensis* | GCA_001600195.1 | JCM 14894 | 41 | (Shen et al. 2018) |
| *Yarrowia lipolytica* | GCA_001761485.1 | CLIB89(W29) | 6 | (Magnan et al. 2016) |
| *Yarrowia osloensis* | GCA_900519015.1 | CBS 10146 | 37 | unpublished |
| *Yarrowia phangngaensis* | GCA_900519005.1 | CBS 10407 | 9 | unpublished |
| *Yarrowia porcina* | GCA_900519025.1 | CBS 12935 | 43 | unpublished |
| *Yarrowia yakushimensis* | GCA_900518995.1 | CBS 10253 | 7 | unpublished |
| *Zygoascus biomembranicola* | GCA_030556875.1 | CBS 14157 | 62 | (Opulente et al. 2023) |
| *Zygoascus bituminiphila* | GCA_030705125.1 | CBS 8813 | 144 | (Opulente et al. 2023) |
| *Zygoascus flipseniorum* | GCA_030565365.1 | CBS 14876 | 63 | (Opulente et al. 2023) |
| *Zygoascus hellenicus* | GCA_030564625.1 | NRRL Y-7136 | 144 | (Opulente et al. 2023) |
| *Zygoascus meyerae* | GCA_030561445.1 | NRRL Y-17319 | 39 | (Opulente et al. 2023) |
| *Zygoascus ofunaensis* | GCA_003707925.3 | NRRL Y-10998 | 166 | (Shen et al. 2018) |
| *Zygoascus polysorbophila* | GCA_030563625.1 | NRRL Y-27161T | 56 | (Opulente et al. 2023) |
| *Zygoascus tannicola* | GCA_030569095.1 | NRRL Y-17392T | 68 | (Opulente et al. 2023) |

**Reference**

Barros KO, Valério AD, Batista TM, Santos ARO, Souza GF, Alvarenga FB, Lopes MR, Morais CG, Alves C, Goes-Neto A (2024) *Spencermartinsiella nicolii* sp. nov., a potential opportunistic pathogenic yeast species isolated from rotting wood in Brazil. International journal of systematic and evolutionary microbiology 74: 006520.

Batista TM, Moreira RG, Hilário HO, Morais CG, Franco GR, Rosa LH, Rosa CA (2017) Draft genome sequence of Sugiyamaella xylanicola UFMG-CM-Y1884T, a xylan-degrading yeast species isolated from rotting wood samples in Brazil. Genomics Data 11: 120-121. doi:https://doi.org/10.1016/j.gdata.2017.01.006.

Brejová B, Lichancová H, Brázdovič F, Hegedűsová E, Forgáčová Jakúbková M, Hodorová V, Džugasová V, Baláž A, Zeiselová L, Cillingová A (2019a) Genome sequence of the opportunistic human pathogen *Magnusiomyces capitatus*. Current genetics 65: 539-560.

Brejová B, Lichancová H, Hodorová V, Neboháčová M, Tomáška Ľ, Vinař T, Nosek J (2019b) Genome sequence of an arthroconidial yeast, *Saprochaete fungicola* CBS 625.85. Microbiology resource announcements 8: 10.1128/mra. 00092-00019.

Dai R-C, Guan J, Ning Y-T, Kudinha T, Zhang W, Chen X-F, Zhang G, Xu Y-C, Xiao M (2024) Complete Genome Sequence of *Candida* *mucifera* from an Otitis Media Patient. Mycopathologia 189: 78.

Deroche L, Deffois E, Cateau E, Buyck J, Brunet K (2025) Diploid Genome Assembly of the Blastobotrys allociferrii Clinical Isolate CBS 18616. Mycopathologia 190: 76.

Cheng T, Veselská T, Křížková B, Švec K, Havlíček V, Stadler M, Kolařík M (2023) Insight into the genomes of dominant yeast symbionts of European spruce bark beetle, Ips typographus. Frontiers in Microbiology 14: 930.

Kominek J, Doering DT, Opulente DA, Shen X-X, Zhou X, DeVirgilio J, Hulfachor AB, Groenewald M, Mcgee MA, Karlen SD (2019) Eukaryotic acquisition of a bacterial operon. Cell 176: 1356-1366. e1310.

Magnan C, Yu J, Chang I, Jahn E, Kanomata Y, Wu J, Zeller M, Oakes M, Baldi P, Sandmeyer S (2016) Sequence assembly of Yarrowia lipolytica strain W29/CLIB89 shows transposable element diversity. PLoS ONE 11: e0162363.

Opulente DA, LaBella AL, Harrison M-C, Wolters JF, Liu C, Li Y, Kominek J, Steenwyk JL, Stoneman HR, VanDenAvond J (2023) Genomic and ecological factors shaping specialism and generalism across an entire subphylum. Biorxiv:

Perkins V, Vignola S, Lessard M-H, Plante P-L, Corbeil J, Dugat-Bony E, Frenette M, Labrie S (2020) Phenotypic and genetic characterization of the cheese ripening yeast *Geotrichum candidum*. Frontiers in Microbiology 11: 737.

Riley R, Haridas S, Wolfe KH, Lopes MR, Hittinger CT, Göker M, Salamov AA, Wisecaver JH, Long TM, Calvey CH (2016) Comparative genomics of biotechnologically important yeasts. Proceedings of the National Academy of Sciences 113: 9882-9887.

Shen X-X, Opulente DA, Kominek J, Zhou X, Steenwyk JL, Buh KV, Haase MA, Wisecaver JH, Wang M, Doering DT (2018) Tempo and mode of genome evolution in the budding yeast subphylum. Cell 175: 1533-1545. e1520.

Spruijtenburg B, de Souza Lima BJF, Tosar STG, Borman AM, Andersen CT, Nizamuddin S, Ahmad S, de Almeida Junior JN, Vicente VA, Nosanchuk JD, Buil JB, de Hoog S, Meijer EFJ, Meis JF, de Groot T (2024) The yeast genus Tardiomyces gen. nov. with one new species and two new combinations. Infection 52: 1799-1812. doi:10.1007/s15010-024-02229-6.

Vaux S, Criscuolo A, Desnos-Ollivier M, Diancourt L, Tarnaud C, Vandenbogaert M, Brisse S, Coignard B, Dromer F, Group GI (2014) Multicenter outbreak of infections by Saprochaete clavata, an unrecognized opportunistic fungal pathogen. MBio 5: 10.1128/mbio. 02309-02314.
