# Supplementary material for: The ambrosial mycobiota of Treptoplatypus oxyurus (Coleoptera, Platypodidae): a unique island of fungal diversity revealing Wilhelmdebeerea oxyuri gen. et sp. nov. (Ophiostomatales), and two new yeast species Blastobotrys sasensis sp. nov., and Sugiyamaella casensis sp. nov. (Dipodascales)
Source: IMA Fungus. 2026 Feb 16;17:e177075. doi: 10.3897/imafungus.17.177075 (PMC12930180; doi:10.3897/imafungus.17.177075)
Supplement: Supplementary material 12 — Geography of main mycangial symbionts, studied using GlobalFungi database [file imafungus-17-e177075-s012.docx]

**Supplementary material 12.** Geography of main mycangial symbionts, studied using GlobalFungi database. Primary data are presented for (**A**) *Wilhelmdebeerea oxyuri*, **(B)** *Candida schatavii*, **(C)** *Blastobotrys sasensis* and **(C)** *Sugiyamaella casensis.*

**Supplementary material 12 A.** Geography of *Wilhelmdebeerea oxyuri* studied using GlobalFungi. Primary data.

ITS2: CCCCCTCAGCGCGCCTCGTTGCGCCGCTGGTGTTGGGGCACCTCGAGACGCCCTCCCCCCCTGGGGGGTTGGCGGCCGAGGGCCCCCAAAGCGAGTGGCGGGCCTGGCTGGTTGGCTCCGAGCGCAGTACCGAACGCATGTTCTCCTCTCGCTCTGCAGCCCCGGCCGGCGCCCTGCCGTCAACGCGCACAGTGACGTGCAGCCTCATTTT

Hits with 100-99.5% similarity.

| id | paper | permanent_id | sample_type | latitude | longitude | continent | year_of_sampling_from | year_of_sampling_to | Biome | primers | MAT | MAP | pH | SOC | ITS_total | manipulated | abundances |
| --- | --- | --- | --- | --- | --- | --- | --- | --- | --- | --- | --- | --- | --- | --- | --- | --- | --- |
| 129 | 71 | GF05023919S | deadwood | 60.1765 | 24.6192 | Europe | 2018 | 2018 | forest | gITS7/ITS4 | 5.6 | 665 | 4.6 | 15.7 | 49921 | 0 | 234 |
| 317 | 71 | GF05024174S | deadwood | 60.4775 | 25.2406 | Europe | 2018 | 2018 | forest | gITS7/ITS4 | 5.1 | 670 | 4.3 | 16.1 | 57058 | 0 | 1037 |
| 4983 | 408 | GF04005592S | deadwood | 56.801 | 59.425 | Asia | 2017 | 2017 | forest | fITS7/ITS4 | 1.7 | 556 | 5.2 | 23.7 | 44399 | 0 | 4 |
| 8007 | 408 | GF04005650S | deadwood | 56.801 | 59.425 | Asia | 2017 | 2017 | forest | fITS7/ITS4 | 1.7 | 556 | 5.2 | 23.7 | 146689 | 0 | 1 |
| 11673 | 71 | GF05024197S | deadwood | 60.3277 | 24.7752 | Europe | 2018 | 2018 | forest | gITS7/ITS4 | 5.3 | 629 | 4.4 | 20.7 | 75002 | 0 | 2 |
| 12016 | 71 | GF05024171S | deadwood | 60.4777 | 25.2403 | Europe | 2018 | 2018 | forest | gITS7/ITS4 | 5.1 | 670 | 4.3 | 16.1 | 10476 | 0 | 7 |
| 13828 | 71 | GF05023983S | deadwood | 60.2626 | 24.9074 | Europe | 2018 | 2018 | forest | gITS7/ITS4 | 5.4 | 650 | 4.5 | 20.7 | 31381 | 0 | 17 |
| 14005 | 71 | GF05023952S | deadwood | 60.2641 | 24.7898 | Europe | 2018 | 2018 | forest | gITS7/ITS4 | 5.4 | 624 | 5.2 | 16.3 | 59099 | 0 | 7 |
| 15606 | 71 | GF05024130S | deadwood | 60.6233 | 25.1517 | Europe | 2018 | 2018 | forest | gITS7/ITS4 | 4.9 | 645 | 4.4 | 22.6 | 59437 | 0 | 19 |
| 20443 | 71 | GF05024051S | deadwood | 60.2063 | 24.9131 | Europe | 2018 | 2018 | forest | gITS7/ITS4 | 5.5 | 659 | 5.5 | 15.8 | 52492 | 0 | 10 |
| 22040 | 71 | GF05023891S | deadwood | 60.2242 | 25.1478 | Europe | 2018 | 2018 | forest | gITS7/ITS4 | 5.6 | 634 | 5.3 | 15 | 42606 | 0 | 24 |
| 22555 | 71 | GF05024017S | deadwood | 60.2486 | 24.9149 | Europe | 2018 | 2018 | forest | gITS7/ITS4 | 5.5 | 632 | 5 | 17.6 | 42785 | 0 | 1 |
| 22556 | 71 | GF05024029S | deadwood | 60.2241 | 24.9219 | Europe | 2018 | 2018 | forest | gITS7/ITS4 | 5.5 | 644 | 4.9 | 20.5 | 37772 | 0 | 6 |
| 27907 | 71 | GF05023858S | deadwood | 60.2218 | 25.047 | Europe | 2018 | 2018 | forest | gITS7/ITS4 | 5.6 | 641 | 4.9 | 17.5 | 29102 | 0 | 19 |
| 28429 | 71 | GF05024173S | deadwood | 60.4775 | 25.2406 | Europe | 2018 | 2018 | forest | gITS7/ITS4 | 5.1 | 670 | 4.3 | 16.1 | 37500 | 0 | 1 |
| 29626 | 71 | GF05024030S | deadwood | 60.2241 | 24.9219 | Europe | 2018 | 2018 | forest | gITS7/ITS4 | 5.5 | 644 | 4.9 | 20.5 | 41204 | 0 | 6 |
| 30648 | 71 | GF05024132S | deadwood | 60.6233 | 25.1517 | Europe | 2018 | 2018 | forest | gITS7/ITS4 | 4.9 | 645 | 4.4 | 22.6 | 51014 | 0 | 2 |
| 30915 | 71 | GF05024020S | deadwood | 60.2486 | 24.9149 | Europe | 2018 | 2018 | forest | gITS7/ITS4 | 5.5 | 632 | 5 | 17.6 | 38084 | 0 | 11 |
| 31533 | 71 | GF05023982S | deadwood | 60.2626 | 24.9074 | Europe | 2018 | 2018 | forest | gITS7/ITS4 | 5.4 | 650 | 4.5 | 20.7 | 30202 | 0 | 3 |
| 31535 | 71 | GF05024024S | deadwood | 60.2489 | 24.9155 | Europe | 2018 | 2018 | forest | gITS7/ITS4 | 5.5 | 632 | 5 | 17.6 | 49807 | 0 | 2 |
| 36152 | 71 | GF05023899S | deadwood | 60.2313 | 25.1573 | Europe | 2018 | 2018 | forest | gITS7/ITS4 | 5.6 | 633 | 5.2 | 17.3 | 39410 | 0 | 4 |
| 36153 | 71 | GF05024175S | deadwood | 60.4775 | 25.2406 | Europe | 2018 | 2018 | forest | gITS7/ITS4 | 5.1 | 670 | 4.3 | 16.1 | 50238 | 0 | 47 |
| 36154 | 71 | GF05023862S | deadwood | 60.222 | 25.0469 | Europe | 2018 | 2018 | forest | gITS7/ITS4 | 5.6 | 641 | 4.9 | 17.5 | 38240 | 0 | 3 |
| 37213 | 71 | GF05023948S | deadwood | 60.2643 | 24.79 | Europe | 2018 | 2018 | forest | gITS7/ITS4 | 5.4 | 624 | 5.2 | 16.3 | 47424 | 0 | 114 |
| 38307 | 71 | GF05024052S | deadwood | 60.2063 | 24.9131 | Europe | 2018 | 2018 | forest | gITS7/ITS4 | 5.5 | 659 | 5.5 | 15.8 | 46055 | 0 | 193 |
| 39015 | 71 | GF05023885S | deadwood | 60.2245 | 25.1478 | Europe | 2018 | 2018 | forest | gITS7/ITS4 | 5.6 | 634 | 5.3 | 15 | 73011 | 0 | 6 |
| 39018 | 71 | GF05024039S | deadwood | 60.2237 | 24.9294 | Europe | 2018 | 2018 | forest | gITS7/ITS4 | 5.5 | 658 | 5.2 | 18.5 | 38099 | 0 | 1 |
| 39019 | 71 | GF05024031S | deadwood | 60.2241 | 24.9219 | Europe | 2018 | 2018 | forest | gITS7/ITS4 | 5.5 | 644 | 4.9 | 20.5 | 53864 | 0 | 4 |
| 39020 | 71 | GF05023968S | deadwood | 60.2622 | 24.865 | Europe | 2018 | 2018 | forest | gITS7/ITS4 | 5.5 | 612 | 5.5 | 16.6 | 50542 | 0 | 3 |
| 39021 | 71 | GF05023889S | deadwood | 60.2242 | 25.1478 | Europe | 2018 | 2018 | forest | gITS7/ITS4 | 5.6 | 634 | 5.3 | 15 | 55003 | 0 | 1 |
| 39022 | 71 | GF05023890S | deadwood | 60.2242 | 25.1478 | Europe | 2018 | 2018 | forest | gITS7/ITS4 | 5.6 | 634 | 5.3 | 15 | 31630 | 0 | 6 |
| 42163 | 71 | GF05023943S | deadwood | 60.2784 | 24.8055 | Europe | 2018 | 2018 | forest | gITS7/ITS4 | 5.4 | 632 | 5.2 | 15.3 | 58611 | 0 | 194 |
| 42414 | 71 | GF05024078S | deadwood | 60.1977 | 25.1064 | Europe | 2018 | 2018 | forest | gITS7/ITS4 | 5.7 | 622 | 4.8 | 17.7 | 30045 | 0 | 1 |
| 43594 | 71 | GF05024056S | deadwood | 60.2387 | 25.1276 | Europe | 2018 | 2018 | forest | gITS7/ITS4 | 5.6 | 624 | 5.5 | 14.9 | 48915 | 0 | 79 |
| 46956 | 71 | GF05024179S | deadwood | 60.4774 | 25.2409 | Europe | 2018 | 2018 | forest | gITS7/ITS4 | 5.1 | 670 | 4.3 | 16.1 | 21989 | 0 | 60 |
| 48807 | 71 | GF05023857S | deadwood | 60.2218 | 25.047 | Europe | 2018 | 2018 | forest | gITS7/ITS4 | 5.6 | 641 | 4.9 | 17.5 | 37478 | 0 | 34 |
| 49084 | 71 | GF05023923S | deadwood | 60.1769 | 24.62 | Europe | 2018 | 2018 | forest | gITS7/ITS4 | 5.6 | 665 | 4.6 | 15.7 | 54690 | 0 | 1 |
| 51920 | 71 | GF05024087S | deadwood | 60.1966 | 25.017 | Europe | 2018 | 2018 | forest | gITS7/ITS4 | 5.7 | 604 | 5.1 | 16.2 | 42464 | 0 | 19 |
| 51921 | 71 | GF05024111S | deadwood | 60.1344 | 24.5553 | Europe | 2018 | 2018 | forest | gITS7/ITS4 | 5.6 | 666 | 4.4 | 20.9 | 35535 | 0 | 1 |
| 54068 | 71 | GF05023863S | deadwood | 60.222 | 25.0469 | Europe | 2018 | 2018 | forest | gITS7/ITS4 | 5.6 | 641 | 4.9 | 17.5 | 43821 | 0 | 2 |
| 56803 | 71 | GF05023997S | deadwood | 60.2717 | 24.9273 | Europe | 2018 | 2018 | forest | gITS7/ITS4 | 5.5 | 610 | 5.3 | 17.8 | 33965 | 0 | 1 |
| 56808 | 71 | GF05023988S | deadwood | 60.2624 | 24.9062 | Europe | 2018 | 2018 | forest | gITS7/ITS4 | 5.4 | 650 | 4.5 | 20 | 27435 | 0 | 2 |
| 56817 | 71 | GF05023924S | deadwood | 60.1769 | 24.62 | Europe | 2018 | 2018 | forest | gITS7/ITS4 | 5.6 | 665 | 4.6 | 15.7 | 51113 | 0 | 1 |
| 58424 | 71 | GF05024092S | deadwood | 60.1974 | 25.0166 | Europe | 2018 | 2018 | forest | gITS7/ITS4 | 5.7 | 604 | 5.1 | 16 | 39942 | 0 | 1 |
| 66712 | 71 | GF05023999S | deadwood | 60.2717 | 24.9273 | Europe | 2018 | 2018 | forest | gITS7/ITS4 | 5.5 | 610 | 5.3 | 17.8 | 32116 | 0 | 2 |


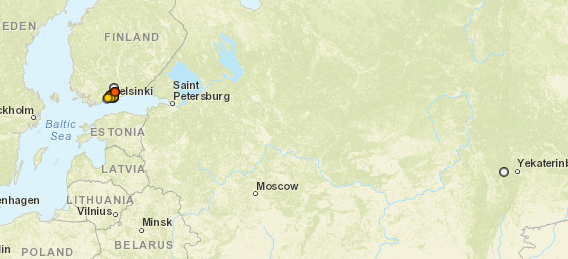


ITS1: TCGAGTTTTCACAACTCCCAaCCCTTGCGCACCGTACCCCAGTTTGTTCTCGTTGCTTCCTGGCGGGACGCCCGGGGGGGCCCCCAAAGGCCCCCCCAGGCTCCCAAATGCCCCGTCCGCGGGGCGCCCCGCCAGGGGCGGCGAGCCGCCTGAACCTTTTTTTTTACAAACAGCACACGAGCCTGTCTGAGCAAAACACACAATGAATCAA

No hits with 100-99.5%

**Supplementary material 12 B.** Geography of *Candida schatavii* studied using GlobalFungi. Primary data.

ITS1

ACAGTATTCTTTTGCCAGCGCTTAATTGCGCGGCGAAAAACCTTTCACACTATGTTTTTTATTAATTTGAAACTCTTGCTTTGGTCTGGCTTAGAAATAAGTTGGGCCAAAGGATATACCAAACTTCAATTTTTTAATTGAATTGTTATTTTAATTATTTTGTCAATTTGTTGATTAAATTCAAAAAATCTTCA

no hit above 93%

ITS2

CTCTCTCAAATCTTCGGATTTGGTTTTGAGTGATACTCTTAGTCGAACTAGGCGTTTGCTTGAAATGTATTGGCAAGAGTGGTACTAGATAGTGCTGAACTGTTATTCAATGTATTAGGTTTATCCAACTCGTTGAAATCAGATTGGTATTTGTTTATTACACAGGCTCGGCCTTACAACAACAAACAAAGT

Hits with100-99.5% similarity

| id | paper | permanent_id | sample_type | latitude | longitude | continent | year_of_sampling_from | year_of_sampling_to | Biome | primers | MAT | MAP | pH | SOC | ITS_total | manipulated | abundances | Country |
| --- | --- | --- | --- | --- | --- | --- | --- | --- | --- | --- | --- | --- | --- | --- | --- | --- | --- | --- |
| 15995 | 71 | GF05024034S | deadwood | 60,224 | 24,9295 | Europe | 2018 | 2018 | forest | gITS7/ITS4 | 5,5 | 658 | 5,2 | 18,5 | 60258 | 0 | 1 | Finland |
| 29626 | 71 | GF05024030S | deadwood | 60,2241 | 24,9219 | Europe | 2018 | 2018 | forest | gITS7/ITS4 | 5,5 | 644 | 4,9 | 20,5 | 41204 | 0 | 10 | Finland |
| 43357 | 71 | GF05023984S | deadwood | 60,2626 | 24,9074 | Europe | 2018 | 2018 | forest | gITS7/ITS4 | 5,4 | 650 | 4,5 | 20,7 | 42844 | 0 | 1 | Finland |
| 54068 | 71 | GF05023863S | deadwood | 60,222 | 25,0469 | Europe | 2018 | 2018 | forest | gITS7/ITS4 | 5,6 | 641 | 4,9 | 17,5 | 43821 | 0 | 74 | Finland |
| 67462 | 839 | GF01000667S | deadwood | 48,664 | 14,7052 | Europe | 2013 | 2013 | forest | gITS7/ITS4 | 6,6 | 784 | 4,5 | 21,5 | 3983 | 0 | 1 | Czech R. |


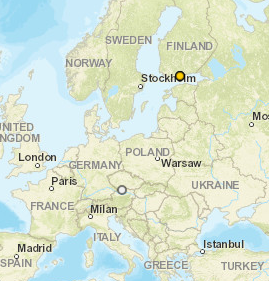


**Supplementary material 12 C,** Geography of *Blastobotrys sasensis* studied using GlobalFungi. Primary data.

ITS2 CAACTCTTAAATCAAATTTTTGATTTAGTATTGAGCCACATTTTTCGAGAGCAATCTTGAAAATGGTTTGAAATGTATATGGCTGAAGCAATTCCAGTATTTGGAGGCTTTGATTACAACGTAATAGGTTTTACCAACTCGTTGGCTTATCGAAGTTTTTCTTTTGGAATTTTCGCTTTTGCGTTATAAAATGACACTTAAAA

ITS1 ACGAAGTTTTGTCTGAAAAGACATTCTTTCAACTAAACACCTGTGAATTATTTACATCATTTTGCTTTGGTCAAACTTGTTTGGCCAAAAGTTCAAATTAAAACCTTTTATTTTTTAACCTTGATAGTCTGAAATTGAAATTTTTTAATTATTTA

| Sample ID | Longitude | Latitude | Elevation (m) | Continent | Country | Location | Sample Type | Biome | Biome Detail | Year of Sampling | Sampling Info | Sample Description | Dominant Plants | Authors | Year | DOI | MAT CHELSA (°C) | MAP CHELSA (mm) | ITS observed | ITS total |
| --- | --- | --- | --- | --- | --- | --- | --- | --- | --- | --- | --- | --- | --- | --- | --- | --- | --- | --- | --- | --- |
| GF01009573S | 25.2 | 60.45 | 50 | Europe | Finland | Rörstrand, Sipoo, Helsinki, Finland | deadwood | forest | coniferous forest biome | 2008 | NA_ | deadwood | Picea abies | Ovaskainen, O., Nokso-Koivisto, J., Hottola, J., Rajala, T., Pennanen, T., Ali-Kovero, H., Miettinen, O., Oinonen, P., Auvinen, P., Paulin, L. and Larsson, K.H., Mäkipää, R. | 2010 | 10.1016/j.funeco.2010.01.001 | 5.2 | 654 | 1 | 902 |
| GF05023872S | 25.0447 | 60.2271 | 26 | Europe | Finland | Helsinki | deadwood | forest | subpolar coniferous forest biome | 2018 | wood drilled from the inside of a downed trunk of Picea abies, sampling point 8 m from the base toward the top of the trunk, trunk diameter at sampling point 25.3 cm | deadwood | Picea abies | Korhonen, A., Miettinen, O., Kotze, J.D. and Hamberg, L. | 2022 | 10.1111/1462-2920.15903 | 5.6 | 640 | 112 | 52311 |
| GF04005650S | 59.425 | 56.801 | 392 | Asia | Russia | Sverdlovsk Oblast, Nizhneserginsky District | deadwood | forest | temperate evergreen needleleaf forest | 2017 | a disk was cut out with a chainsaw from three sampling points more than 1 m apart from each other on a log of at least 10-cm base diameter that was in contact with the ground and undamaged by large animals, and 10 g of wood fragments was extracted from five points of each disk with bleach-sterilized forceps and pooled in a sterile plastic container | deadwood | NA_ | Mikryukov, V.S., Dulya, O.V., Bergman, I.E., Lihodeevskiy, G.A., Loginova, A.D. and Tedersoo, L. | 2021 | 10.3389/fmicb.2021.729244 | 1.7 | 556 | 6 | 146689 |
| GF05024171S | 25.2403 | 60.4777 | 58 | Europe | Finland | Sipoo | deadwood | forest | subpolar coniferous forest biome | 2018 | wood drilled from the inside of a downed trunk of Picea abies, sampling point 6 m from the base toward the top of the trunk, trunk diameter at sampling point 28.6 cm | deadwood | Picea abies | Korhonen, A., Miettinen, O., Kotze, J.D. and Hamberg, L. | 2022 | 10.1111/1462-2920.15903 | 5.1 | 670 | 2 | 10476 |
| GF05024120S | 24.5562 | 60.1355 | 34 | Europe | Finland | Kirkkonummi | deadwood | forest | subpolar coniferous forest biome | 2018 | wood drilled from the inside of a downed trunk of Picea abies, sampling point 8 m from the base toward the top of the trunk, trunk diameter at sampling point 27.8 cm | deadwood | Picea abies | Korhonen, A., Miettinen, O., Kotze, J.D. and Hamberg, L. | 2022 | 10.1111/1462-2920.15903 | 5.6 | 666 | 2 | 38667 |
| GF05023983S | 24.9074 | 60.2626 | 39 | Europe | Finland | Helsinki | deadwood | forest | subpolar coniferous forest biome | 2018 | wood drilled from the inside of a downed trunk of Picea abies, sampling point 6 m from the base toward the top of the trunk, trunk diameter at sampling point 24.9 cm | deadwood | Picea abies | Korhonen, A., Miettinen, O., Kotze, J.D. and Hamberg, L. | 2022 | 10.1111/1462-2920.15903 | 5.4 | 650 | 660 | 31381 |
| GF05023996S | 25.0409 | 60.3618 | 57 | Europe | Finland | Helsinki | deadwood | forest | subpolar coniferous forest biome | 2018 | wood drilled from the inside of a downed trunk of Picea abies, sampling point 8 m from the base toward the top of the trunk, trunk diameter at sampling point 23.3 cm | deadwood | Picea abies | Korhonen, A., Miettinen, O., Kotze, J.D. and Hamberg, L. | 2022 | 10.1111/1462-2920.15903 | 5.3 | 625 | 42 | 43524 |
| GF05024034S | 24.9295 | 60.224 | 27 | Europe | Finland | Helsinki | deadwood | forest | subpolar coniferous forest biome | 2018 | wood drilled from the inside of a downed trunk of Picea abies, sampling point 4 m from the base toward the top of the trunk, trunk diameter at sampling point 27 cm | deadwood | Picea abies | Korhonen, A., Miettinen, O., Kotze, J.D. and Hamberg, L. | 2022 | 10.1111/1462-2920.15903 | 5.5 | 658 | 5 | 60258 |
| GF05023884S | 25.1564 | 60.2481 | 11 | Europe | Finland | Vantaa | deadwood | forest | subpolar coniferous forest biome | 2018 | wood drilled from the inside of a downed trunk of Picea abies, sampling point 8 m from the base toward the top of the trunk, trunk diameter at sampling point 22.2 cm | deadwood | Picea abies | Korhonen, A., Miettinen, O., Kotze, J.D. and Hamberg, L. | 2022 | 10.1111/1462-2920.15903 | 5.6 | 632 | 59 | 39174 |
| GF05024036S | 24.9295 | 60.224 | 27 | Europe | Finland | Helsinki | deadwood | forest | subpolar coniferous forest biome | 2018 | wood drilled from the inside of a downed trunk of Picea abies, sampling point 8 m from the base toward the top of the trunk, trunk diameter at sampling point 22.5 cm | deadwood | Picea abies | Korhonen, A., Miettinen, O., Kotze, J.D. and Hamberg, L. | 2022 | 10.1111/1462-2920.15903 | 5.5 | 658 | 4 | 58541 |
| GF05023972S | 24.8649 | 60.2635 | 30 | Europe | Finland | Vantaa | deadwood | forest | subpolar coniferous forest biome | 2018 | wood drilled from the inside of a downed trunk of Picea abies, sampling point 8 m from the base toward the top of the trunk, trunk diameter at sampling point 22.3 cm | deadwood | Picea abies | Korhonen, A., Miettinen, O., Kotze, J.D. and Hamberg, L. | 2022 | 10.1111/1462-2920.15903 | 5.5 | 612 | 83 | 45954 |
| GF05023964S | 24.866 | 60.2622 | 41 | Europe | Finland | Vantaa | deadwood | forest | subpolar coniferous forest biome | 2018 | wood drilled from the inside of a downed trunk of Picea abies, sampling point 8 m from the base toward the top of the trunk, trunk diameter at sampling point 23.5 cm | deadwood | Picea abies | Korhonen, A., Miettinen, O., Kotze, J.D. and Hamberg, L. | 2022 | 10.1111/1462-2920.15903 | 5.5 | 612 | 348 | 59159 |
| GF05023973S | 24.866 | 60.2639 | 31 | Europe | Finland | Vantaa | deadwood | forest | subpolar coniferous forest biome | 2018 | wood drilled from the inside of a downed trunk of Picea abies, sampling point 2 m from the base toward the top of the trunk, trunk diameter at sampling point 34.8 cm | deadwood | Picea abies | Korhonen, A., Miettinen, O., Kotze, J.D. and Hamberg, L. | 2022 | 10.1111/1462-2920.15903 | 5.5 | 612 | 4 | 45509 |
| GF05024050S | 24.9131 | 60.2063 | 22 | Europe | Finland | Helsinki | deadwood | forest | subpolar coniferous forest biome | 2018 | wood drilled from the inside of a downed trunk of Picea abies, sampling point 4 m from the base toward the top of the trunk, trunk diameter at sampling point 30.6 cm | deadwood | Picea abies | Korhonen, A., Miettinen, O., Kotze, J.D. and Hamberg, L. | 2022 | 10.1111/1462-2920.15903 | 5.5 | 659 | 7 | 48888 |
| GF05024043S | 24.9123 | 60.2083 | 22 | Europe | Finland | Helsinki | deadwood | forest | subpolar coniferous forest biome | 2018 | wood drilled from the inside of a downed trunk of Picea abies, sampling point 6 m from the base toward the top of the trunk, trunk diameter at sampling point 29.3 cm | deadwood | Picea abies | Korhonen, A., Miettinen, O., Kotze, J.D. and Hamberg, L. | 2022 | 10.1111/1462-2920.15903 | 5.6 | 647 | 1 | 52141 |
| GF05024030S | 24.9219 | 60.2241 | 28 | Europe | Finland | Helsinki | deadwood | forest | subpolar coniferous forest biome | 2018 | wood drilled from the inside of a downed trunk of Picea abies, sampling point 4 m from the base toward the top of the trunk, trunk diameter at sampling point 28.2 cm | deadwood | Picea abies | Korhonen, A., Miettinen, O., Kotze, J.D. and Hamberg, L. | 2022 | 10.1111/1462-2920.15903 | 5.5 | 644 | 8910 | 41204 |
| GF05023864S | 25.0469 | 60.222 | 28 | Europe | Finland | Helsinki | deadwood | forest | subpolar coniferous forest biome | 2018 | wood drilled from the inside of a downed trunk of Picea abies, sampling point 8 m from the base toward the top of the trunk, trunk diameter at sampling point 29.6 cm | deadwood | Picea abies | Korhonen, A., Miettinen, O., Kotze, J.D. and Hamberg, L. | 2022 | 10.1111/1462-2920.15903 | 5.6 | 641 | 143 | 25572 |
| GF05024020S | 24.9149 | 60.2486 | 30 | Europe | Finland | Helsinki | deadwood | forest | subpolar coniferous forest biome | 2018 | wood drilled from the inside of a downed trunk of Picea abies, sampling point 8 m from the base toward the top of the trunk, trunk diameter at sampling point 22.3 cm | deadwood | Picea abies | Korhonen, A., Miettinen, O., Kotze, J.D. and Hamberg, L. | 2022 | 10.1111/1462-2920.15903 | 5.5 | 632 | 51 | 38084 |
| GF05023982S | 24.9074 | 60.2626 | 39 | Europe | Finland | Helsinki | deadwood | forest | subpolar coniferous forest biome | 2018 | wood drilled from the inside of a downed trunk of Picea abies, sampling point 4 m from the base toward the top of the trunk, trunk diameter at sampling point 25.9 cm | deadwood | Picea abies | Korhonen, A., Miettinen, O., Kotze, J.D. and Hamberg, L. | 2022 | 10.1111/1462-2920.15903 | 5.4 | 650 | 50 | 30202 |
| GF05023987S | 24.9062 | 60.2624 | 32 | Europe | Finland | Helsinki | deadwood | forest | subpolar coniferous forest biome | 2018 | wood drilled from the inside of a downed trunk of Picea abies, sampling point 6 m from the base toward the top of the trunk, trunk diameter at sampling point 25.9 cm | deadwood | Picea abies | Korhonen, A., Miettinen, O., Kotze, J.D. and Hamberg, L. | 2022 | 10.1111/1462-2920.15903 | 5.4 | 650 | 1 | 32215 |
| GF05024140S | 25.4116 | 60.6429 | 85 | Europe | Finland | Mäntsälä | deadwood | forest | subpolar coniferous forest biome | 2018 | wood drilled from the inside of a downed trunk of Picea abies, sampling point 8 m from the base toward the top of the trunk, trunk diameter at sampling point 23.3 cm | deadwood | Picea abies | Korhonen, A., Miettinen, O., Kotze, J.D. and Hamberg, L. | 2022 | 10.1111/1462-2920.15903 | 5 | 652 | 5 | 81784 |
| GF05023963S | 24.866 | 60.2622 | 41 | Europe | Finland | Vantaa | deadwood | forest | subpolar coniferous forest biome | 2018 | wood drilled from the inside of a downed trunk of Picea abies, sampling point 6 m from the base toward the top of the trunk, trunk diameter at sampling point 28.4 cm | deadwood | Picea abies | Korhonen, A., Miettinen, O., Kotze, J.D. and Hamberg, L. | 2022 | 10.1111/1462-2920.15903 | 5.5 | 612 | 8 | 48017 |
| GF05023900S | 25.1573 | 60.2313 | 12 | Europe | Finland | Helsinki | deadwood | forest | subpolar coniferous forest biome | 2018 | wood drilled from the inside of a downed trunk of Picea abies, sampling point 8 m from the base toward the top of the trunk, trunk diameter at sampling point 25.2 cm | deadwood | Picea abies | Korhonen, A., Miettinen, O., Kotze, J.D. and Hamberg, L. | 2022 | 10.1111/1462-2920.15903 | 5.6 | 633 | 934 | 29816 |
| GF05024102S | 25.023 | 60.2124 | 8 | Europe | Finland | Helsinki | deadwood | forest | subpolar coniferous forest biome | 2018 | wood drilled from the inside of a downed trunk of Picea abies, sampling point 4 m from the base toward the top of the trunk, trunk diameter at sampling point 38 cm | deadwood | Picea abies | Korhonen, A., Miettinen, O., Kotze, J.D. and Hamberg, L. | 2022 | 10.1111/1462-2920.15903 | 5.7 | 601 | 15 | 60298 |
| GF05024072S | 25.1067 | 60.1975 | 16 | Europe | Finland | Helsinki | deadwood | forest | subpolar coniferous forest biome | 2018 | wood drilled from the inside of a downed trunk of Picea abies, sampling point 8 m from the base toward the top of the trunk, trunk diameter at sampling point 23.7 cm | deadwood | Picea abies | Korhonen, A., Miettinen, O., Kotze, J.D. and Hamberg, L. | 2022 | 10.1111/1462-2920.15903 | 5.7 | 622 | 2 | 36726 |
| GF05023885S | 25.1478 | 60.2245 | 8 | Europe | Finland | Helsinki | deadwood | forest | subpolar coniferous forest biome | 2018 | wood drilled from the inside of a downed trunk of Picea abies, sampling point 2 m from the base toward the top of the trunk, trunk diameter at sampling point 26.3 cm | deadwood | Picea abies | Korhonen, A., Miettinen, O., Kotze, J.D. and Hamberg, L. | 2022 | 10.1111/1462-2920.15903 | 5.6 | 634 | 3 | 73011 |
| GF05024039S | 24.9294 | 60.2237 | 24 | Europe | Finland | Helsinki | deadwood | forest | subpolar coniferous forest biome | 2018 | wood drilled from the inside of a downed trunk of Picea abies, sampling point 6 m from the base toward the top of the trunk, trunk diameter at sampling point 26.6 cm | deadwood | Picea abies | Korhonen, A., Miettinen, O., Kotze, J.D. and Hamberg, L. | 2022 | 10.1111/1462-2920.15903 | 5.5 | 658 | 2 | 38099 |
| GF05024031S | 24.9219 | 60.2241 | 28 | Europe | Finland | Helsinki | deadwood | forest | subpolar coniferous forest biome | 2018 | wood drilled from the inside of a downed trunk of Picea abies, sampling point 6 m from the base toward the top of the trunk, trunk diameter at sampling point 25.9 cm | deadwood | Picea abies | Korhonen, A., Miettinen, O., Kotze, J.D. and Hamberg, L. | 2022 | 10.1111/1462-2920.15903 | 5.5 | 644 | 46 | 53864 |
| GF05024088S | 25.017 | 60.1966 | 9 | Europe | Finland | Helsinki | deadwood | forest | subpolar coniferous forest biome | 2018 | wood drilled from the inside of a downed trunk of Picea abies, sampling point 8 m from the base toward the top of the trunk, trunk diameter at sampling point 23.4 cm | deadwood | Picea abies | Korhonen, A., Miettinen, O., Kotze, J.D. and Hamberg, L. | 2022 | 10.1111/1462-2920.15903 | 5.7 | 604 | 2 | 41850 |
| GF05023929S | 24.8085 | 60.2803 | 41 | Europe | Finland | Vantaa | deadwood | forest | subpolar coniferous forest biome | 2018 | wood drilled from the inside of a downed trunk of Picea abies, sampling point 2 m from the base toward the top of the trunk, trunk diameter at sampling point 41 cm | deadwood | Picea abies | Korhonen, A., Miettinen, O., Kotze, J.D. and Hamberg, L. | 2022 | 10.1111/1462-2920.15903 | 5.4 | 624 | 1 | 54554 |
| GF05023981S | 24.9074 | 60.2626 | 39 | Europe | Finland | Helsinki | deadwood | forest | subpolar coniferous forest biome | 2018 | wood drilled from the inside of a downed trunk of Picea abies, sampling point 2 m from the base toward the top of the trunk, trunk diameter at sampling point 27.4 cm | deadwood | Picea abies | Korhonen, A., Miettinen, O., Kotze, J.D. and Hamberg, L. | 2022 | 10.1111/1462-2920.15903 | 5.4 | 650 | 2 | 22160 |
| GF05023869S | 25.0447 | 60.2271 | 26 | Europe | Finland | Helsinki | deadwood | forest | subpolar coniferous forest biome | 2018 | wood drilled from the inside of a downed trunk of Picea abies, sampling point 2 m from the base toward the top of the trunk, trunk diameter at sampling point 30.8 cm | deadwood | Picea abies | Korhonen, A., Miettinen, O., Kotze, J.D. and Hamberg, L. | 2022 | 10.1111/1462-2920.15903 | 5.6 | 640 | 6 | 39289 |
| GF05023866S | 25.0444 | 60.2269 | 25 | Europe | Finland | Helsinki | deadwood | forest | subpolar coniferous forest biome | 2018 | wood drilled from the inside of a downed trunk of Picea abies, sampling point 4 m from the base toward the top of the trunk, trunk diameter at sampling point 24.5 cm | deadwood | Picea abies | Korhonen, A., Miettinen, O., Kotze, J.D. and Hamberg, L. | 2022 | 10.1111/1462-2920.15903 | 5.6 | 640 | 1 | 39307 |
| GF05024103S | 25.023 | 60.2124 | 8 | Europe | Finland | Helsinki | deadwood | forest | subpolar coniferous forest biome | 2018 | wood drilled from the inside of a downed trunk of Picea abies, sampling point 6 m from the base toward the top of the trunk, trunk diameter at sampling point 32.7 cm | deadwood | Picea abies | Korhonen, A., Miettinen, O., Kotze, J.D. and Hamberg, L. | 2022 | 10.1111/1462-2920.15903 | 5.7 | 601 | 2110 | 45499 |
| GF05023984S | 24.9074 | 60.2626 | 39 | Europe | Finland | Helsinki | deadwood | forest | subpolar coniferous forest biome | 2018 | wood drilled from the inside of a downed trunk of Picea abies, sampling point 8 m from the base toward the top of the trunk, trunk diameter at sampling point 23.2 cm | deadwood | Picea abies | Korhonen, A., Miettinen, O., Kotze, J.D. and Hamberg, L. | 2022 | 10.1111/1462-2920.15903 | 5.4 | 650 | 36 | 42844 |
| GF05024100S | 25.02 | 60.197 | 5 | Europe | Finland | Helsinki | deadwood | forest | subpolar coniferous forest biome | 2018 | wood drilled from the inside of a downed trunk of Picea abies, sampling point 8 m from the base toward the top of the trunk, trunk diameter at sampling point 30.7 cm | deadwood | Picea abies | Korhonen, A., Miettinen, O., Kotze, J.D. and Hamberg, L. | 2022 | 10.1111/1462-2920.15903 | 5.7 | 604 | 18 | 45343 |
| GF05024153S | 25.1917 | 60.4595 | 61 | Europe | Finland | Sipoo | deadwood | forest | subpolar coniferous forest biome | 2018 | wood drilled from the inside of a downed trunk of Picea abies, sampling point 2 m from the base toward the top of the trunk, trunk diameter at sampling point 32.6 cm | deadwood | Picea abies | Korhonen, A., Miettinen, O., Kotze, J.D. and Hamberg, L. | 2022 | 10.1111/1462-2920.15903 | 5.2 | 655 | 4 | 60678 |
| GF05024158S | 24.5039 | 60.3317 | 42 | Europe | Finland | Vihti | deadwood | forest | subpolar coniferous forest biome | 2018 | wood drilled from the inside of a downed trunk of Picea abies, sampling point 4 m from the base toward the top of the trunk, trunk diameter at sampling point 33 cm | deadwood | Picea abies | Korhonen, A., Miettinen, O., Kotze, J.D. and Hamberg, L. | 2022 | 10.1111/1462-2920.15903 | 5.2 | 626 | 3 | 42612 |
| GF05024013S | 24.9122 | 60.2484 | 30 | Europe | Finland | Helsinki | deadwood | forest | subpolar coniferous forest biome | 2018 | wood drilled from the inside of a downed trunk of Picea abies, sampling point 2 m from the base toward the top of the trunk, trunk diameter at sampling point 31.6 cm | deadwood | Picea abies | Korhonen, A., Miettinen, O., Kotze, J.D. and Hamberg, L. | 2022 | 10.1111/1462-2920.15903 | 5.5 | 632 | 1 | 47096 |
| GF05024165S | 24.5028 | 60.3327 | 51 | Europe | Finland | Vihti | deadwood | forest | subpolar coniferous forest biome | 2018 | wood drilled from the inside of a downed trunk of Picea abies, sampling point 2 m from the base toward the top of the trunk, trunk diameter at sampling point 33.5 cm | deadwood | Picea abies | Korhonen, A., Miettinen, O., Kotze, J.D. and Hamberg, L. | 2022 | 10.1111/1462-2920.15903 | 5.2 | 626 | 20 | 32564 |
| GF05024032S | 24.9219 | 60.2241 | 28 | Europe | Finland | Helsinki | deadwood | forest | subpolar coniferous forest biome | 2018 | wood drilled from the inside of a downed trunk of Picea abies, sampling point 8 m from the base toward the top of the trunk, trunk diameter at sampling point 23.7 cm | deadwood | Picea abies | Korhonen, A., Miettinen, O., Kotze, J.D. and Hamberg, L. | 2022 | 10.1111/1462-2920.15903 | 5.5 | 644 | 3 | 41255 |
| GF05023923S | 24.62 | 60.1769 | 19 | Europe | Finland | Espoo | deadwood | forest | subpolar coniferous forest biome | 2018 | wood drilled from the inside of a downed trunk of Picea abies, sampling point 6 m from the base toward the top of the trunk, trunk diameter at sampling point 34.2 cm | deadwood | Picea abies | Korhonen, A., Miettinen, O., Kotze, J.D. and Hamberg, L. | 2022 | 10.1111/1462-2920.15903 | 5.6 | 665 | 383 | 54690 |
| GF05024201S | 24.7751 | 60.3283 | 45 | Europe | Finland | Vantaa | deadwood | forest | subpolar coniferous forest biome | 2018 | wood drilled from the inside of a downed trunk of Picea abies, sampling point 2 m from the base toward the top of the trunk, trunk diameter at sampling point 29.3 cm | deadwood | Picea abies | Korhonen, A., Miettinen, O., Kotze, J.D. and Hamberg, L. | 2022 | 10.1111/1462-2920.15903 | 5.3 | 629 | 1 | 83023 |
| GF05023863S | 25.0469 | 60.222 | 28 | Europe | Finland | Helsinki | deadwood | forest | subpolar coniferous forest biome | 2018 | wood drilled from the inside of a downed trunk of Picea abies, sampling point 6 m from the base toward the top of the trunk, trunk diameter at sampling point 29.7 cm | deadwood | Picea abies | Korhonen, A., Miettinen, O., Kotze, J.D. and Hamberg, L. | 2022 | 10.1111/1462-2920.15903 | 5.6 | 641 | 60 | 43821 |
| GF05024025S | 24.9225 | 60.2245 | 19 | Europe | Finland | Helsinki | deadwood | forest | subpolar coniferous forest biome | 2018 | wood drilled from the inside of a downed trunk of Picea abies, sampling point 2 m from the base toward the top of the trunk, trunk diameter at sampling point 37.5 cm | deadwood | Picea abies | Korhonen, A., Miettinen, O., Kotze, J.D. and Hamberg, L. | 2022 | 10.1111/1462-2920.15903 | 5.5 | 644 | 1 | 49088 |
| GF05024033S | 24.9295 | 60.224 | 27 | Europe | Finland | Helsinki | deadwood | forest | subpolar coniferous forest biome | 2018 | wood drilled from the inside of a downed trunk of Picea abies, sampling point 2 m from the base toward the top of the trunk, trunk diameter at sampling point 29.5 cm | deadwood | Picea abies | Korhonen, A., Miettinen, O., Kotze, J.D. and Hamberg, L. | 2022 | 10.1111/1462-2920.15903 | 5.5 | 658 | 3 | 43597 |
| GF05023894S | 25.1571 | 60.2303 | 10 | Europe | Finland | Helsinki | deadwood | forest | subpolar coniferous forest biome | 2018 | wood drilled from the inside of a downed trunk of Picea abies, sampling point 4 m from the base toward the top of the trunk, trunk diameter at sampling point 31.5 cm | deadwood | Picea abies | Korhonen, A., Miettinen, O., Kotze, J.D. and Hamberg, L. | 2022 | 10.1111/1462-2920.15903 | 5.6 | 633 | 1 | 57535 |
| GF05024071S | 25.1067 | 60.1975 | 16 | Europe | Finland | Helsinki | deadwood | forest | subpolar coniferous forest biome | 2018 | wood drilled from the inside of a downed trunk of Picea abies, sampling point 6 m from the base toward the top of the trunk, trunk diameter at sampling point 26.5 cm | deadwood | Picea abies | Korhonen, A., Miettinen, O., Kotze, J.D. and Hamberg, L. | 2022 | 10.1111/1462-2920.15903 | 5.7 | 622 | 1 | 48945 |
| GF05024046S | 24.9123 | 60.2079 | 21 | Europe | Finland | Helsinki | deadwood | forest | subpolar coniferous forest biome | 2018 | wood drilled from the inside of a downed trunk of Picea abies, sampling point 4 m from the base toward the top of the trunk, trunk diameter at sampling point 27.4 cm | deadwood | Picea abies | Korhonen, A., Miettinen, O., Kotze, J.D. and Hamberg, L. | 2022 | 10.1111/1462-2920.15903 | 5.5 | 659 | 2 | 43166 |
| GF05023997S | 24.9273 | 60.2717 | 29 | Europe | Finland | Helsinki | deadwood | forest | subpolar coniferous forest biome | 2018 | wood drilled from the inside of a downed trunk of Picea abies, sampling point 2 m from the base toward the top of the trunk, trunk diameter at sampling point 30.9 cm | deadwood | Picea abies | Korhonen, A., Miettinen, O., Kotze, J.D. and Hamberg, L. | 2022 | 10.1111/1462-2920.15903 | 5.5 | 610 | 1 | 33965 |
| GF05023924S | 24.62 | 60.1769 | 19 | Europe | Finland | Espoo | deadwood | forest | subpolar coniferous forest biome | 2018 | wood drilled from the inside of a downed trunk of Picea abies, sampling point 8 m from the base toward the top of the trunk, trunk diameter at sampling point 31.7 cm | deadwood | Picea abies | Korhonen, A., Miettinen, O., Kotze, J.D. and Hamberg, L. | 2022 | 10.1111/1462-2920.15903 | 5.6 | 665 | 3 | 51113 |
| GF05024104S | 25.023 | 60.2124 | 8 | Europe | Finland | Helsinki | deadwood | forest | subpolar coniferous forest biome | 2018 | wood drilled from the inside of a downed trunk of Picea abies, sampling point 8 m from the base toward the top of the trunk, trunk diameter at sampling point 31.5 cm | deadwood | Picea abies | Korhonen, A., Miettinen, O., Kotze, J.D. and Hamberg, L. | 2022 | 10.1111/1462-2920.15903 | 5.7 | 601 | 3 | 43228 |
| GF05024092S | 25.0166 | 60.1974 | 11 | Europe | Finland | Helsinki | deadwood | forest | subpolar coniferous forest biome | 2018 | wood drilled from the inside of a downed trunk of Picea abies, sampling point 8 m from the base toward the top of the trunk, trunk diameter at sampling point 29.7 cm | deadwood | Picea abies | Korhonen, A., Miettinen, O., Kotze, J.D. and Hamberg, L. | 2022 | 10.1111/1462-2920.15903 | 5.7 | 604 | 681 | 39942 |
| GF05023861S | 25.0469 | 60.222 | 28 | Europe | Finland | Helsinki | deadwood | forest | subpolar coniferous forest biome | 2018 | wood drilled from the inside of a downed trunk of Picea abies, sampling point 2 m from the base toward the top of the trunk, trunk diameter at sampling point 33.7 cm | deadwood | Picea abies | Korhonen, A., Miettinen, O., Kotze, J.D. and Hamberg, L. | 2022 | 10.1111/1462-2920.15903 | 5.6 | 641 | 124 | 32624 |


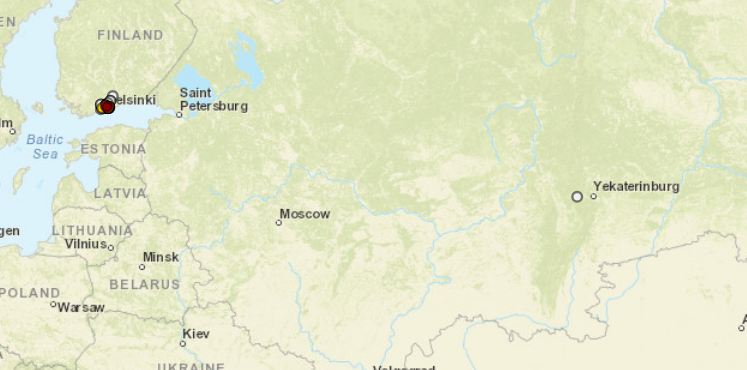

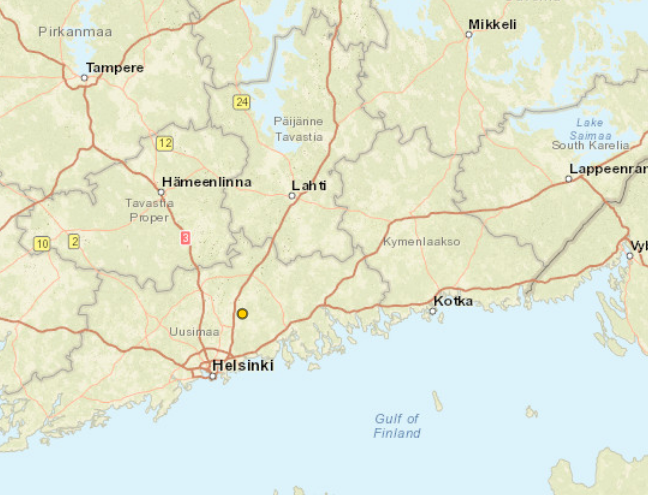


**Supplementary material 12 D.** Geography of *Sugiyamaella casensis* studied using GlobalFungi. Primary data.

ITS1 ATACACCTGTGAaTCTTTGACTTATTTGCTTTGCCCTTTTTTTTCTTTTTTCTTCGGGAAAGGGAACGGGGGGGGGCAAAGGATTTCAAACTTTTTTTTTTTTTTTGT

ITS1 ATACACCTGTGATCTTTGACTTATTTGCTTTGCCCTTTTTTTTCTTTTTTCTTCGGGAAAGGGAACGGGGGGGGGCAAAGGATTTCAAACTTTTTTTTTTTTTTTGT

ITS2 CATTTCTTTCTCAAAACTTTAGTTTTGGTCTTGATTGATTAGCTACTTTATTGTGGCTATTGATTGAAAGAAATTGGCATAGGCAGTTTATTTGTTTTAACAACGTATTAGGTTCTACCAACTCGTTGGCCAAAACATTTTGAACAATCGCCTAGCGGTACAAATTTTTCAAAATTTTtAAAAAT

ITS2 CATTTCTTTCTCAAAACTTTAGTTTTGGTCTTGATTGATTAGCTACTTTATTGTGGCTATTGATTGAAAGAAATTGGCATAGGCAGTTTATTTGTTTTAACAACGTATTAGGTTCTACCAACTCGTTGGCCAAAACATTTTGAACAATCGCCTAGCGGTACAAATTTTTCAAAATTTTAAAAAT

no exact hit

no hit above 90%
