# Supplementary material for: The ambrosial mycobiota of Treptoplatypus oxyurus (Coleoptera, Platypodidae): a unique island of fungal diversity revealing Wilhelmdebeerea oxyuri gen. et sp. nov. (Ophiostomatales), and two new yeast species Blastobotrys sasensis sp. nov., and Sugiyamaella casensis sp. nov. (Dipodascales)
Source: IMA Fungus. 2026 Feb 16;17:e177075. doi: 10.3897/imafungus.17.177075 (PMC12930180; doi:10.3897/imafungus.17.177075)
Supplement: Supplementary material 13 — Physiological characteristic of the new yeast species [file imafungus-17-e177075-s013.docx]

**Supplementary material 13.** Physiological characteristics of the new yeast species. **Table S1.** Physiological and biochemical properties of new species. **Table S2.** Comparison of the physiological and biochemical properties of two new species with their closely related species.

**Table S1.** Physiological and biochemical properties of new species.

| Characteristic | ***Blastobotrys sasensis* sp. nov.** | ***Sugiyamaella casensis* sp. nov.** |
| --- | --- | --- |
| Fermentation of: |  |  |
| Glucose | - | **-** |
| Saccharose | - | **-** |
| Maltose | - | **-** |
| Lactose | - | **-** |
| Raffinose | - | **-** |
| d-Xylose | - | **-** |
| Galactose | - | **-** |
| Assimilation of carbon compounds: |  |  |
| Glucose | + | + |
| Fructose | + | + |
| Mannose | + | + |
| Galactose | + | + |
| Maltose | + | + |
| Saccharose | + | + |
| Lactose | + | - |
| Raffinose | + | + |
| Melezitose | - | + |
| d-Xylose | + | + |
| l-Arabinose | - | + |
| Inulin | - | - |
| Cellobiose | + | + |
| Trehalose | + | + |
| Soluble starch | + | + |
| Melibiose | + | + |
| l-Rhamnose | - | + |
| l-Sorbose | + | + |
| d-Arabinose | - | - |
| d-Ribose | - | + |
| Salicin | + | + |
| Xylitol | - | + |
| Ribitol | + | + |
| d-Mannitol | + | + |
| d-Glucitol | + | + |
| Erythritol | + | + |
| *myo*-Inositol | - | + |
| Ethanol | - | + |
| Methanol | - | - |
| Glycerol | + | + |
| n-acetyl-d-glukosamine | w | + |
| Succinic acid | + | + |
| dl-Lactate | - | - |
| Citrate | + | + |
| Assimilation of nitrogen compounds: |  |  |
| Nitrate | - | - |
| Nitrite | - | - |
| Ethylamine | + | + |
| l-Lysine | + | - |
| Cadaverine | + | - |
| Creatinine | - | - |
| n-acetyl-d-glucosamine | + | + |
| Other tests |  |  |
| Starch formation | - | - |
| Urease production | - | - |
| Growth at/in: |  |  |
| vitamin free-medium | w | + |
| 10% NaCl+5% glucose | + | w |
| 50% Glucose | + | + |
| 5°C | + | w |
| 10 °C | + | w |
| 20°C | + | + |
| 28°C | + | + |
| 30°C | + | + |
| 35 °C | - | - |
| Note: +, positive; -, negative; w, weak growth | | |

**Table S2.** Comparison of the physiological and biochemical properties of two new species with their closely related species

| Characteristic | ***Blastobotrys sasensis* sp. nov.** | *Blastobotrys robertii* | *Blastobotrys muscicola* | ***Sugiyamaella casensis* sp. nov.** | *Sugiyamaella mastotermitis* |
| --- | --- | --- | --- | --- | --- |
| Fermentation of: |  |  |  |  |  |
| Glucose |  |  |  | **-** | + |
| Saccharose |  |  |  | **-** | + |
| Maltose |  |  |  | **-** | + |
| Galactose |  |  |  | **-** | + |
| Assimilation of carbon compounds: |  |  |  |  |  |
| Melezitose | - | - | + |  |  |
| D-Xylose | + | - | - |  |  |
| Soluble starch |  |  |  | + | w (96 h) |
| L-Sorbose | + | - | - |  |  |
| D-Ribose |  |  |  | + | - |
| Xylitol | - | - | n |  |  |
| Ribitol | + | - | - | + | - |
| D-Glucitol | + | - | - | + | n |
| Erythritol | + | - | - |  |  |
| *Myo*-Inositol |  |  |  | + | w |
| Ethanol |  |  |  | + | n |
| Methanol |  |  |  | - | n |
| Glycerol | + | - | + | + | - |
| N-acetyl-D-glukosamine | w | + | + |  |  |
| Succinic acid |  |  |  | + | n |
| Citrate | + | n | + | + | n |
| Assimilation of nitrogen compounds: |  |  |  |  |  |
| Nitrate |  |  |  | - | n |
| Nitrite | - | - | n | - | n |
| Ethylamine | + | + | n | + | n |
| L-Lysine | + | + | n | - | n |
| Cadaverine | + | + | n | - | n |
| Creatinine | - | - | n | - | n |
| N-acetyl-D-glucosamine | + | n | n | + | n |
| Growth at/in: |  |  |  |  |  |
| vitamin free-medium | w | - | v |  |  |
| 10% NaCl+5% glucose | + | - | n | w | - |
| 50% Glucose | + | n | n | + | - |
| 37°C | - | - | + | - | + |
| 40°C |  |  |  | - | + |
| Note: +, positive; -, negative; d, delayed growth (after 14 days); w, weak growth; n, no data available; | | | | |  |
| data from closely related species were taken from: Kurtzman (2007), Middelhoven and Kurtzman (2007), Handel et al. (2016) | | | | | |

**References**

Handel S, Wang T, Yurkov AM et al. (2016) *Sugiyamaella mastotermitis* sp. nov. and *Pa­piliotrema odontotermitis* fa, sp. nov. from the gut of the termites *Mastotermes dar­winiensis* and *Odontotermes obesus*. International Journal of Systematic and Evolu­tionary Microbiology 66: 4600–4608. https://doi.org/10.1099/ijsem.0.001397

Kurtzman CP (2007) Eleven new species of *Sugiyamaella* and *Candida* from forest habitats. FEMS yeast research 7: 1046–1063. [https://doi.org/10.1111/j.1567- 1364.2007.00224.x.ů-0](https://doi.org/10.1111/j.1567-%201364.2007.00224.x.ů-0)

Middelhoven WJ, Kurtzman CP (2007) Four novel yeasts from decaying organic matter: *Blastobotrys robertii* sp. nov., *Candida cretensis* sp. nov., *Candida scorzettiae* sp. nov. and *Candida vadensis* sp. nov. Antonie Van Leeuwenhoek 92: 233–244. https://doi. org/10.1007/s10482-007-9159-1
